# Cardiac muscle–restricted partial loss of *Nos1ap* expression has limited but significant impact on electrocardiographic features

**DOI:** 10.1093/g3journal/jkad208

**Published:** 2023-09-14

**Authors:** Alexa Smith, Dallas Auer, Morgan Johnson, Ernesto Sanchez, Holly Ross, Christopher Ward, Aravinda Chakravarti, Ashish Kapoor

**Affiliations:** Institute of Molecular Medicine, McGovern Medical School, University of Texas Health Science Center at Houston, Houston, TX 77030, USA; McKusick-Nathans Institute of Genetic Medicine, Johns Hopkins University School of Medicine, Baltimore, MD 21205, USA; Institute of Molecular Medicine, McGovern Medical School, University of Texas Health Science Center at Houston, Houston, TX 77030, USA; Institute of Molecular Medicine, McGovern Medical School, University of Texas Health Science Center at Houston, Houston, TX 77030, USA; McKusick-Nathans Institute of Genetic Medicine, Johns Hopkins University School of Medicine, Baltimore, MD 21205, USA; Department of Molecular Physiology and Biophysics, Baylor College of Medicine, Houston, TX 77030, USA; McKusick-Nathans Institute of Genetic Medicine, Johns Hopkins University School of Medicine, Baltimore, MD 21205, USA; Center for Human Genetics and Genomics, New York University School of Medicine, New York, NY 10016, USA; Institute of Molecular Medicine, McGovern Medical School, University of Texas Health Science Center at Houston, Houston, TX 77030, USA; McKusick-Nathans Institute of Genetic Medicine, Johns Hopkins University School of Medicine, Baltimore, MD 21205, USA

**Keywords:** Nos1ap, knockout mouse, QT interval, electrocardiogram, echocardiogram

## Abstract

Genome-wide association studies have identified sequence polymorphisms in a functional enhancer of the *NOS1AP* gene as the most common genetic regulator of QT interval and human cardiac *NOS1AP* gene expression in the general population. Functional studies based on in vitro overexpression in murine cardiomyocytes and ex vivo knockdown in zebrafish embryonic hearts, by us and others, have also demonstrated that NOS1AP expression levels can alter cellular electrophysiology. Here, to explore the role of *NOS1AP* in cardiac electrophysiology at an organismal level, we generated and characterized constitutive and heart muscle–restricted *Nos1ap* knockout mice to assess whether *NOS1AP* disruption alters the QT interval in vivo. Constitutive loss of *Nos1ap* led to genetic background-dependent variable lethality at or right before birth. Heart muscle–restricted *Nos1ap* knockout, generated using cardiac-specific alpha-myosin heavy chain promoter-driven tamoxifen-inducible Cre, resulted in tissue-level *Nos1ap* expression reduced by half. This partial loss of expression had no detectable effect on the QT interval or other electrocardiographic and echocardiographic parameters, except for a small but significant reduction in the QRS interval. Given that challenges associated with defining the end of the T wave on murine electrocardiogram can limit identification of subtle effects on the QT interval and that common noncoding *NOS1AP* variants are also associated with the QRS interval, our findings support the role of *NOS1AP* in regulation of the cardiac electrical cycle.

## Teaser

To explore the role of *NOS1AP* in cardiac electrophysiology and QT interval regulation *in vivo*, Smith et al. generated constitutive and heart muscle-restricted *Nos1ap* knockout mice. Constitutive loss of Nos1ap led to near-complete lethality and had no major impact on the embryonic heart transcriptome. Heart muscle-restricted knockout, generated using cardiac specific alpha-myosin heavy chain promoter-driven tamoxifen-inducible Cre, reduced tissue-level expression by half, had no detectable effect on the QT interval, but led to a small and significant reduction in the QRS interval.

## Introduction

The electrocardiographic QT interval (QTi hereafter), an index of ventricular repolarization (Nerbonne and Kass 2005), is a clinically relevant, heritable quantitative trait associated with cardiovascular disease in the general population (Straus *et al.* 2006). Prolongation or shortening of the QTi, owing to underlying pathology, genetic variants, or adverse drug reactions, can lead to life-threatening arrhythmias and sudden cardiac death (SCD). QTi, the time between the start of the Q wave and the end of the T wave in an electrocardiogram (ECG), is a genetic trait with a heritability of 35%, also correlated with age, sex, and heart rate (Newton-Cheh *et al.* 2005). Beyond the rare, high penetrance, coding mutations leading to phenotypic extremes of QTi in subjects with Mendelian long QT (LQTS), short QT, or Brugada syndromes (Schwartz *et al.* 2013; Campuzano *et al.* 2018), common DNA sequence variation is a major source of QTi variation in the general population. Genome-wide association studies (GWAS) of QTi have mapped at least 35 common variant-based loci (Arking *et al.* 2006; Newton-Cheh *et al.* 2009; Pfeufer *et al.* 2009; Arking *et al.* 2014) contributing to its heritability, and, among them, the locus with the largest contribution (∼1.5% of phenotype variation) includes the *NOS1AP* gene on chromosome 1q23. Although the functional role of *NOS1AP* in cardiac repolarization is not established, QTi-associated common variants at the *NOS1AP* locus are also associated with an increased risk of SCD in the general population (Eijgelsheim *et al.* 2009; Kao *et al.* 2009) and are genetic modifiers of cardiac outcomes in subjects with LQTS (Schwartz *et al.* 2018). Furthermore, QTi-associated *NOS1AP* variants are also associated with the QRS interval, representing ventricular depolarization on an ECG, but the same sequence variants often act in the opposite direction (Sotoodehnia *et al.* 2010; Arking *et al.* 2014).

Nitric oxide synthase 1adaptor protein (NOS1AP), initially called carboxy-terminal PDZ ligand of neuronal (CAPON) NOS, is the C-terminal PDZ ligand of NOS1/nNOS and was originally cloned from a rat hippocampal cDNA library (Jaffrey *et al.* 1998). Nearly all biochemical characterization of *NOS1AP* function so far has been in in vitro and ex vivo systems, largely based on neuronal cell lines and tissue lysates (Wang *et al.* 2016), with very limited knowledge about its cardiac function. A relationship between the nitric oxide synthase pathway and cardiac repolarization was not recognized before the QTi GWAS mapping (Arking *et al.* 2006). Invoking a *cis*-regulatory mechanistic hypothesis, we identified a functional enhancer variant underlying the QTi GWAS signal that further influenced *NOS1AP* cardiac transcript expression (Kapoor *et al.* 2014). We also demonstrated that overexpression of long and short isoforms of human *NOS1AP* in neonatal rat ventricular myocytes (NRVMs) led to shortened action potential duration (APD), a potential cellular correlate of QTi (Kapoor *et al.* 2014). Similarly, others showed that overexpression of *Nos1ap* in guinea pig ventricular myocytes led to reduced APD via inhibition of L-type calcium channels and activation of delayed rectifier potassium channels (Chang *et al.* 2008). In contrast, optical mapping of excised whole hearts from developing zebrafish embryos with morpholino-based knockdown of *nos1ap* showed shortened APD (Milan *et al.* 2009). Although, directionally inconsistent, these studies indicated that *NOS1AP* expression levels can alter cellular electrophysiology. The differences observed could be simply due to differences in model systems or indicate that both gain and loss of *NOS1AP* can misregulate its functional complexes.

In an effort to explore the role of *NOS1AP* in cardiac electrophysiology at an organismal level, and to assess whether *NOS1AP* disruption alters the QTi in vivo, a critical knowledge gap, here we have generated and characterized constitutive and heart muscle–restricted *Nos1ap* knockout mice. In this paper, we report that constitutive loss of *Nos1ap* leads to near-complete lethality at or right before birth, that constitutive loss of *Nos1ap* has no major impact on the embryonic heart transcriptome, that heart muscle–restricted *Nos1ap* knockout, generated using cardiac-specific alpha-myosin heavy chain (αMHC) promoter-driven tamoxifen-inducible Cre (Sohal *et al.* 2001), reduces tissue-level *Nos1ap* expression by half, and that this partial loss of *Nos1ap* cardiac expression has no detectable effect on the QTi but leads to a small and significant reduction in the QRS interval.

## Materials and methods

### Generation of constitutive and conditional *Nos1ap* null mice

Targeted mouse embryonic (ES) cells with *Nos1ap* “knockout-first allele” (*Nos1ap*^tm1a^; reporter-tagged insertion with conditional potential) (Skarnes *et al.* 2011) derived from the parental ES cell line JM8A3.N1 (*A/a*; *Tyr*^+/+^) of the C57BL/6N strain (Pettitt *et al.* 2009) were purchased from the Knockout Mouse Project (KOMP) repository. Injections into albino blastocysts (C57BL/6 *a/a*; *Tyr*^c/c^) and generation of G0 chimeras with agouti coat color were performed at Texas A&M Institute of Genomic Medicine. Chimeric mice were crossed to C57BL/6J (*a/a*; *Tyr*^+/+^), and G1 mice were genotyped by PCR (Supplementary Table 1) to assess germline transmission. *Nos1ap*^+/tm1a^ mice were crossed with CMV-Cre (Jax stock #006054) (Schwenk *et al.* 1995) to generate reporter-tagged knockout mice (*lacZ*-tagged, *neo*^R^-deleted, *Nos1ap*-exon 4-deleted; *Nos1ap*^+/tm1b^) and crossed with ACTB-Flpe (Jax stock #005703) (Rodríguez *et al.* 2000) to generate Cre recombinase conditional knockout mice (*lacZ*-deleted, *neo*^R^-deleted, *Nos1ap*-exon 4-floxed; *Nos1ap*^+/tm1c^ or *Nos1ap*^+/fl^). Subsequently, *Nos1ap*^+/tm1c^ mice were crossed with CMV-Cre (Jax stock #006054) (Schwenk *et al.* 1995) to generate constitutive null mice (*lacZ*-deleted, *neo*^R^-deleted, *Nos1ap*-exon 4-deleted; *Nos1ap*^+/tm1d^ or *Nos1ap*^+/−^). All alleles were maintained by backcrossing to C57BL/6J, and mice with backcross generation number 10 or beyond (≥N10) were used for phenotyping of *Nos1ap* tm1c and tm1d alleles. All protocols for animal care, use, and euthanasia were reviewed and approved by the Institutional Animal Care and Use Committees at Johns Hopkins University (JHU), University of Texas Health Science Center at Houston (UTHealth), and Baylor College of Medicine (BCM) and were in accordance with the Association for Assessment and Accreditation of Laboratory Animal Care guidelines. All animals were fed a standard rodent chow ad libitum. Genomic DNA was isolated from tail tips of 3-week-old mice at weaning or from tail tips and left ventricle tissue of euthanized adult mice or from embryonic day 13.5 (E13.5) embryos following standard methods. All mice were genotyped by PCR (see Supplementary Table 1 for primers and Supplementary Table 2 for amplicons; PCR conditions are available on request). Alleles tm1a, tm1b, and tm1d are Nos1ap protein null by design.

### RNA isolation and gene expression analyses

Adult mice were euthanized using inhaled isoflurane in a closed chamber, and dissected tissues were snap frozen in liquid nitrogen prior to storage at −80°C. Total RNA was extracted from ∼20-mg dry tissue using TRIzol (Invitrogen, MA, USA) following the manufacturer's instructions. DNase digestion and RNA cleanup were performed using the RNeasy Mini Kit and RNase-Free DNase Set (Qiagen, MD, USA), following the manufacturer's instructions. cDNA was synthesized by oligo-dT primed reverse transcription performed on 1 µg of total RNA using SuperScript III First-Strand Synthesis System (Invitrogen, MA, USA), following the manufacturer's instructions. Quantitative expression analysis of *Nos1ap* was performed using mouse-specific TaqMan Gene Expression Assay (Mm01290688_m1; mapping to exons 5 and 6) (Applied Biosystems, MA, USA). Real-time quantitative PCR (qPCR) was performed on a 7900HT Fast Real-Time PCR System or QuantStudio 5 Real-Time PCR System (Applied Biosystems, MA, USA) and analyzed using Sequence Detection System Software v.2.1 or QuantStudio Design and Analysis Software v.1.2 (Applied Biosystems, MA, USA). Expression was measured in technical triplicates, and the averages of the threshold cycle (*C_t_*) values were used for analysis. *Actb* expression, assessed using mouse *Actb* Endogenous Control TaqMan Gene Expression assay (Applied Biosystems, MA, USA), was used for normalization.

### Western blotting

Nos1ap expression was evaluated in mouse brain cortex lysates using commercially available rabbit polyclonal NOS1AP antibody (R-300, Santa Cruz Biotechnology, TX, USA). Adult mice were euthanized using inhaled isoflurane in a closed chamber, and dissected tissues were snap frozen in liquid nitrogen prior to storage at −80°C. Whole tissue protein extracts were prepared by cryogenic pulverization of ∼20 mg of tissue with Cellcrusher (Cellcrusher, OR, USA). Pulverized tissue was suspended in modified RIPA buffer supplemented with a protease inhibitor cocktail (Roche, IN, USA). Following sonication, tissue and cell debris were removed by centrifugation, and protein concentration was determined by Bio-Rad DC Protein Assay (Bio-Rad, CA, USA). Samples (75 µg) were denatured and analyzed by western blotting following standard methods (Laemmli 1970). Relative estimation of protein bands was performed using ImageJ software (Schneider *et al.* 2012). Our efforts of bacterial expression and purification of a full-length GST-/His-tagged NOS1AP protein failed, largely due to cytotoxicity, because of which we did not have a purified NOS1AP protein to run as a positive control.

### RNA-seq library preparation, sequencing, and analyses

RNA-seq was performed in E13.5 heart tissues from 5 *Nos1ap*^+/+^ and 5 *Nos1ap*^−/−^ males. Total RNA was isolated from E13.5 heart tissue using RNeasy Mini Kit following the manufacturers’ recommendations (Qiagen, MD, USA) that included the on-column DNase digestion using RNase-Free DNase set (Qiagen, MD, USA). KAPA Stranded mRNA-Seq Kit (KAPA Biosystems, MA, USA) was used to generate indexed Illumina platform sequencing libraries. Briefly, polyA RNA was captured from 1 µg of total RNA using magnetic oligo-dT beads. After elution from the magnetic beads, polyA RNA was fragmented to generate inserts ranging in size from 100 to 200 bp, followed by random priming and reverse transcription to generate double-stranded cDNA. Next, after performing a 1.8× SPRI cleanup using AMPure XP beads (Agencourt, IN, USA), dAMP was added to 3′-ends of the cDNA fragments followed by ligation with indexed 3′-dTMP Illumina TruSeq adapters. Ligated fragments were subsequently size selected using PEG/NaCl SPRI solution and underwent PCR amplification (12 cycles) to generate the sequencing libraries. After performing a 1× SPRI cleanup using AMPure XP beads (Agencourt, IN, USA), a sample from each library was used to assess library fragment size distribution by electrophoresis using BioAnalyzer High Sensitivity DNA Assay (Agilent Technologies, CA, USA) and to assess library concentration by qPCR using KAPA Library Quantification Kit (KAPA Biosystems, MA, USA). Equimolar amounts of libraries were pooled and sequenced on an Illumina HiSeq 2500 instrument using standard protocols for paired-end 100-bp sequencing with a desired sequencing depth of ∼30 million paired-end reads per library. Paired-end read fastq files were quality checked using FASTQC (version 0.11.5) (http://www.bioinformatics.babraham.ac.uk/projects/fastqc/) and then processed using Trimmomatic (version 0.36) (Bolger *et al.* 2014), for removing adapters and other Illumina-specific sequences from the reads and for performing a sliding window–based trimming of low-quality bases from each read (ILLUMINACLIP:TruSeq3-PE-2.fa:2:30:10:1:TRUE LEADING:3 TRAILING:3 SLIDINGWINDOW:4:15 MINLEN:36). For estimating gene and isoform expression levels, we first extracted reference transcript sequences from the mouse genome (GRCm38, primary assembly) based on the GENCODE (http://www.gencodegenes.org/mouse_releases/current.html) primary assembly gene annotation (release M10) and built STAR aligner (Dobin *et al.* 2013) indices using the RSEM software package (version 1.2.31) (Li and Dewey 2011). Trimmed paired-end reads from each sample were then aligned to the reference transcript sequences by calling the STAR aligner within RSEM and using alignment parameters from the ENCODE STAR-RSEM long RNA-seq pipeline (--outSAMunmapped Within --outFilterType BySJout --outSAMattributes NH HI AS NM MD --outFilterMultimapNmax 20 --outFilterMismatchNmax 999 --outFilterMismatchNoverLmax 0.04 --alignIntronMin 20 --alignIntronMax 1000000 --alignMatesGapMax 1000000 --alignSJoverhangMin 8 --alignSJDBoverhangMin 1 --sjdbScore 1 --quantMode TranscriptomeSAM). Gene and isoform expression levels were then estimated in each sample from these transcriptome alignments using RSEM, keeping in mind the strandedness of the prepared RNA-seq libraries (--forward-prob 0.0). Gene-level read count data generated by RSEM were compared between wild-type and mutant mice to assess differential gene expression using DESeq (version 1.24.0) (Anders and Huber 2010). Only those genes where the sum of read counts across the 10 samples was >1 were retained for differential gene expression analysis. Although release M10 of the GENCODE primary assembly gene annotation has 48,526 genes, we limited differential gene expression comparison to only protein-coding genes (22,098). To address multiple hypothesis testing, observed *P*-values were adjusted based on the Benjamini–Hochberg false discovery rate (FDR) procedure (Benjamini and Hochberg 1995; Yekutieli and Benjamini 1999). All data have been deposited in NCBI's GEO and are accessible at GEO Series accession number GSE210266.

### Cardiac muscle–restricted *Nos1ap* loss of expression

To generate tamoxifen-inducible 0, 1, or 2 copy losses of *Nos1ap* in cardiac muscle, we utilized the mouse cardiac-specific αMHC promoter-driven tamoxifen-inducible Cre recombinase transgenic line (αMHC-MerCreMer; Jax stock #005657) (Sohal *et al.* 2001). *Nos1ap*^+/fl^; +/+ mice were crossed with *Nos1ap*^+/fl^; +/*Tg*^αMHC−MerCreMer^ mice to generate *Nos1ap*^+/+^, *Nos1ap*^+/fl^, and *Nos1ap*^fl/fl^ mice with and without αMHC-MerCreMer transgene. To induce Cre recombinase, 4-week-old mice were treated with tamoxifen (Sigma, MO, USA) by intraperitoneal (IP) injection once a day for 5 continuous days at a dose of 20 mg/kg per day (Sohal *et al.* 2001). Tamoxifen stock solution was prepared weekly by dissolving 50-mg tamoxifen in 10 ml of corn oil (Sigma, MO, USA) and stored at 4°C. Following a 1-week gap postinjections, a small number of mice were euthanized to assess Cre recombinase–mediated deletion of floxed allele by PCR genotyping of tail and heart genomic DNA samples.

### Electrocardiographic and echocardiographic measurements

ECG in conscious mice was performed using ECGenie System (Mouse Specifics, MA, USA), with data acquisition using LabChart (ADIstruments, CO, USA) and automated measurements using EzCG Analysis software (Mouse Specifics, MA, USA) following the manufacturer's instructions. Briefly, animals were placed on the recording platform to acclimate for 5–10 min before starting data collection. Data were collected for ∼10 min at a sampling rate of 2,000/s and the following filter settings: 3-Hz high pass, 100-Hz low pass, 60-Hz notch, and mains filter. At least 3 different segments of ECG signals, each with 20 or more heartbeats, when each animal was positioned so that the paws were touching both the inner and the outer area of the lead plate with the same lead orientation, were exported and analyzed as individual data points to report various ECG indices. ECGs in anesthetized mice were captured using Rodent Surgical Monitor+ (Indus Instruments, TX, USA) and PowerLab 4/35 (ADInstruments, CO, USA), with automated measurements using LabChart 8 software (ADInstruments, CO, USA). Briefly, animals were kept anesthetized by inhaled isoflurane delivered in oxygen (induction at 2.5–4%, maintenance at 1.5% isoflurane) via a nose cone and placed supine on the monitoring platform with paws taped in contact with electrodes for recording ECG waveforms. The monitoring platform was heated and feedback controlled via rectal thermometer to maintain thermal homeostasis (36–38°C) during the ∼25-min recording session. Following a 5-min baseline recording, isoproterenol hydrochloride (USP, MD, USA) at 1- or 5-mg/kg dose was delivered via IP injection for pharmacological challenge. Postinjection measurement was collected for the next 20 min. ECG signals in the 5-min baseline recording were averaged to assess various ECG indices. Similarly, ECG signals corresponding to the 5-min postinjection window from the start of the 10th to the start of the 15th minute were averaged. ECG measurements for the 2 isoproterenol hydrochloride doses were separated by 5 days. To facilitate signal analysis, a digital 5-Hz high pass filter was applied, and the in-built ECG analysis suite was used for identifying ECG beats and analysis of ECG parameters following the manufacturer's instructions. The QTi was corrected (QTc) for heart rate as described earlier (Mitchell *et al.* 1998). Given the recent findings on appropriateness of heart rate–based QTi correction in rodents (Mulla *et al.* 2022), uncorrected QTi was also used for comparisons. Two days after the second ECG measurement, echocardiography was performed using a Vevo 2100 system (FUJIFILM VisualSonics, WA, USA) with MS550S transducer, following the manufacturer's instructions. Briefly, animals were anesthetized by inhaled isoflurane and maintained at a body temperature between 36 and 38°C as described above. Animals and ultrasound transducer probe were positioned to facilitate short-axis imaging of left ventricle at the level of papillary muscles, and B-mode and M-mode images were acquired. Quantification of left ventricle structure and function from M-mode images was performed using the manufacturer's software that permitted assessment of ventricle wall thickness, inner diameter, and derived measures including ejection fraction (EF) and fractional shortening (FS).

### Statistical analyses

Counts data were compared using *χ*^2^ contingency tests. Student's *t*-test was utilized for comparing mean values between groups. Bonferroni correction was used to adjust for multiple testing. Multiple linear regression was used to evaluate the effect of multiple predictors on ECG parameters.

## Results

### Generation of constitutive and conditional *Nos1ap* knockout mice

**Fig. 1.**
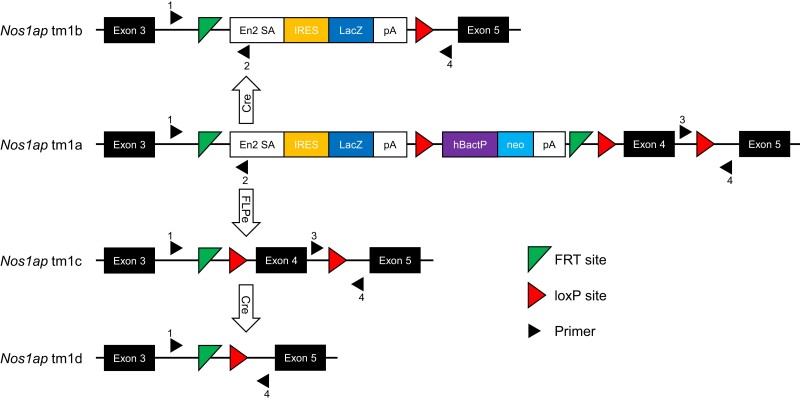
Targeted and derived *Nos1ap* alleles. The “knockout-first” (tm1a) allele contains an internal ribosome entry site (IRES):*lacZ* trapping cassette and a floxed human beta actin (hBactP) promoter-driven *neo* cassette inserted into intron 3 of *Nos1ap*. A splice acceptor sequence from Engrailed 2 (En2 SA) and poly-A (pA) transcription termination signals disrupt *Nos1ap* expression while expressing *lacZ* under the control of the endogenous promoter. Exposure to Cre recombinase mediates conversion of tm1a to tm1b allele to generate a nonconditional *lacZ*-tagged null allele without the *neo* cassette and without the critical region (exon 4). Exposure to FLPe recombinase mediates conversion of tm1a to tm1c allele to generate a conditional floxed allele, which on further exposure to Cre recombinase can generate either the constitutive null allele (tm1d) or a tissue-restricted *Nos1ap* knockout. Triangles, numbered 1–4, indicate primers used for PCR genotyping; see Supplementary Tables 1 and 2 for details. Adapted from Ryder *et al*. (2013).

**Fig. 2.**
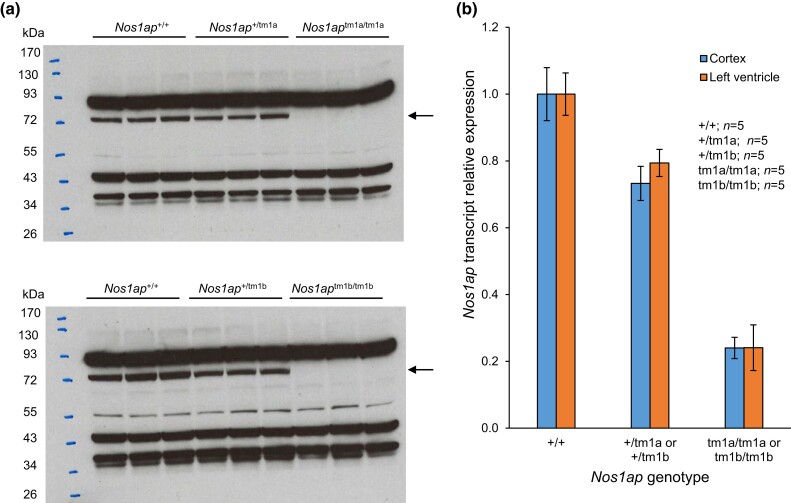
Loss of Nos1ap protein and transcript in *Nos1ap* tm1a and tm1b allele carriers. a) Western blot of brain cortex lysates from adult mouse using rabbit polyclonal NOS1AP antibody shows complete absence of Nos1ap protein (immunoreactive band indicated by arrow) in tm1a (top) and tm1b (bottom) homozygotes and reduced protein levels in tm1a (top) and tm1b (bottom) heterozygotes as compared to wild-type mice. Other immunoreactive bands likely indicating nonspecific binding were also observed. b) Compared to wild-type mice, tm1a or tm1b heterozygotes and tm1a or tm1b homozygotes had significantly reduced *Nos1ap* transcript expression in adult brain cortex and left ventricle tissues. Error bars: SEM.

Starting with mouse ES cells targeting exon 4 of *Nos1ap* (NM_001109985) with the “knockout-first allele” (Skarnes *et al.* 2011), purchased from the KOMP repository, reporter-tagged *Nos1ap* knockout mice with conditional potential (*Nos1ap*^+/tm1a^) were generated by blastocyst injection and germline transmission. The flexible design of the tm1a allele was exploited to generate nonconditional reporter-tagged knockout (reporter-tagged and exon 4-deleted; *Nos1ap*^+/tm1b^), Cre recombinase conditional knockout (exon 4-floxed; *Nos1ap*^+/tm1c^ or *Nos1ap*^+/fl^), and constitutive null (exon 4-deleted; *Nos1ap*^+/tm1d^ or *Nos1ap*^+/−^) mice by crossing with CMV-Cre (Schwenk *et al.* 1995) and ACTB-Flpe (Rodríguez *et al.* 2000) mice (Fig. 1). Targeted (tm1a) and derived (tm1b, tm1c, and tm1d) alleles were maintained in the C57BL/6J background. We used *Nos1ap*^+/tm1c^ and *Nos1ap*^+/tm1d^ mice from backcross generation ≥N10 in phenotypic studies. Alleles tm1a and tm1b are expected to be Nos1ap protein null by design due to the insertion of a transgene cassette containing the Engrailed 2 splice acceptor, internal ribosome entry site, *lacZ* open-reading frame, and polyadenylation signal between exons 3 and 4. Allele tm1d is also expected to be Nos1ap protein null due to exon 4 deletion-mediated frameshift that creates a premature termination codon leading to nonsense-mediated mRNA decay. Western blotting of adult brain cortex tissue lysates showed complete absence of Nos1ap protein in *Nos1ap*^tm1a/tm1a^ and *Nos1ap*^tm1b/tm1b^ mice and a considerable decrease in protein levels in *Nos1ap*^+/tm1a^ and *Nos1ap*^+/tm1b^ mice compared to wild type (Fig. 2; Supplementary Fig. 1). Similarly, compared to wild type, *Nos1ap* transcript expression in adult brain cortex tissue as measured by real-time qPCR on cDNA was reduced to 73% (*P* = 0.01) in *Nos1ap*^+/tm1a^ and *Nos1ap*^+/tm1b^ mice and to 24% (*P* = 7.88 × 10^−8^) in *Nos1ap*^tm1a/tm1a^ and *Nos1ap*^tm1b/tm1b^ mice (Fig. 2; Supplementary Fig. 1). The corresponding values for *Nos1ap* transcript expression in adult left ventricle tissue were 79% (*P* = 0.01) and 24% (*P* = 8.47 × 10^−6^) (Fig. 2; Supplementary Fig. 1).

### Constitutive loss of *Nos1ap* leads to near-complete lethality

To generate homozygous *Nos1ap* constitutive null mice, free of *lacZ*, and *neo* cassette, *Nos1ap*^+/tm1d^ mice were intercrossed. With increasing backcross generation numbers, *Nos1ap*^+/tm1d^ intercrosses were performed at 2 different locations: first at JHU and then at UTHealth. Among *Nos1ap*^+/tm1d^ intercrosses at JHU (parental mice from N8 to N11), 9.6, 7.5, and 25.2% of all mice were *Nos1ap*^tm1d/tm1d^ at weaning (P21), birth (P0), and E13.5, respectively (Table 1). Among *Nos1ap*^+/tm1d^ intercrosses at UTHealth (parental mice from N14 to N15), 3.4% of all mice were *Nos1ap*^tm1d/tm1d^ at P21 (Table 1). With an expectation of 25% mice being null homozygotes under Mendelian segregation, a significant drop in counts observed at P21 (*P* = 7.61 × 10^−7^ JHU; *P* = 1.53 × 10^−5^ UTHealth) and P0 (*P* = 1.04 × 10^−4^), but not at E13.5 (*P* = 0.09) (Table 1), indicates that complete loss of *Nos1ap* in *Nos1ap*^tm1d/tm1d^ mice leads to near-complete lethality at birth or during late gestation (after E13.5).

**Table 1.** Genotype distribution from *Nos1ap* constitutive null intercrosses.

	E13.5 (%)*^a^*	P0 (%)*^a^*	P21 (%)*^a^*	P21 (%)*^b^*
+/+	37 (33.3)	35 (32.7)	70 (32.0)	31 (35.6)
+/−	46 (41.4)	64 (59.8)	128 (58.4)	53 (60.9)
−/−	28 (25.2)	8 (7.5)	21 (9.6)	3 (3.4)
*χ* ^2^	4.71	17.75	28.18	22.17
*P*	0.09	1.04 × 10^−4^	7.61 × 10^−7^	1.53 × 10^−5^

At JHU.

At UTHealth.

### Constitutive loss of *Nos1ap* has no significant impact on E13.5 heart transcriptome

To evaluate the *molecular* consequences of *Nos1ap* loss that might lead to embryonic lethality, we assessed differential gene expression between E13.5 heart transcriptomes from wild-type and *Nos1ap*^tm1d/tm1d^ mice. We performed stranded mRNA-seq (Mortazavi *et al.* 2008) in E13.5 heart tissues dissected from 5 wild-type and 5 *Nos1ap*^tm1d/tm1d^ male mice. Paired-end 100-bp sequencing was performed on an Illumina HiSeq 2500 with a desired depth of ∼30 million paired-end reads per sample. Low-quality bases and Illumina adapter sequences were removed from the generated reads using Trimmomatic (Bolger *et al.* 2014). RSEM (Li and Dewey 2011) was used for estimation of gene and isoform expression level that included mapping of trimmed paired-end reads to the mouse genome and GENCODE transcripts using STAR aligner (Dobin *et al.* 2013). Gene-level read count data from 5 wild-type and 5 mutants were compared to assess differential gene expression using DESeq (Anders and Huber 2010). At a FDR of 1% and an absolute log_2_-fold change over 1, only 2 genes (*Mt2* and *Gm7694*) were differentially expressed (Supplementary Data Set 1 and Supplementary Fig. 2), beyond that expected at *Nos1ap*. Compared to wild type mice, *Nos1ap* expression was significantly reduced (0.3×; *P* = 2.9 × 10^−31^), *Mt2* expression was significantly increased (2.3×; *P* = 4.2 × 10^−8^), and *Gm7694* expression was significantly reduced (0.4×; *P* = 8.5 × 10^−39^) in *Nos1ap*^tm1d/tm1d^ mice. These findings indicate that constitutive loss of *Nos1ap* has no major widespread impact on the E13.5 heart transcriptome.

### Cardiac muscle–restricted αMHC promoter-driven tamoxifen-inducible Cre leads to partial loss of expression in *Nos1ap* floxed mice

**Fig. 3.**
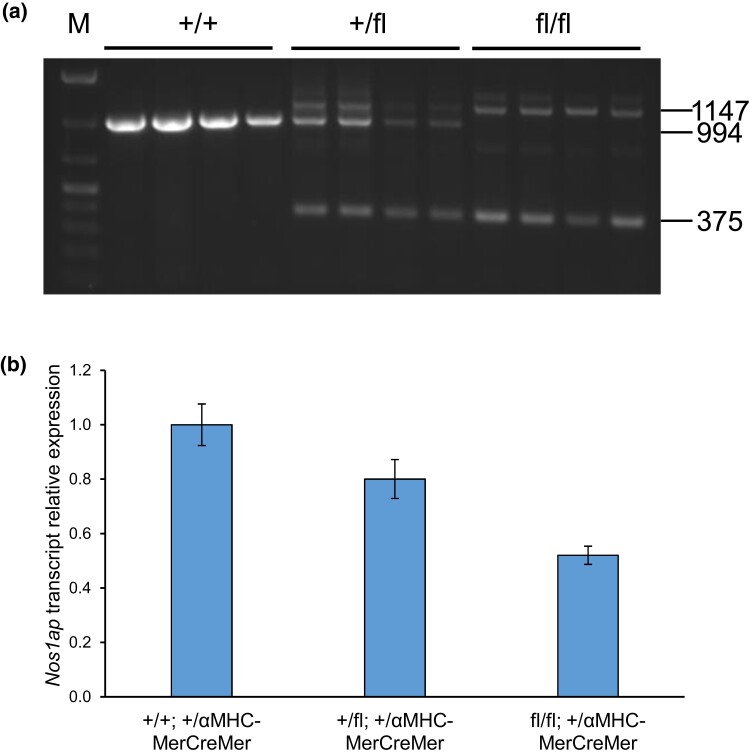
Cardiac αMHC promoter-driven tamoxifen-inducible Cre recombinase leads to excision of floxed allele and loss of *Nos1ap* expression in left ventricle tissue. a) Following tamoxifen IP injections to induce Cre recombinase activity, agarose gel electrophoresis of amplicons generated by PCR at the *Nos1ap* locus using left ventricle tissue genomic DNA from *Nos1ap*^+/+^, *Nos1ap*^+/fl^, and *Nos1ap*^fl/fl^ mice, all with tamoxifen-inducible αMHC-MerCreMer transgene, shows wild-type (+) allele amplicon (994 bp), floxed (fl) allele amplicon (1,147 bp), and floxed excision allele amplicon (375 bp). M: DNA ladder. b) Compared to wild-type mice, excision of the *Nos1ap* floxed allele leads to reduced *Nos1ap* transcript expression in left ventricle tissue of floxed heterozygotes and homozygotes.

Given the near-complete lethality observed in *Nos1ap* constitutive null homozygous mice at birth or during late gestation, we evaluated the effects of cardiac muscle–restricted *Nos1ap* loss using the *Nos1ap* floxed mice and cardiac αMHC promoter-driven tamoxifen-inducible Cre recombinase (MerCreMer) transgenic mice (αMHC-MerCreMer) (Sohal *et al.* 2001). Crosses were set up between *Nos1ap*^+/fl^; +/+ and *Nos1ap*^+/fl^; +/*Tg*^αMHC−MerCreMer^ mice to generate *Nos1ap*^+/+^, *Nos1ap*^+/fl^, and *Nos1ap*^fl/fl^ mice with and without αMHC-MerCreMer transgene. All 6 genotypes were observed at expected proportions at weaning (*P* = 0.75; Supplementary Table 3). Tamoxifen IP injections were performed in 4-week-old animals for 5 consecutive days at a dosage of 20 mg/kg per day to induce Cre recombinase activity (Sohal *et al.* 2001) and were followed by a 1-week waiting period before any experimentation. *Nos1ap*^+/+^, *Nos1ap*^+/fl^, and *Nos1ap*^fl/fl^ mice with and without αMHC-MerCreMer were viable and normal in size and did not display any gross physical or behavioral abnormalities (data not shown). PCR genotyping of genomic DNA isolated from left ventricle and tail (control) tissue in a subset of animals showed cardiac-restricted excision of the floxed allele, with no “leaky” Cre recombinase activity in tail tissue (Fig. 3; Supplementary Fig. 3). As *αMHC* promoter activity is mostly limited to cardiomyocytes, the unexcised floxed allele PCR band derived from other cell types in left ventricle tissue was also observed in *Nos1ap*^fl^ and *Tg*^αMHC−MerCreMer^ carriers (Fig. 3). The effect of Cre-mediated, floxed allele excision on *Nos1ap* transcript expression was evaluated by real-time qPCR using cDNA generated from left ventricle tissue harvested at terminal euthanasia. Among αMHC-MerCreMer transgene positive animals, compared to wild type (*n* = 6), *Nos1ap* expression in left ventricle tissue was reduced to 80% (*P* = 0.09) and 52% (*P* = 1.85 × 10^−4^) in *Nos1ap*^+/fl^ (*n* = 6) and *Nos1ap*^fl/fl^ (*n* = 6) mice, respectively (Fig. 3). Among αMHC-MerCreMer transgene negative animals, no significant difference in *Nos1ap* left ventricle expression was observed among *Nos1ap*^+/+^, *Nos1ap*^+/fl^ (*P* = 0.91), and *Nos1ap*^fl/fl^ (*P* = 0.50) mice (Supplementary Fig. 4).

### Partial loss of *Nos1ap* cardiac expression impacts QRS interval

Starting at 6 weeks of age, a week after tamoxifen IP injections, paw contact–based awake ECG recordings were carried out every 2 weeks until 24 weeks in *Nos1ap*^+/+^, *Nos1ap*^+/fl^, and *Nos1ap*^fl/fl^ mice, with and without αMHC-MerCreMer transgene, and every 4 weeks thereafter until 48 weeks in *Nos1ap*^+/+^, *Nos1ap*^+/fl^, and *Nos1ap*^fl/fl^ mice with αMHC-MerCreMer transgene. Representative images of ECG signals from multiple heartbeats and ensemble average ECG trace from awake ECG recordings are shown in Supplementary Data Set 2. At each time point, ECGs were recorded in 10 or more animals (in nearly equal sex ratios) for each of the 6 genotypes (Supplementary Table 4), and age-, sex-, and genotype-dependent effects on QTc and QT (Mitchell *et al.* 1998; Mulla *et al.* 2022) and other ECG parameters were assessed using multiple linear regression. Overall, based on the predictor variables used (age in weeks, female sex, *Nos1ap*^+/fl^, and *Nos1ap*^fl/fl^), the variance explained for QTc and QT remained low (QTc: adjusted *R*^2^ of 7.17 and 3.10% with and without αMHC-MerCreMer set, Supplementary Tables 5 and 7; QT: adjusted *R*^2^ of 5.77 and 1.56% with and without αMHC-MerCreMer set, Supplementary Tables 6 and 8). Besides small, but consistent and significant age-dependent effects on QTc (*β* = 0.03, *P* = 1.09 × 10^−20^ in with the αMHC-MerCreMer set, Supplementary Table 5; *β* = 0.04, *P* = 3.30 × 10^−5^ in without the αMHC-MerCreMer set, Supplementary Table 7) and sex-dependent effects on QTc (*β* = −0.66, *P* = 7.71 × 10^−15^ in with the αMHC-MerCreMer set, Supplementary Table 5; *β* = −0.19, *P* = 0.08 in without the αMHC-MerCreMer set, Supplementary Table 7), relative to wild-type, absolute genotype-dependent effects from *Nos1ap*^+/fl^; +/αMHC-MerCreMer (*β* = 0.11 ms) or *Nos1ap*^fl/fl^; +/αMHC-MerCreMer (*β* = 0.34 ms) genotypes (Supplementary Table 5) and *Nos1ap*^+/fl^; +/+ (*β* = 0.66 ms) or *Nos1ap*^fl/fl^; +/+ (*β* = 0.63 ms) genotypes (Supplementary Table 7) were negligible. Similarly, beyond small, consistent and significant age-dependent effects on QT (*β* = 0.03, *P* = 1.49 × 10^−18^ in with the αMHC-MerCreMer set, Supplementary Table 6; *β* = 0.03, *P* = 0.002 in without the αMHC-MerCreMer set, Supplementary Table 8) and sex-dependent effects on QT (*β* = −0.38, *P* = 1.67 × 10^−6^ in with αMHC-MerCreMer set, Supplementary Table 6; *β* = −0.19, *P* = 0.07 in without αMHC-MerCreMer set, Supplementary Table 8), relative to wild-type, absolute genotype-dependent effects from *Nos1ap*^+/fl^; +/αMHC-MerCreMer (*β* = −0.16 ms) or *Nos1ap*^fl/fl^; +/αMHC-MerCreMer (*β* = 0.25 ms) genotypes (Supplementary Table 6) and *Nos1ap*^+/fl^; +/+ (*β* = 0.37 ms) or *Nos1ap*^fl/fl^; +/+ (*β* = 0.48 ms) genotypes (Supplementary Table 8) were negligible. These data indicate that loss of *Nos1ap* cardiac expression had no major impact on ventricular repolarization. None of the other ECG parameters assessed (RR, PR, and QRS intervals) varied significantly across genotypes (Supplementary Data Set 3).

**Fig. 4.**
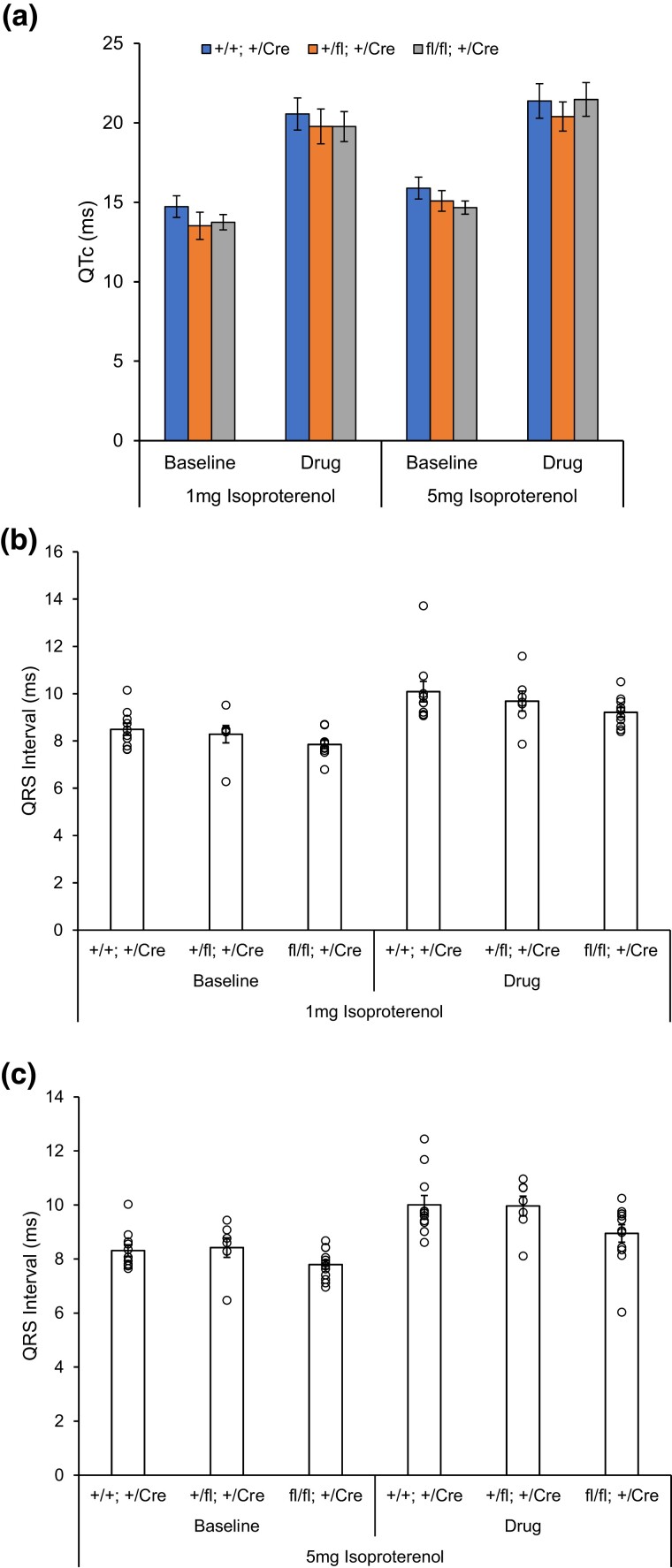
Cardiac muscle–specific loss of *Nos1ap* expression reduces QRS interval without a significant impact on QT interval. a) Mean heart rate–corrected QT interval (QTc) from ECG recording under anesthesia at baseline and after injecting 1- or 5-mg/kg body weight doses of isoproterenol in *Nos1ap*^+/+^, *Nos1ap*^+/fl^, and *Nos1ap*^fl/fl^ mice, all with tamoxifen-inducible αMHC-MerCreMer transgene, shows no significant difference across genotypes. Error bars: SEM. b, c) Mean (bar chart) and individual (dot plot) QRS intervals from ECG recording under anesthesia at baseline and after injecting 1- (b) or 5-mg/kg (c) body weight doses of isoproterenol in *Nos1ap*^+/+^, *Nos1ap*^+/fl^, and *Nos1ap*^fl/fl^ mice, all with tamoxifen-inducible αMHC-MerCreMer transgene, show a small, but significant reduction in QRS interval in floxed homozygotes. Error bars: SEM.

Given that the paw contact–based awake ECG recordings (above) may fail to detect subtle effects due to reduced measurement sensitivity and to collect longer ECG recordings to evaluate the effect of an acute pharmacological challenge, we transitioned to ECG measurements in anesthetized animals using surface electrodes. The set of animals with 48 weeks of age awake ECG recording above (*Nos1ap*^+/+^, *Nos1ap*^+/fl^, and *Nos1ap*^fl/fl^ with αMHC-MerCreMer; Supplementary Table 4) were evaluated further by ECG and echocardiography under anesthesia, followed by terminal euthanasia to harvest tissues for gene expression studies. Surface ECG recordings were performed at baseline and after IP injections of isoproterenol, a beta-adrenergic agonist, at 1 and 5 mg/kg doses. Representative images of ECG signals from multiple heartbeats and ensemble average ECG trace at baseline and after isoproterenol injections from anesthetized ECG recordings are shown in Supplementary Data Set 2. Sex-, drug exposure-, and genotype-dependent effects on QTc and QT and other ECG parameters were assessed using multiple linear regression. However, no significant differences in QTc (Fig. 4) and QT (Supplementary Fig. 5) were observed from surface ECG recordings at baseline and under stress across the 3 genotypes, again indicating lack of a genotype-dependent effect on ventricular repolarization [QTc: Supplementary Table 9 (1-mg/kg drug) and Supplementary Table 11 (5-mg/kg drug); QT: Supplementary Table 10 (1-mg/kg drug) and Supplementary Table 12 (5-mg/kg drug)]. None of the other ECG parameters differed significantly across the 3 genotypes (Supplementary Fig. 6; Supplementary Data Set 3), except a small, but consistent trend of reduced QRS interval in *Nos1ap* floxed homozygotes [mean (± SEM) QRS interval 8.49 (± 0.25) ms vs 7.85 (± 0.18) ms at baseline (*P* = 0.05) and 10.09 (± 0.43) ms vs 9.21 (± 0.21) ms under stress (*P* = 0.08) for 1-mg/kg isoproterenol in wild-type and floxed homozygotes, respectively; 8.32 (± 0.21) ms vs 7.79 (± 0.16) ms at baseline (*P* = 0.06) and 10.01 (± 0.35) ms vs 8.95 (± 0.32) ms under stress (*P* = 0.04) for 5-mg/kg isoproterenol in wild-type and floxed homozygotes, respectively; Fig. 4, Supplementary Data Set 3]. Multiple linear regression model using 1-mg/kg isoproterenol drug exposure, sex, and genotypes as predictors explained 40.66% of the observed variance in QRS intervals (*P* = 4.94 × 10^−6^), with floxed homozygous genotype having a significant effect (*β* = −0.75, *P* = 0.014; Supplementary Table 13). Similarly, at 5-mg/kg isoproterenol drug exposure, QRS interval variance explained was 42.57% (*P* = 4.75 × 10^−7^), with floxed homozygous genotype having a significant effect (*β* = −0.78, *P* = 0.006; Supplementary Table 14).

To evaluate potential effects on heart structure and function, echocardiography was performed in anesthetized animals, where short-axis image of left ventricle was acquired in M-mode and analyzed for structure and function outcome measures. Besides a trend of reduced EF and FS observed in *Nos1ap* floxed allele carriers (wild-type vs floxed heterozygote: *P* = 0.032 for EF and *P* = 0.020 for FS; wild-type vs floxed homozygote: *P* = 0.014 for EF and *P* = 0.016 for FS; none significant after applying multiple testing correction) (Supplementary Fig. 7), none of the other echocardiographic parameters differed significantly across the 3 genotypes (Supplementary Data Set 3), indicating that loss of *Nos1ap* cardiac expression had no major impact on left ventricle structure and function.

## Discussion

Following the GWAS mapping of QTi near *NOS1AP* (Arking *et al.* 2006; Newton-Cheh *et al.* 2009; Pfeufer *et al.* 2009; Arking *et al.* 2014), we reported identification of a functional enhancer variant, among trait-associated variants, which acted as an expression quantitative trait locus and influenced human cardiac *NOS1AP* gene expression (Kapoor *et al.* 2014). Furthermore, in vitro and ex vivo expression perturbation studies, by us and others, showed that *NOS1AP* expression levels influenced APD (Chang *et al.* 2008; Milan *et al.* 2009; Kapoor *et al.* 2014), a cellular correlate of QTi. In parallel to our gene knockout approach described here, conditional *Nos1ap* overexpression transgenic mouse models have been generated by us (Auer *et al.* 2014) and others (Jänsch *et al.* 2023). We had reported generation of 3 independent lines of Cre recombinase conditional *Nos1ap* overexpression transgenic mice but did not evaluate them by electrocardiography or echocardiography (Auer *et al.* 2014). In a recently published study, Jansch *et al.* (2023) reported generation of a conditional transgenic mouse with heart-specific murine *Nos1ap* overexpression that led to a decrease in QT duration and shortening of APD, confirming the in vivo role of *Nos1ap* in cardiac electrophysiology. Together, these studies have identified *NOS1AP* as the most likely causal gene underlying the QTi GWAS signal on chromosome 1q23. Human NOS1AP protein (NP_055512; 506 amino acids) and mouse Nos1ap protein (NP_001103455; 503 amino acids) have a high level of sequence identity, suggestive of conserved function, with a consensus of 508 residues that has 481 identities (95%), 20 mismatches, and 7 gaps (Altschul *et al.* 1990). Our goal here was to generate *Nos1ap* knockout mice and assess the impact of *Nos1ap* in vivo loss of expression on QTi and other electrocardiographic features. Cardiac muscle–restricted partial loss of *Nos1ap* expression had no significant impact on QTi, but a small yet statistically significant reduction in QRS interval was observed, supporting the role of *NOS1AP* in the regulation of the cardiac electrical cycle. Furthermore, there was a trend towards reduced EF and FS by echocardiography, suggesting that cardiac muscle–restricted partial loss of *Nos1ap* expression led to a decline in left ventricle function.

Due to differences in ionic currents that generate different shapes of ventricular action potential in human and mouse (Nerbonne and Kass 2005), the ST segment is lacking in the mouse ECG and the amplitude of T wave is relatively small to the extent that existence of an actual T wave on mouse ECG is a matter of longstanding debate (Boukens *et al.* 2014). The small amplitude of T wave makes pinpointing its end, determined as the return of signal to the voltage corresponding to the mean isoelectric value, error-prone, and this reduced measurement accuracy can lead to failures in detecting subtle effects (London 2001). Thus, even though *Nos1ap* expression levels have been shown to alter APD in rat and guinea pig cardiomyocytes in vitro (Chang *et al.* 2008; Kapoor *et al.* 2014), detecting that effect at an organismal level as altered QTi on mouse ECG can be challenging (London 2001). In general, using gene knockout mice to evaluate effects on QTi has been a challenge. Even when specifically evaluating targeted deletions for well-established genes known to lead to LQTS (LQT1: *KCNQ1*; LQT2: *KCNH2*; and LQT3: *SCN5A*) in humans or genes underlying major repolarizing currents in mouse heart, there are several examples of either failure to find an effect, inconsistent results across studies or differences between in vivo and ex vivo/cellular electrophysiological measurements (Salama and London 2007). Lee *et al.* (2000) found no effect on QTc in *Kcnq1*^−/−^ mice, whereas Casimiro et al. (2001) reported prolonged QTc in *Kcnq1*^−/−^ mice but not in isolated hearts. Targeted deletion of a cardiac-specific transcript isoform of *Kcnh2*, known as *Merg1b*, led to no change in QTc in *Merg1b*^−/−^ mice (Lees-Miller *et al.* 2003), even though spontaneous and abrupt bradycardias were observed in mutants. Similarly, Papadatos *et al.* (2002) reported slow atrial and atrioventricular conduction in *Scn5a*^+/−^ mice, evidenced by prolongation of the P wave and the PR interval, but QTc remained unchanged (*Scn5a*^−/−^ is embryonic lethal). Among the genes regulating major repolarizing currents in mouse myocytes, targeted deletions of *Kncd2* (Guo *et al.* 2005), *Kcnd3* (Niwa *et al.* 2008), and *Knca4* (London *et al.* 1998) had no effects on QTc. Nonetheless, the vast majority of these knockouts had major effects on the *electrophysiology* in myocytes and/or isolated hearts and often led to significant changes in other ECG parameters.

Although the effect of common *NOS1AP* variants is largest for QTi, the same variants also show an attenuated association with QRS interval (Sotoodehnia *et al.* 2010; Arking *et al.* 2014), surprisingly in the opposite direction. The QRS interval, representing ventricular depolarization, is also known to be positively correlated with the QTc interval (*r* = 0.44), which represents ventricular repolarization (Holm *et al.* 2010). Analogously, overall, across all ECG measurements in conscious and anesthetized mice here, QRS and QTc intervals were positively correlated (*r* = 0.30 and *r* = 0.74 in awake and anesthetized mice, respectively). In addition, due to the lack of a plateau in mouse cardiac action potentials, the QRS complex on mouse ECG corresponds to the spread of ventricular depolarization and the early phase of ventricular repolarization (London 2001). Therefore, it is possible that the reduced QRS interval we are observing in heart muscle–restricted *Nos1ap* knockout mice is indicative of shortened ventricular repolarization, at least in the early phase. This observation aligns (in terms of directionality) with the optical mapping–based reduced APD reported from zebrafish excised hearts with morpholino-based *nos1ap* knockdown (Milan *et al.* 2009). As outlined below, there are other plausible reasons for not finding an overt QTi phenotype in our *Nos1ap* knockout mice, in addition to asking if a gene knockout is always an appropriate model to characterize its function (Rossi *et al.* 2015; El-Brolosy and Stainier 2017).

In this study, constitutive loss of *Nos1ap* in null homozygous mice (*Nos1ap*^tm1d/tm1d)^ led to near-complete lethality at a time point after E13.5, indicating an essential role in late embryonic development, organ maturation, or the process of birth. It remains unknown why this lethal phenotype was not fully penetrant, but there was a trend of increased penetrance as the backcross generation number for the heterozygous mice used in the intercrosses increased from N8 to N15 (9.6% vs 3.4% null homozygotes at weaning), probably indicating an effect of genetic background. Constitutive loss of *Nos1ap* in reporter-tagged knockout with conditional potential (*Nos1ap*^tm1a/tm1a^) and in nonconditional reporter-tagged knockout (*Nos1ap*^tm1b/tm1b^) also displayed near-complete lethality (7.7 and 8.8% null homozygotes at weaning for tm1a and tm1b, respectively) from intercrosses at earlier backcross generations (N2–N4 for tm1a and N5–N6 for tm1b). Taken together, across all 3 null alleles and backcross generations N2–N15, 8.0% of mice at weaning from intercrosses were null homozygotes, underscoring the embryonic or early postnatal lethality with constitutive loss of *Nos1ap*. These observations are at variance with the publicly available viability data from the International Mouse Phenotyping Consortium (IMPC; https://www.mousephenotype.org/) for *Nos1ap*^+/tm1b^ intercrosses that show expected Mendelian ratios for the 3 genotypes. A pure genetic background of our mice (C57BL/6J) vs the mixed genetic backgrounds of IMPC mice at the levels of test cross (germline transmission), tm1a to tm1b conversion (Cre-driver), and maintenance of alleles could potentially explain this difference and is consistent with the polygenic nature of QTi (Arking *et al.* 2006; Newton-Cheh *et al.* 2009; Pfeufer *et al.* 2009; Arking *et al.* 2014).

Although previous studies have not evaluated a role for *NOS1AP* in gene expression regulation, we explored E13.5 heart transcriptome with the aim to uncover molecular events that may underlie late embryonic or preweaning lethality observed in *Nos1ap* constitutive null homozygotes. However, we did not find any major impact on E13.5 heart transcriptome as assessed by RNA-seq and differential gene expression analysis. Given these data along with the presence of a functional mouse fetal heart before E13.5, we conclude that the cause of lethality is unrelated to cardiac development. Indeed, *NOS1AP* is widely expressed in several human and ES and adult tissues (Kapoor *et al.* 2014; GTEx Consortium *et al.* 2017), with the highest expression levels in brain tissues. Therefore, assessing the transcriptional molecular consequences of *Nos1ap* loss of function in additional tissues/organs remains critical.

We would like to highlight that our study is not the first report of a *Nos1ap* knockout mouse model. A previous study reported generation of *Nos1ap* knockout mice and characterization by ECG and echocardiography (Sugiyama *et al.* 2016). However, there were no data showing loss of *Nos1ap* transcript and/or protein in mutant mice, which we provide both for the constitutive and tissue-restricted null. The targeted mouse ES cells used in that study carried deletion of *Nos1ap* exon 3, which, based on sequence alone, is expected to generate an in-frame 30 amino acid deletion (full-length wild-type protein 503 amino acids) that likely has limited impact on protein structure and function. In contrast, the KOMP generated mouse ES cells (Skarnes *et al.* 2011) we used targeted *Nos1ap* exon 4, deletion of which creates a frameshift, introducing a premature termination codon leading to nonsense-mediated mRNA decay and complete loss of protein. Moreover, the exon targeted in KOMP ES cells is a “critical” exon common to all transcript variants that, when deleted, creates a frameshift mutation (Skarnes *et al.* 2011). Also, in contrast to our findings, the *Nos1ap* mutant homozygotes in the C57BL/6J background were reported to be viable. At baseline, no difference between wild-type and *Nos1ap* knockout mice in surface ECG and echocardiography was reported. Lastly, following injection of doxorubicin, a drug known to induce cardiotoxicity (Zhang *et al.* 2012), the authors did report longer QTc intervals in *Nos1ap* knockout mice. Keeping in mind that no data showing loss of *Nos1ap* expression were reported, that conclusion remains debatable as similar changes in QTc intervals and several other physiological measurements were observed in doxorubicin-treated wild-type controls, indicating presence of mostly genotype-independent drug-induced effects (Sugiyama *et al.* 2016).

Since Cre expression in αMHC-MerCreMer transgene is driven by a cardiomyocyte-specific promoter (Sohal *et al.* 2001), the floxed allele in cardiac endothelial cells, fibroblasts, and other stromal cells in *Nos1ap*^fl/fl^; αMHC-MerCreMer mice will remain unexcised. Given the widespread expression of *NOS1AP* in various human and mouse tissues (Kapoor *et al.* 2014; GTEx Consortium *et al.* 2017), it is likely that *Nos1ap* is also expressed in noncardiomyocyte cells in heart tissue. It is also possible that Cre-mediated excision of the floxed allele (2 copies per cell in *Nos1ap*^fl/fl^) is not complete in all cardiomyocyte nuclei. Alone or together, these scenarios can explain why the loss of *Nos1ap* transcript expression in left ventricle tissue in *Nos1ap*^fl/fl^ was half of that in wild-type mice. In order to assess *Nos1ap* expression at the cell type level in mouse left ventricles, we checked for *Nos1ap* expression in a published single-cell RNA-seq (scRNA-seq) data set from adult (P56) left ventricle tissue (Wang *et al.* 2020). However, across the ∼2,500 cells sequenced, including both cardiomyocyte- and noncardiomyocyte-enriched cells, *Nos1ap* expression was not detected in any of the identified cell clusters, most likely due to limited sensitivity (median of 2,610 genes/cell) (Wang *et al.* 2020), in contrast to its detection by TaqMan Gene Expression assay in bulk tissue.

Although previous in vitro studies have implicated cardiomyocyte-based *Nos1ap* effect on cellular electrophysiology, it remains possible that other cell types are involved in the regulation of the cardiac electrical cycle. For example, a neuronal effect is possible given that the autonomic nervous system plays an important role in modulation of cardiac electrophysiology and arrhythmogenesis (Shen and Zipes 2014; Gordan *et al.* 2015) and that *Nos1ap* has the highest expression level in nervous system tissues (Jaffrey *et al.* 1998; Kapoor *et al.* 2014). Even when restricting to cardiomyocyte-mediated effects, incomplete deletion of *Nos1ap* floxed allele copies in cardiomyocytes of *Nos1ap*^fl/fl^; αMHC-MerCreMer mice may fail to produce sufficient loss of function necessary to induce an overt ECG phenotype. The converse is also possible, where a complete or near-complete *Nos1ap* loss of function (knockout) in cardiomyocytes is rescued by yet unknown compensatory pathways (Rossi *et al.* 2015; El-Brolosy and Stainier 2017). The latter raises an important question for the utility of gene knockouts to understand gene function. This is especially applicable for genes uncovered by GWAS of common diseases and traits, as is the case here, where an ideal in vivo model to assess variable gene expression–based outcomes should involve an allelic series with target gene expression varying from low to high, as opposed to its complete absence. Generating such an allelic series remains challenging, at least in higher vertebrate model organisms, although recent synthetic biological technologies for creating humanized mice provide a new avenue (Brosh *et al.* 2022).

## Supplementary Material

jkad208_Supplementary_Data

## Data Availability

All RNA-seq data are accessible at NCBI's GEO Series accession number GSE210266. Supplemental material available at G3 online.
