# Supplementary material for: Cardiac muscle–restricted partial loss of Nos1ap expression has limited but significant impact on electrocardiographic features
Source: G3 (Bethesda). 2023 Sep 14;13(11):jkad208. doi: 10.1093/g3journal/jkad208 (PMC10627271; doi:10.1093/g3journal/jkad208)

Genotype: Nos1ap$^{+/+}$; +/αMHC-MCM
Sex: Male
ECG: Conscious

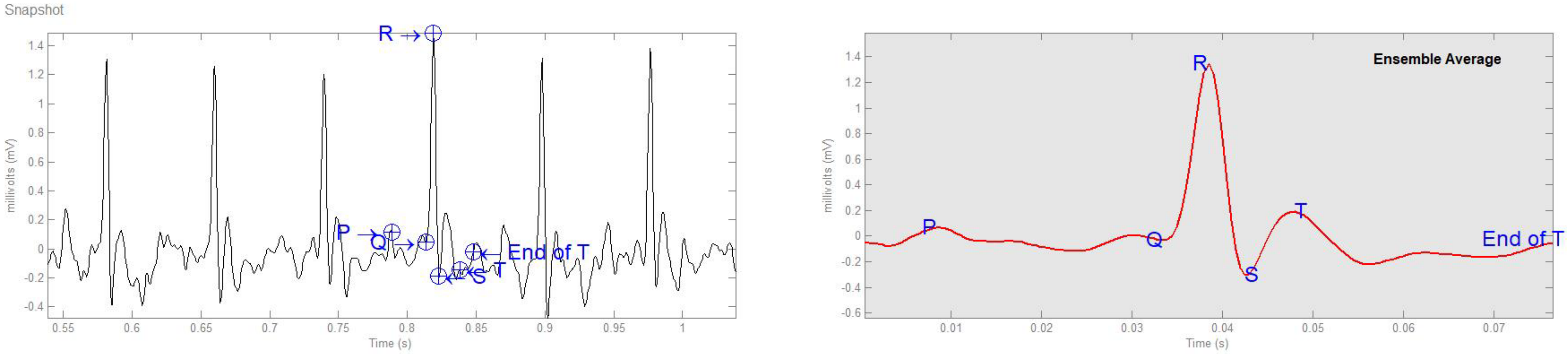

Genotype: Nos1ap$^{+/fl}$; +/αMHC-MCM
Sex: Male
ECG: Conscious

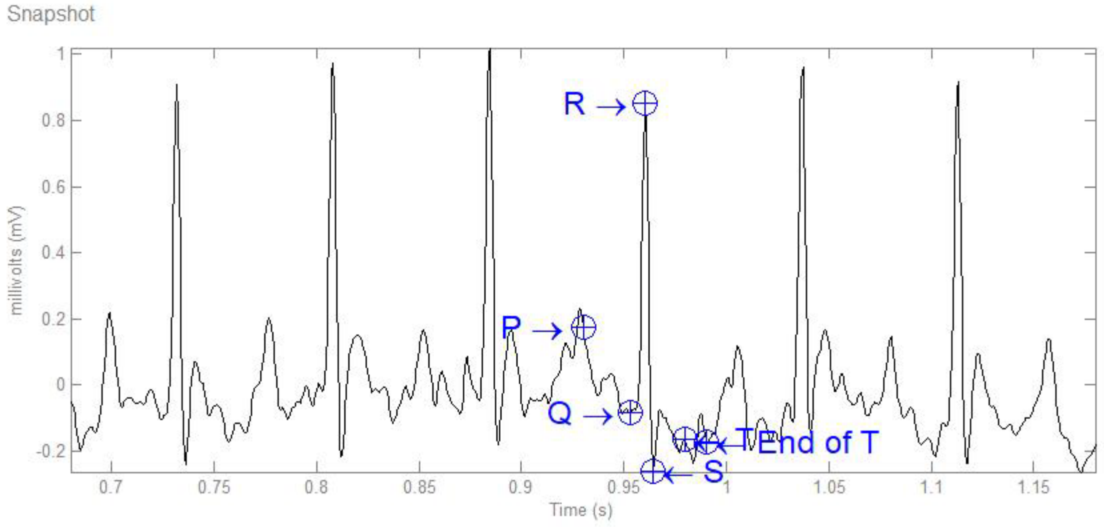

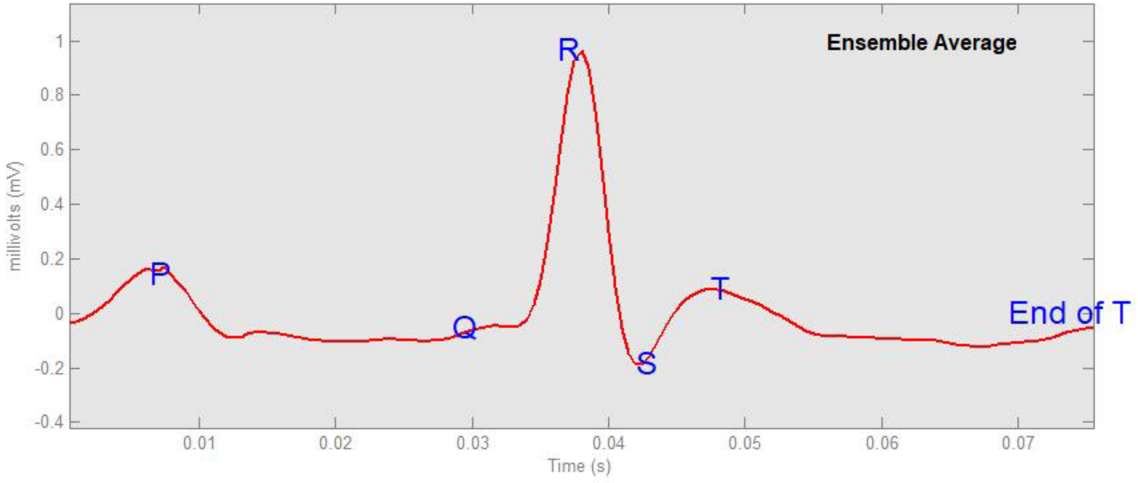

Genotype: Nos1ap$^{fl/fl}$; +/αMHC-MCM
Sex: Male
ECG: Conscious

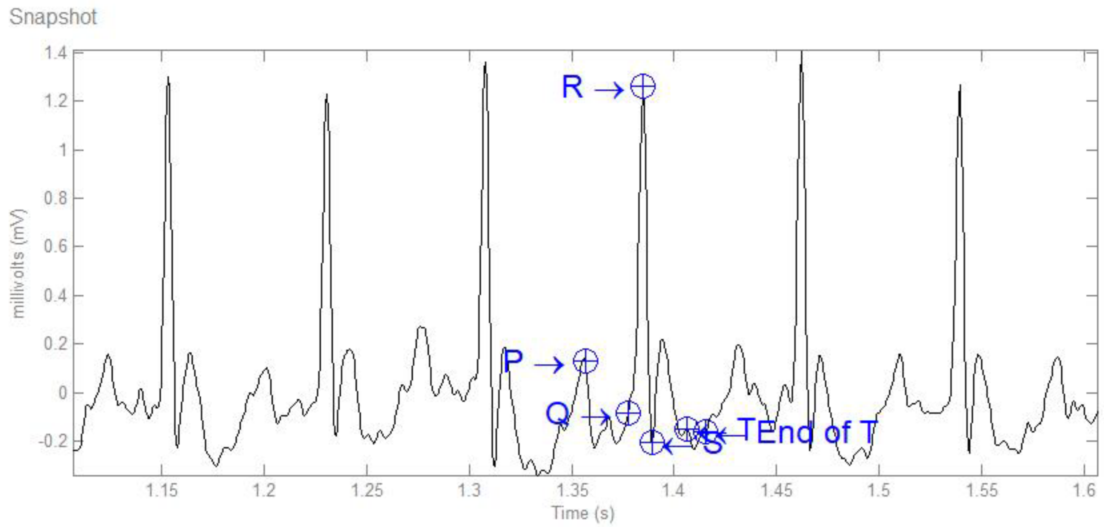

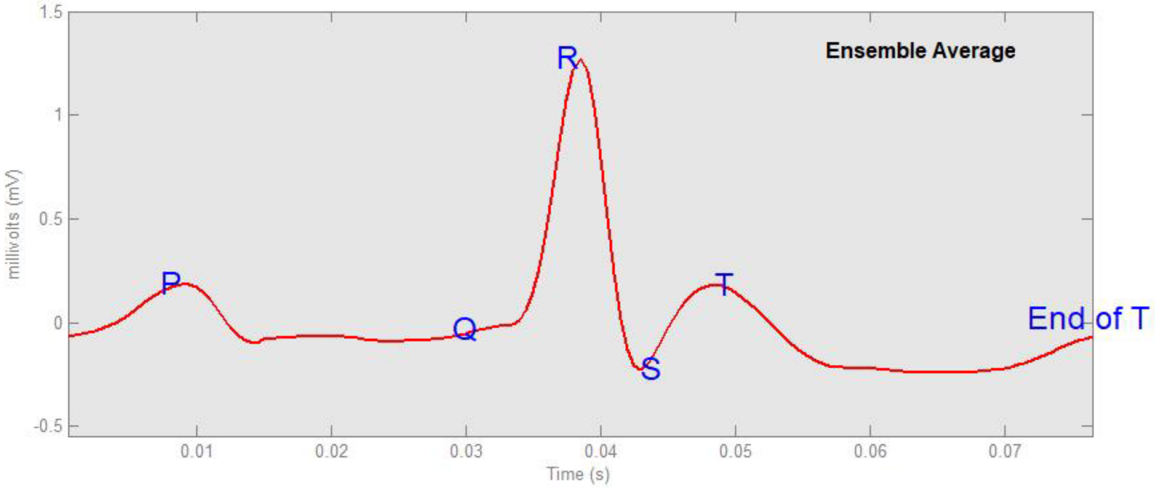

Genotype: Nos1ap$^{+/+}$; +/αMHC-MCM
Sex: Female
ECG: Conscious

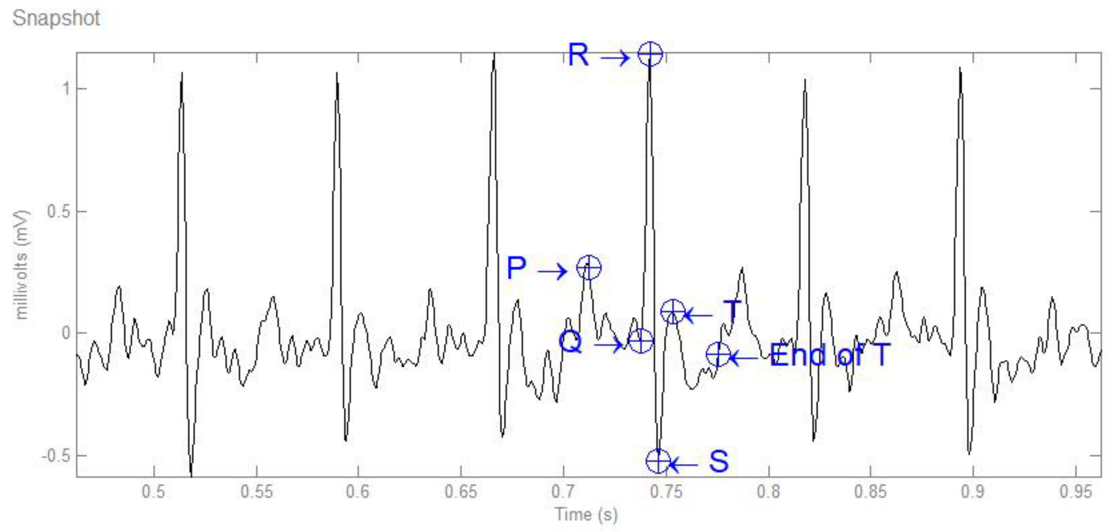

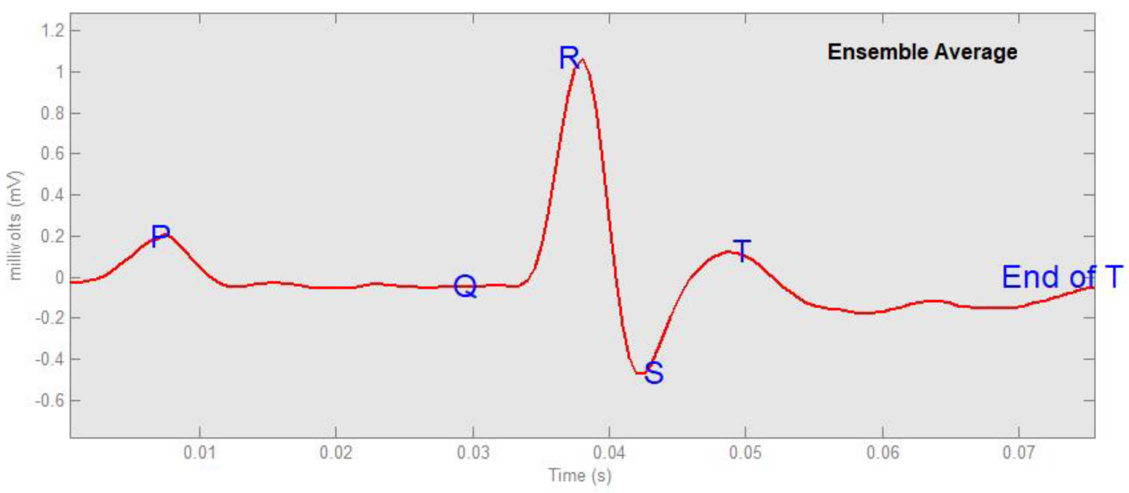

Genotype: Nos1ap$^{+/fl}$; +/αMHC-MCM
Sex: Female
ECG: Conscious

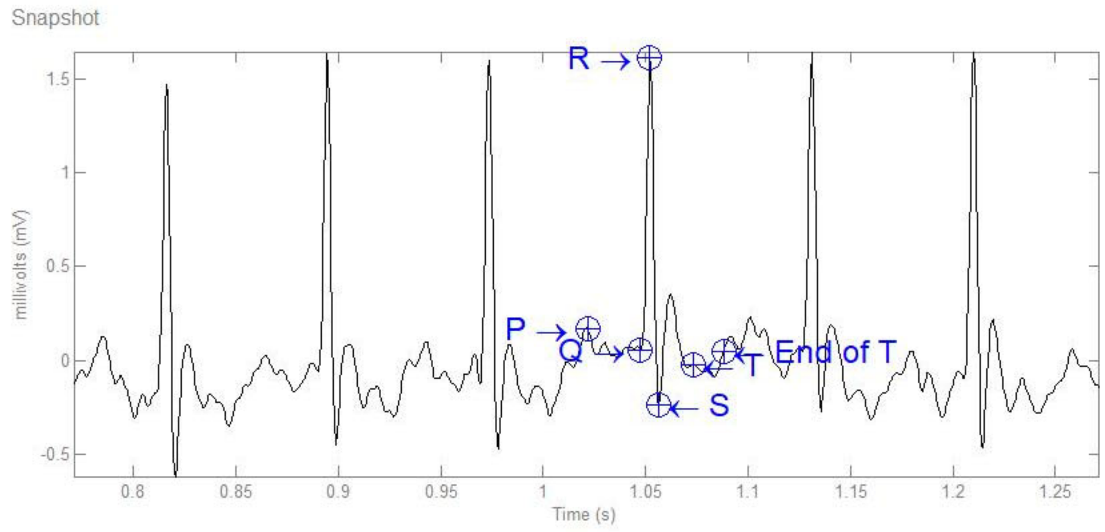

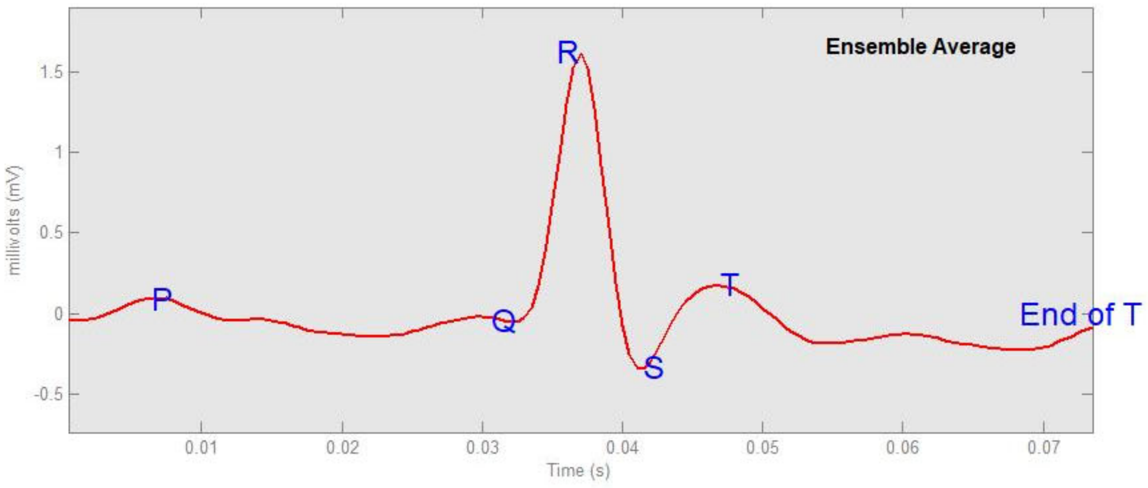

Genotype: Nos1ap$^{fl/fl}$; +/αMHC-MCM
Sex: Female
ECG: Conscious

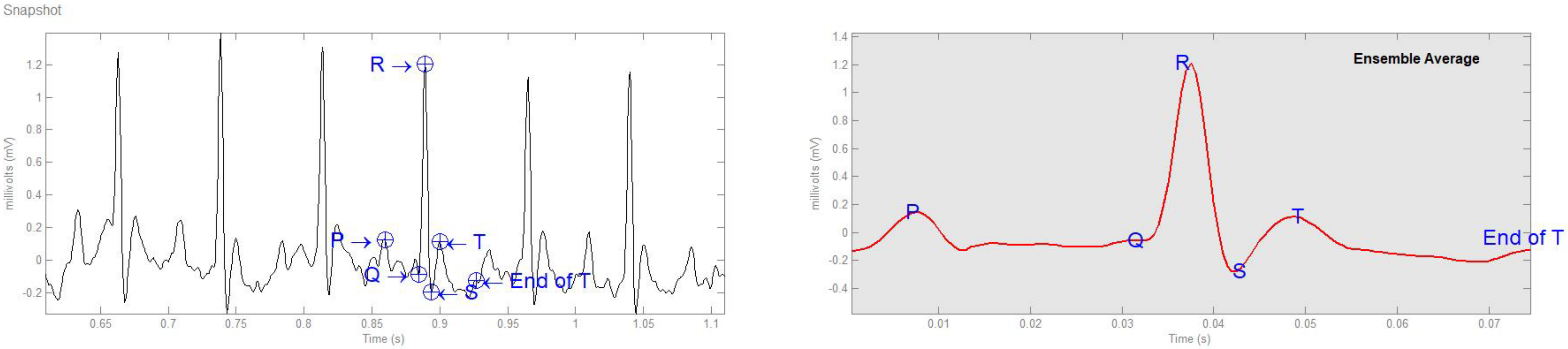

Genotype: Nos1ap$^{+/+}$; +/αMHC-MCM; Sex: Male; ECG: Anesthetized; Isoproterenol 1mg/kg

Baseline

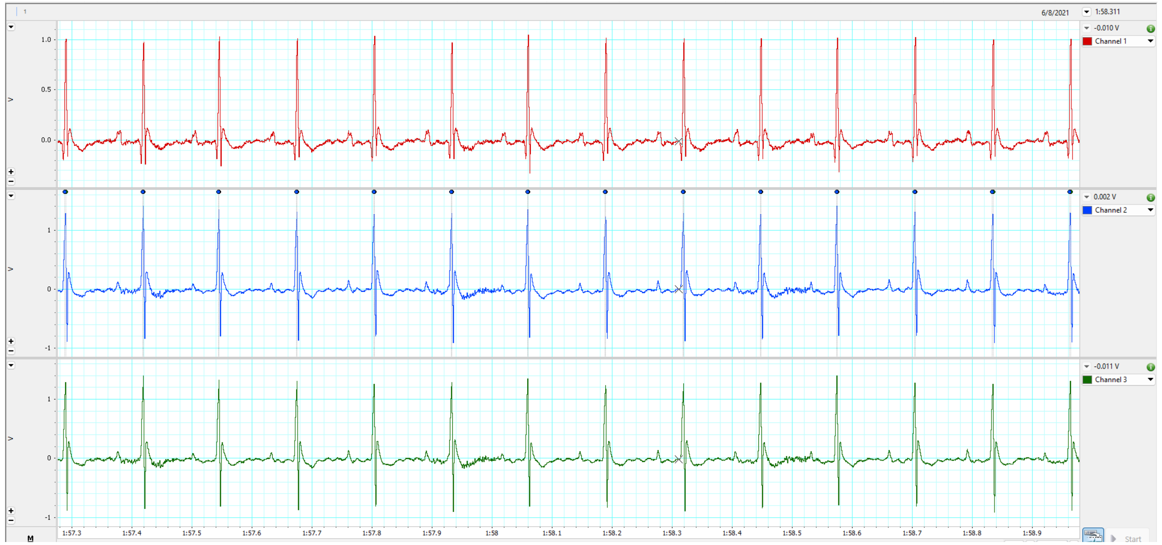

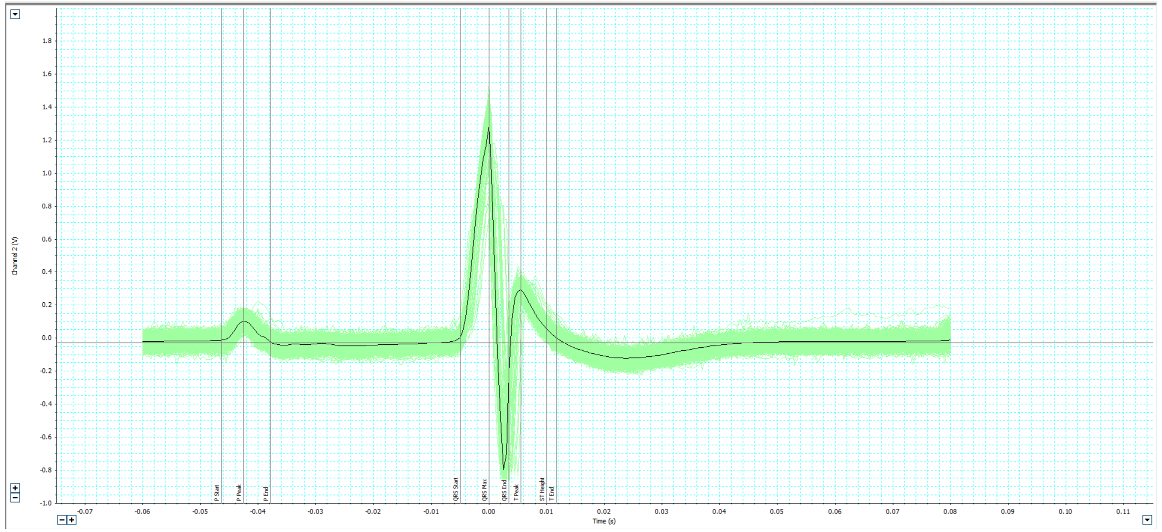

Drug

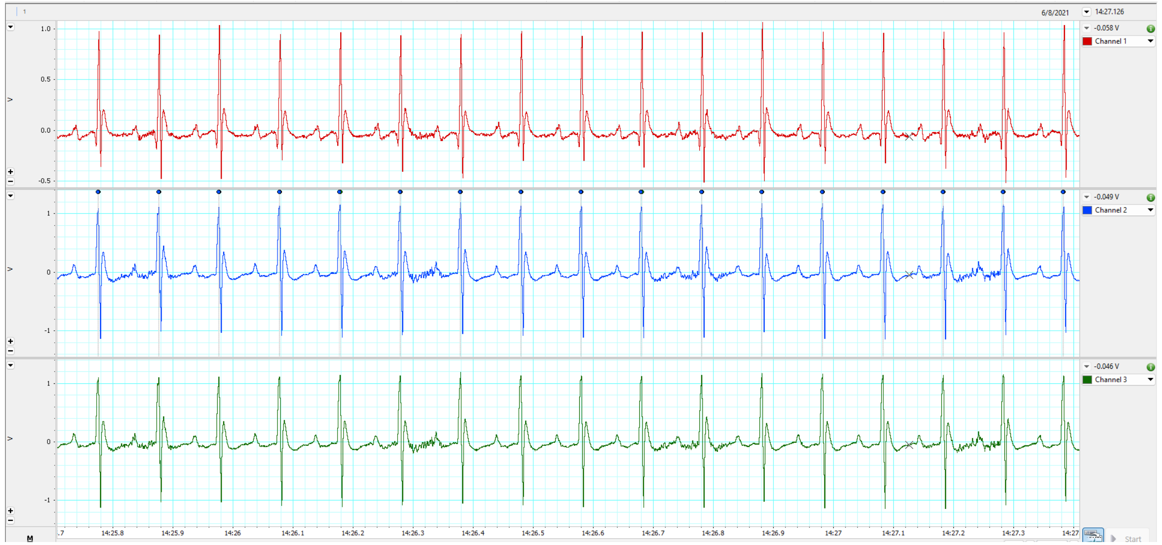

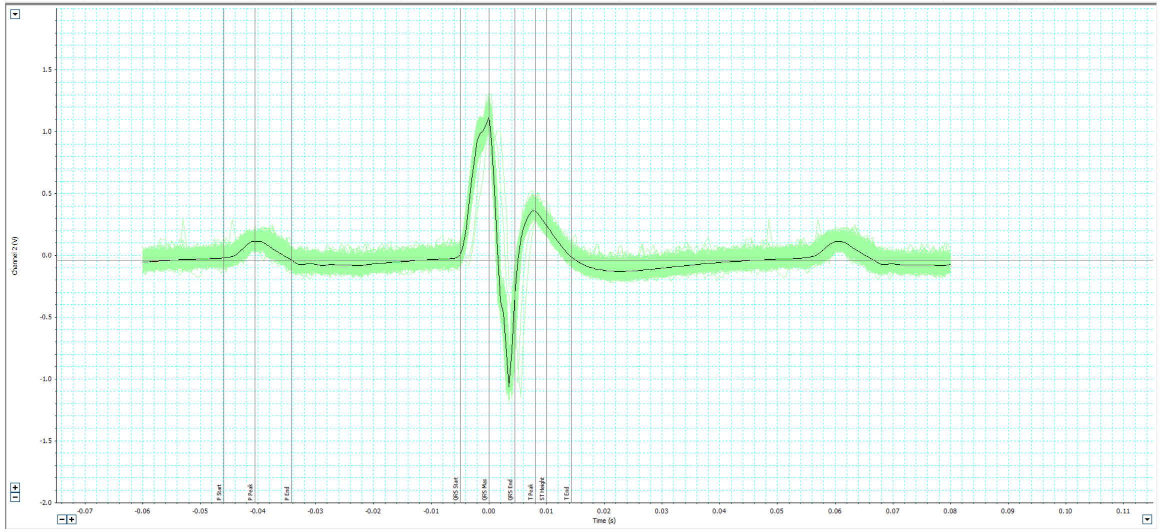

Genotype: Nos1ap$^{+/+}$; +/αMHC-MCM; Sex: Male; ECG: Anesthetized; Isoproterenol 5mg/kg

Baseline

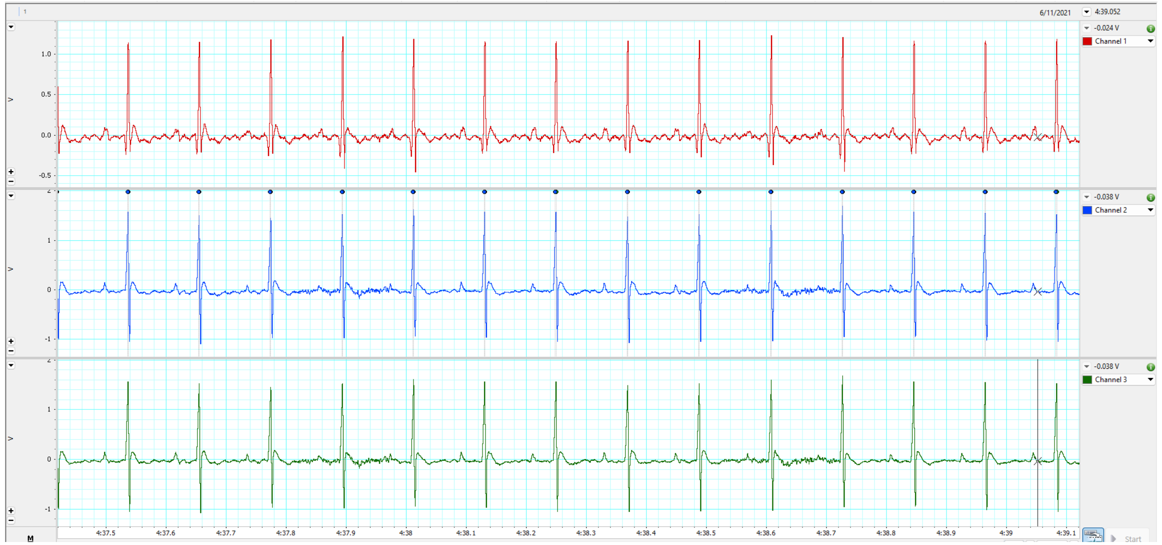

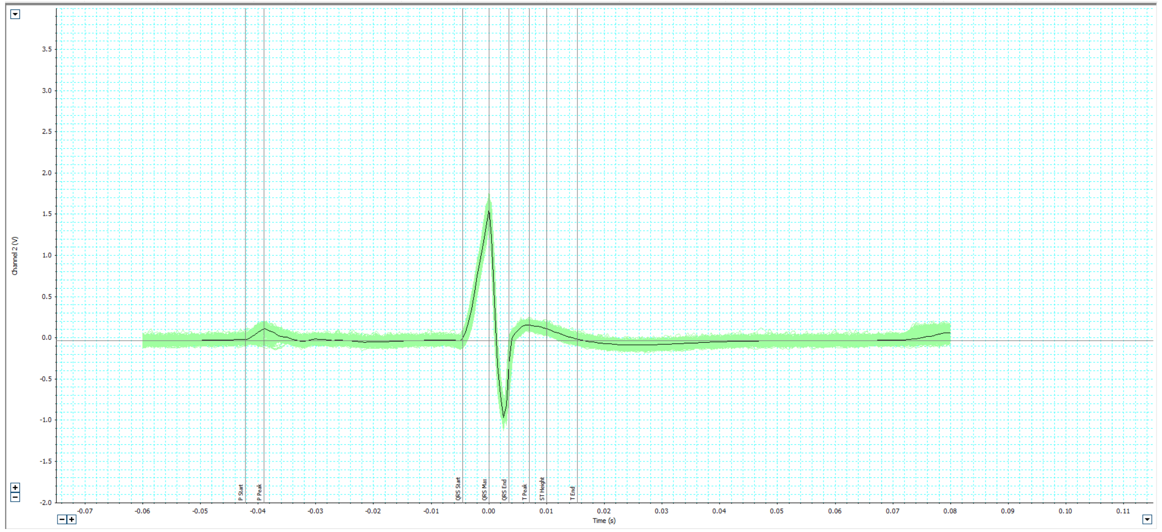

Drug

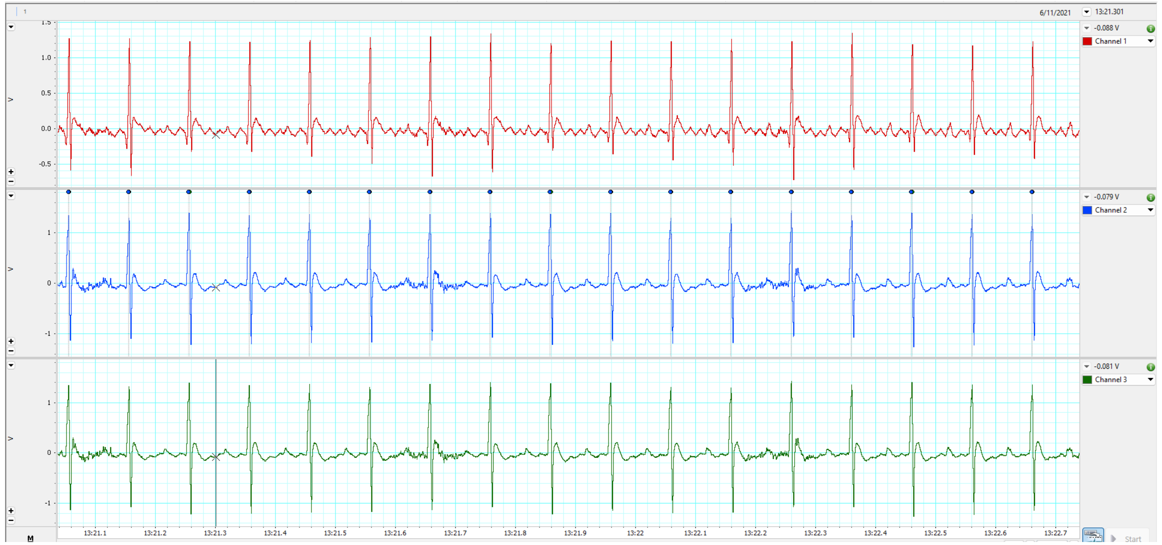

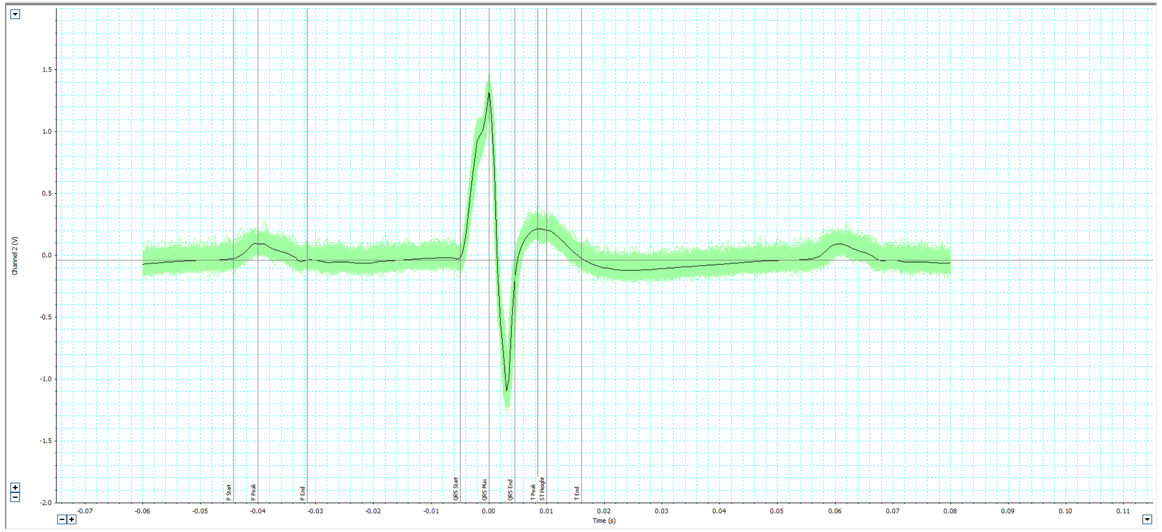

Genotype: Nos1ap$^{+/fl}$; +/αMHC-MCM; Sex: Male; ECG: Anesthetized; Isoproterenol 1mg/kg

Baseline

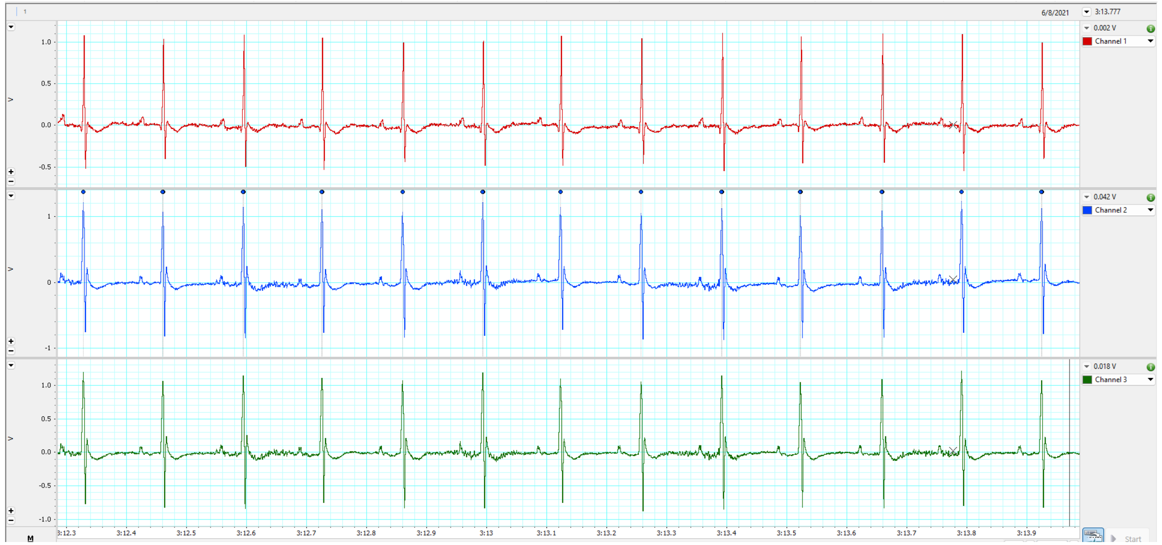

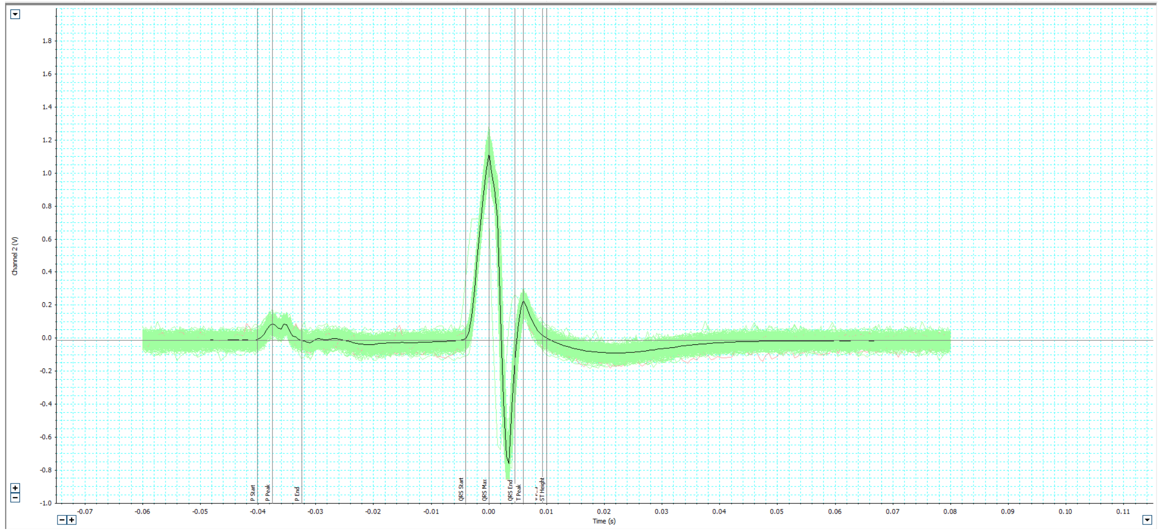

Drug

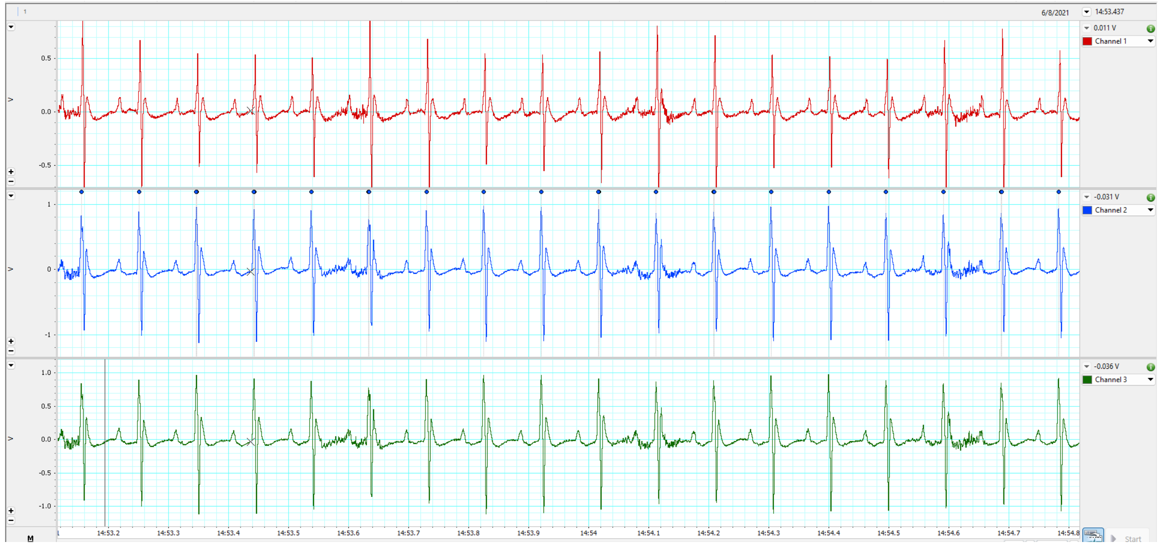

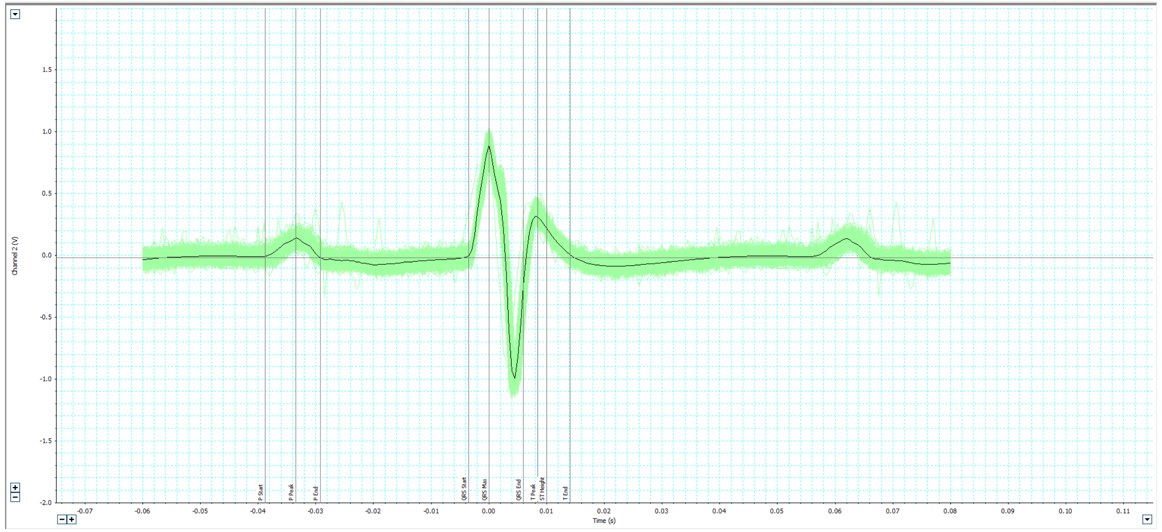

Genotype: Nos1ap$^{+/fl}$; +/αMHC-MCM; Sex: Male; ECG: Anesthetized; Isoproterenol 5mg/kg

Baseline

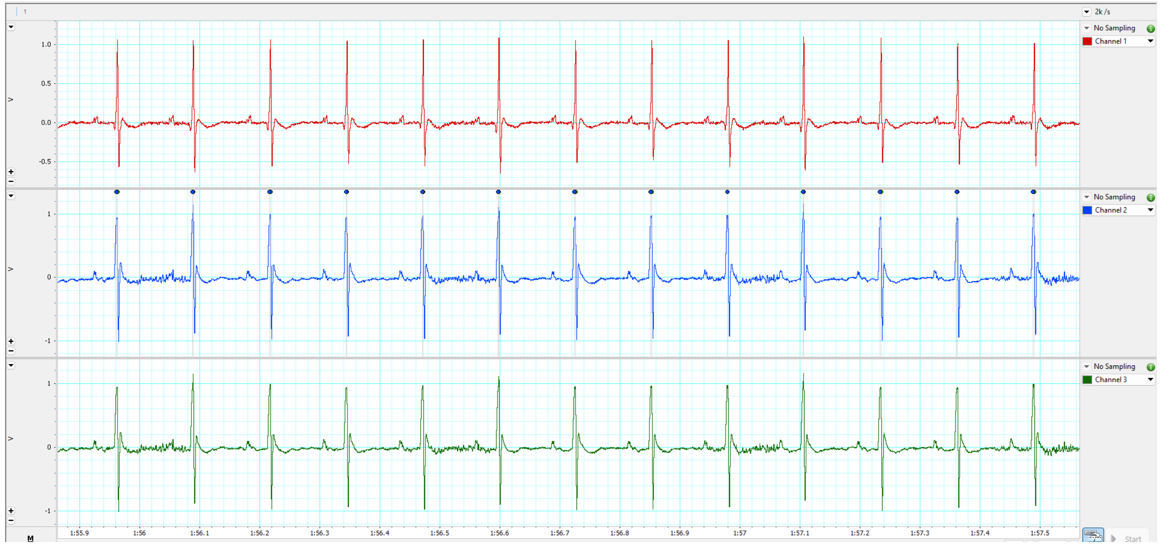

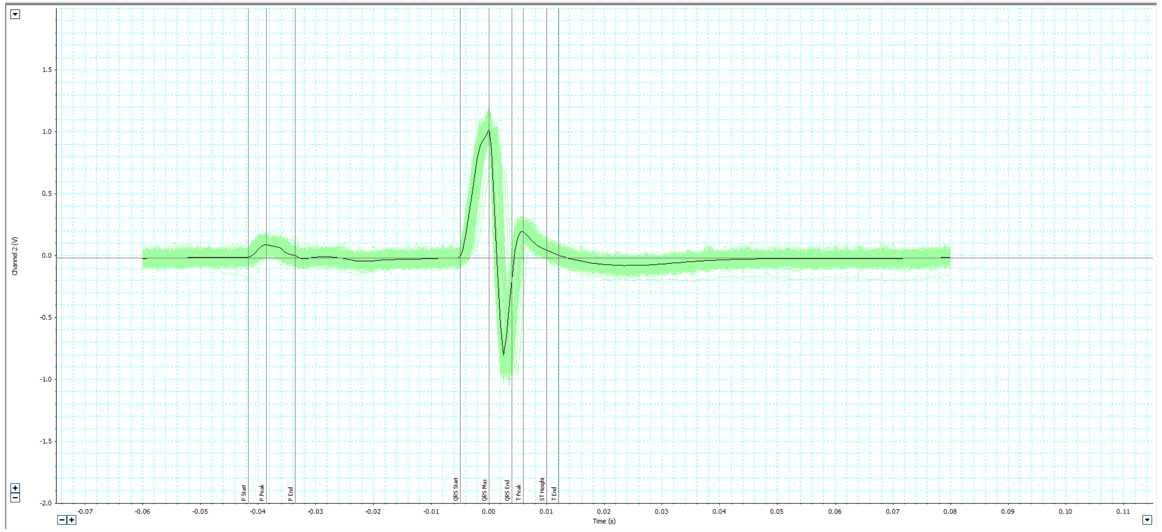

Drug

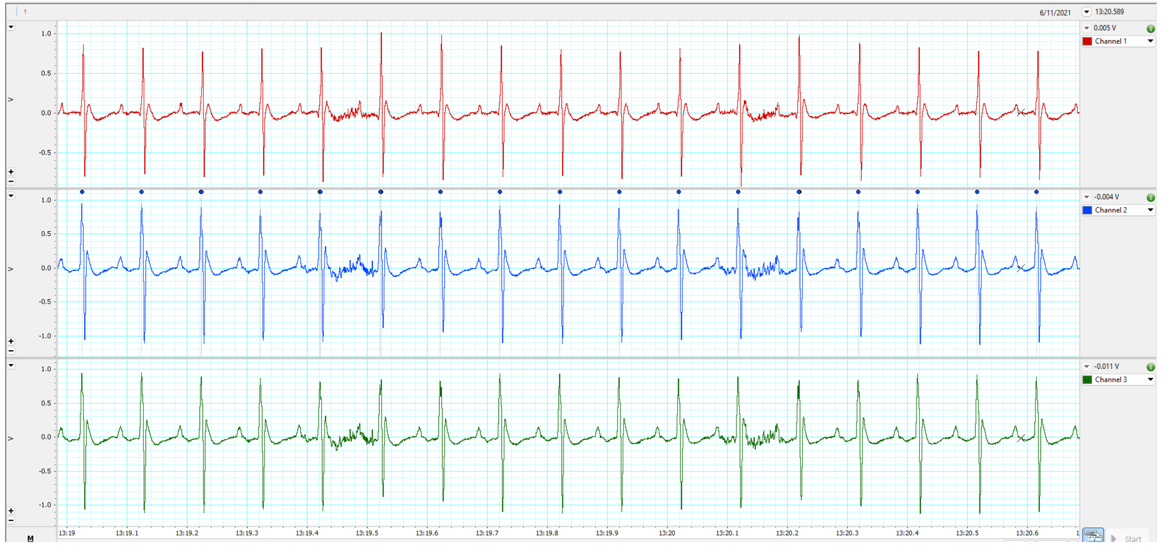

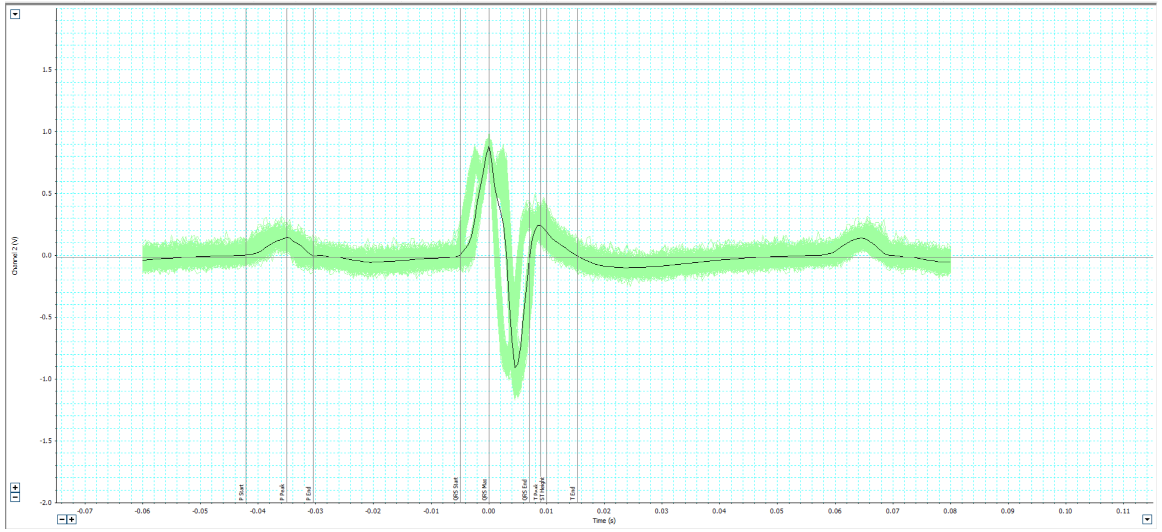

Genotype: Nos1ap[fl/fl]; +/αMHC-MCM; Sex: Male; ECG: Anesthetized; Isoproterenol 1mg/kg

Baseline

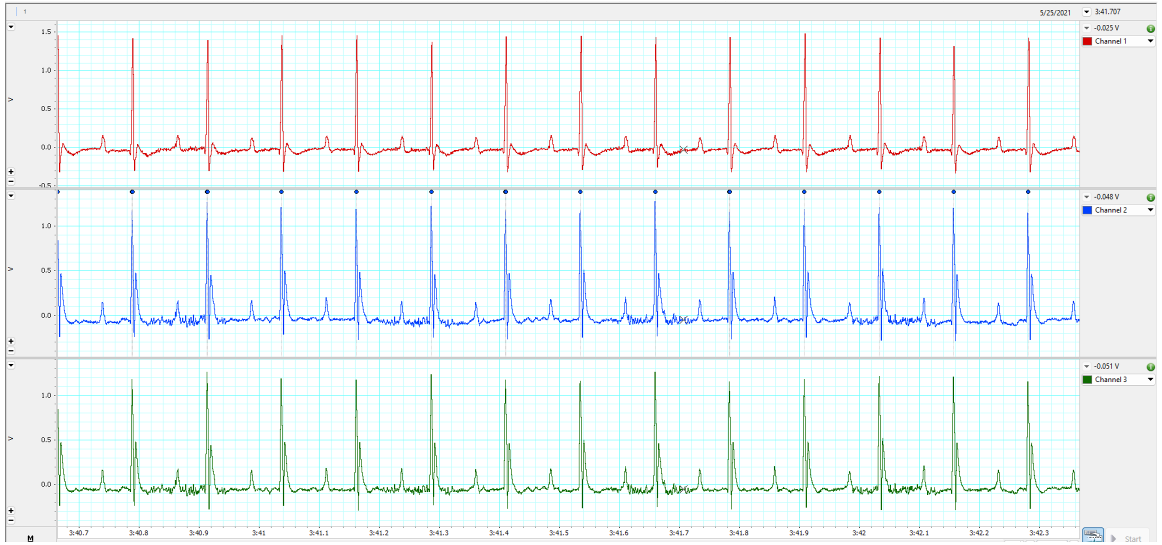

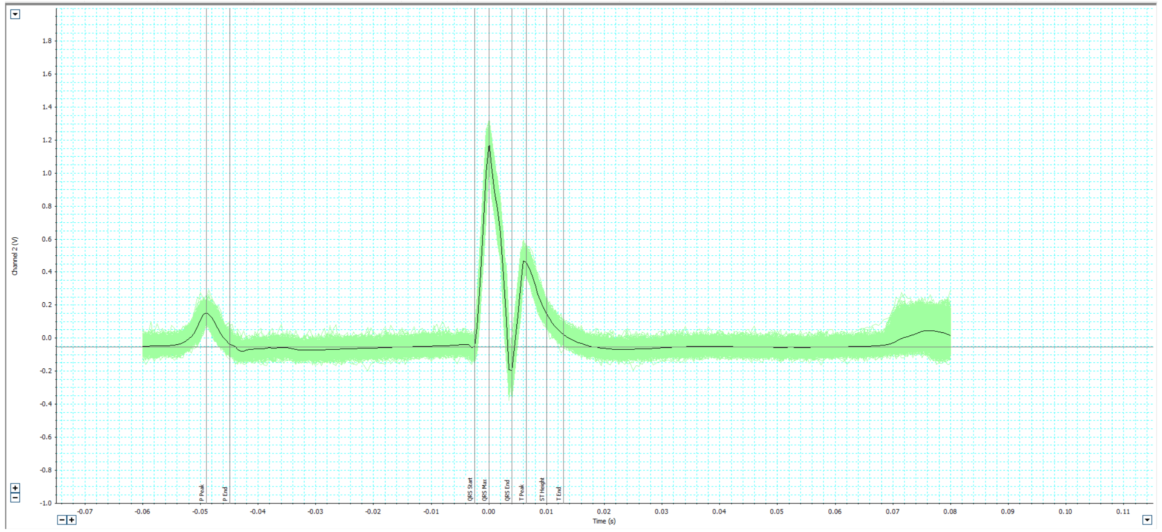

Drug

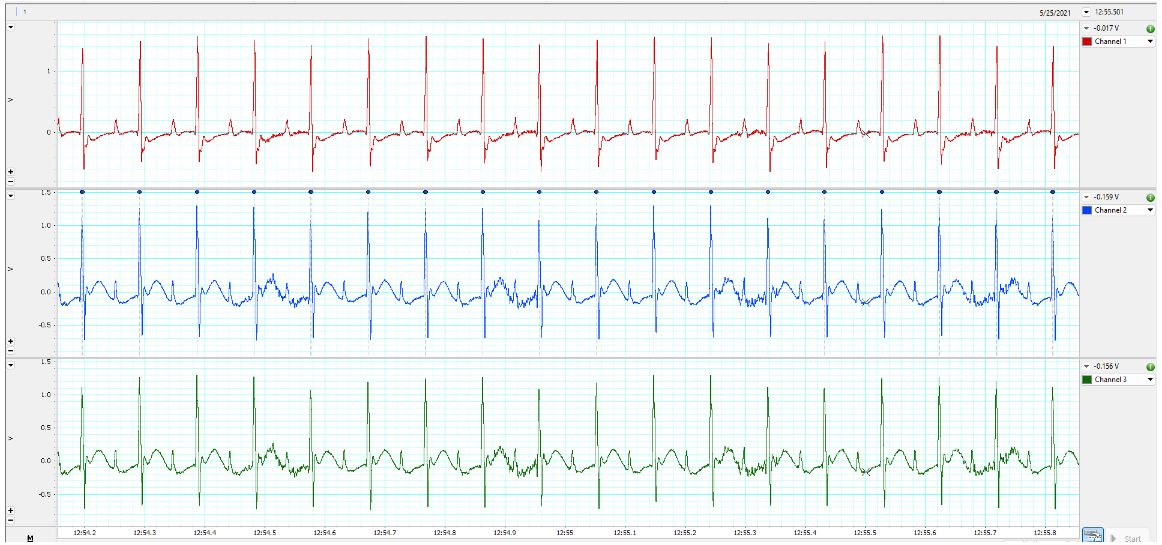

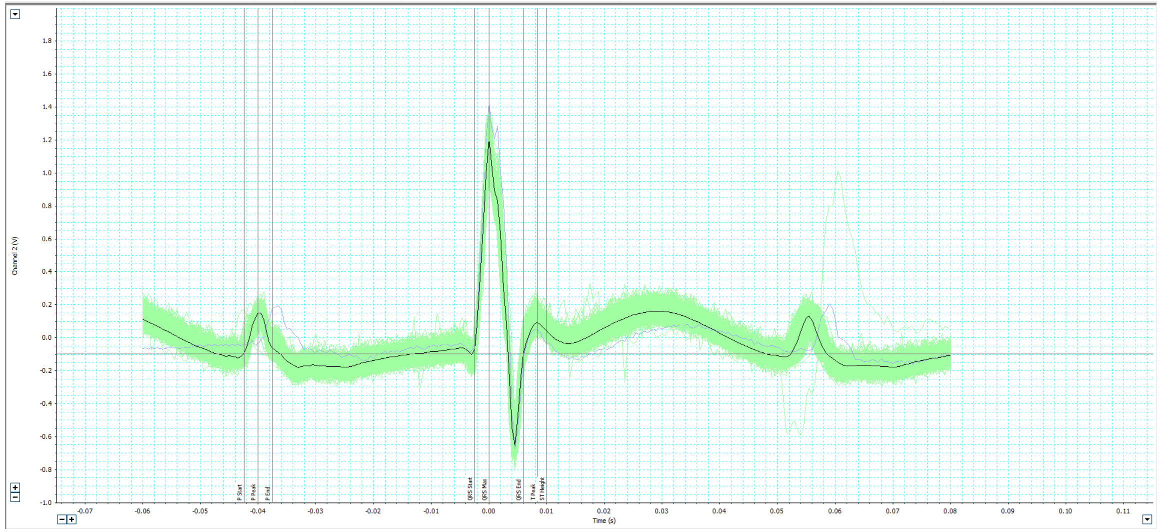

Genotype: Nos1ap$^{fl/fl}$; +/αMHC-MCM; Sex: Male; ECG: Anesthetized; Isoproterenol 5mg/kg

Baseline

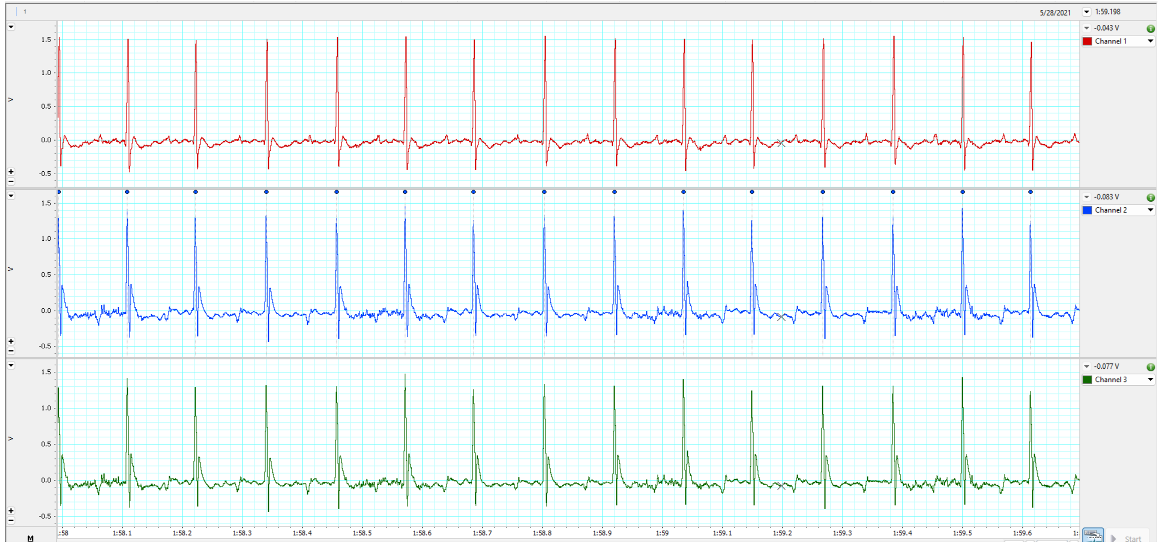

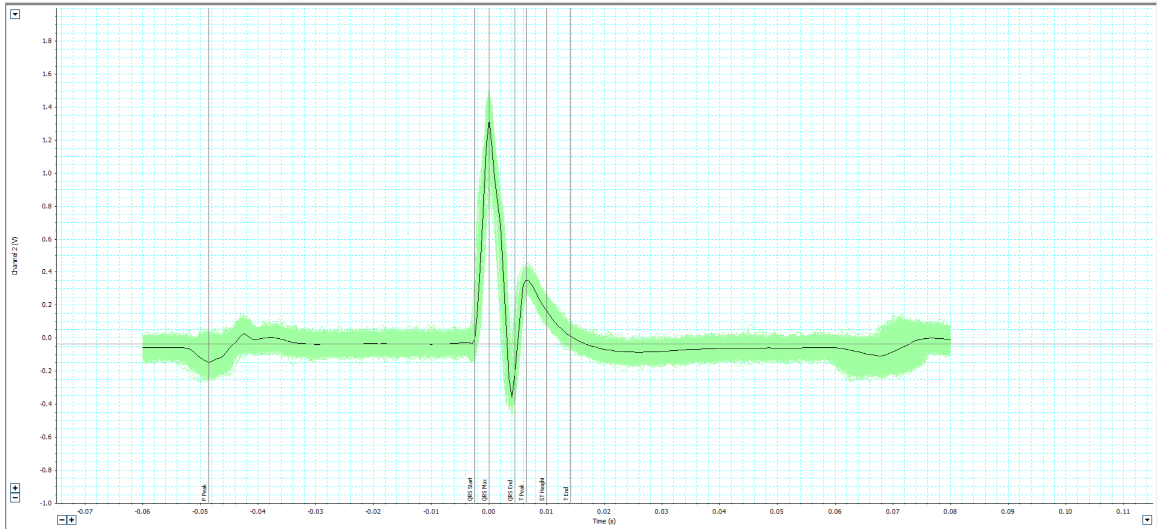

Drug

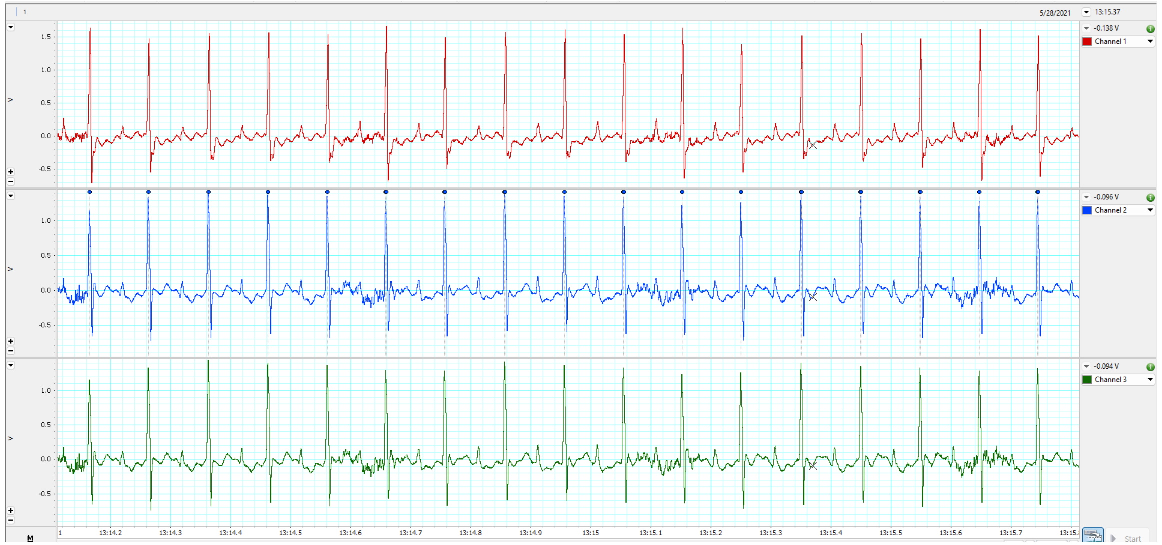

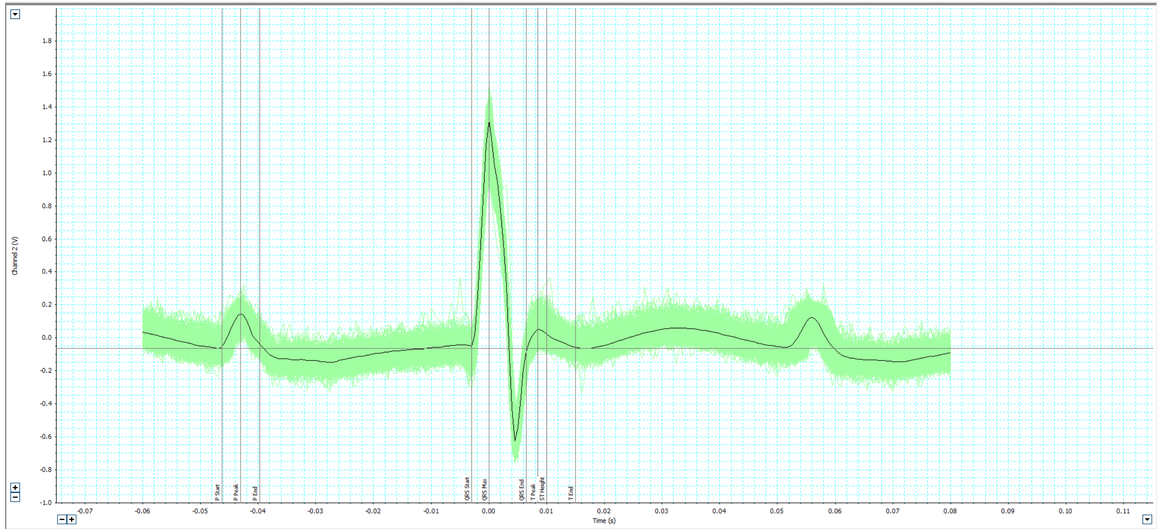

Genotype: Nos1ap$^{+/+}$; +/αMHC-MCM; Sex: Female; ECG: Anesthetized; Isoproterenol 1mg/kg

Baseline

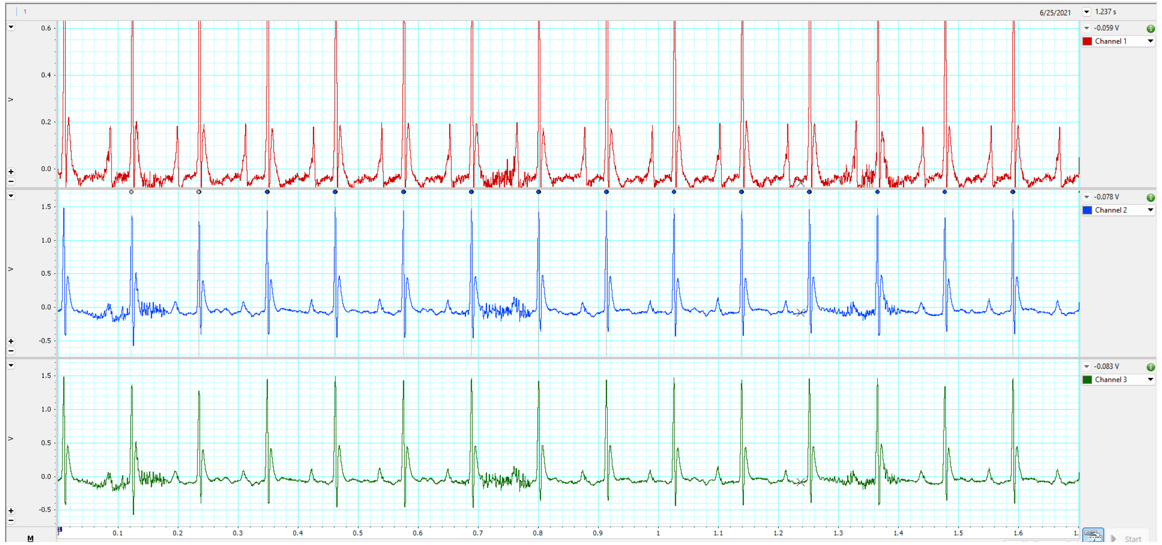

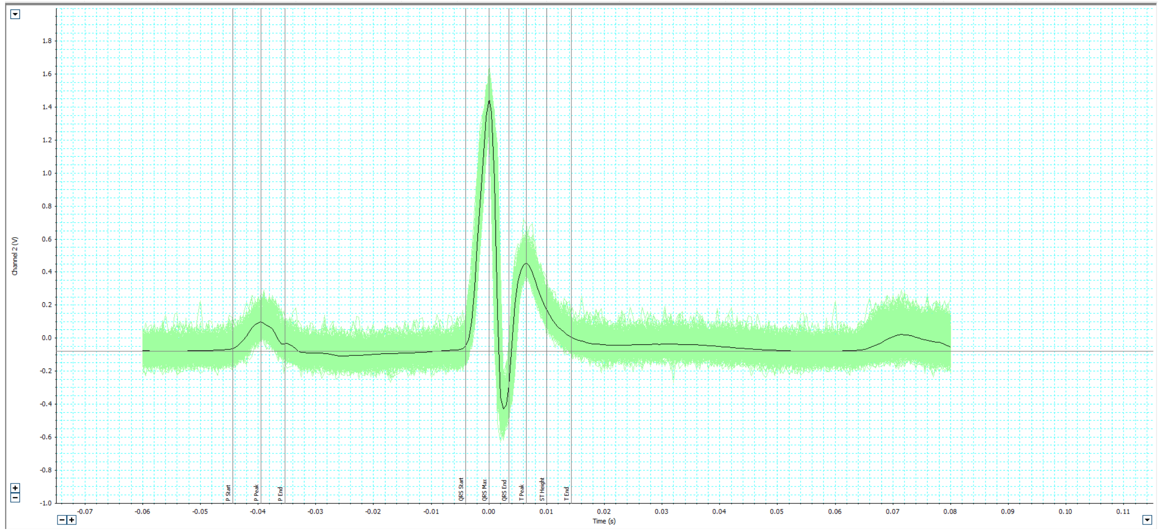

Drug

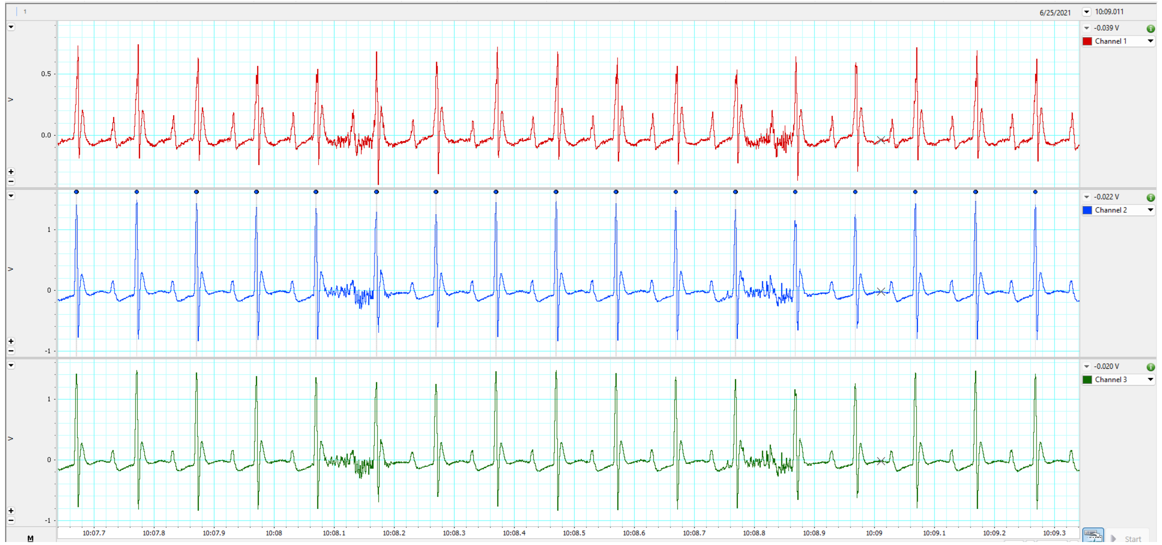

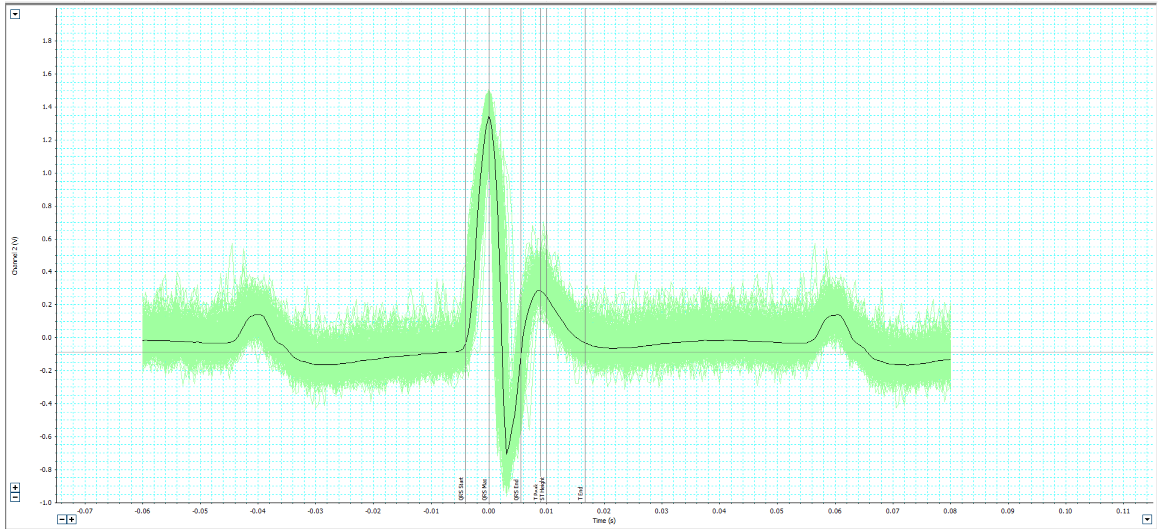

Genotype: Nos1ap$^{+/+}$; +/αMHC-MCM; Sex: Female; ECG: Anesthetized; Isoproterenol 5mg/kg

Baseline

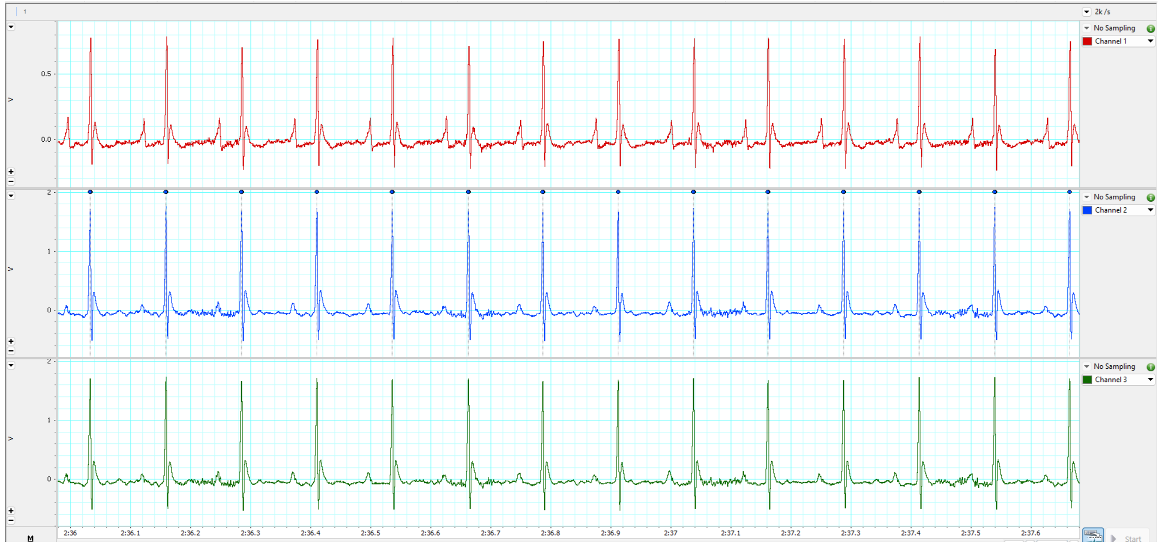

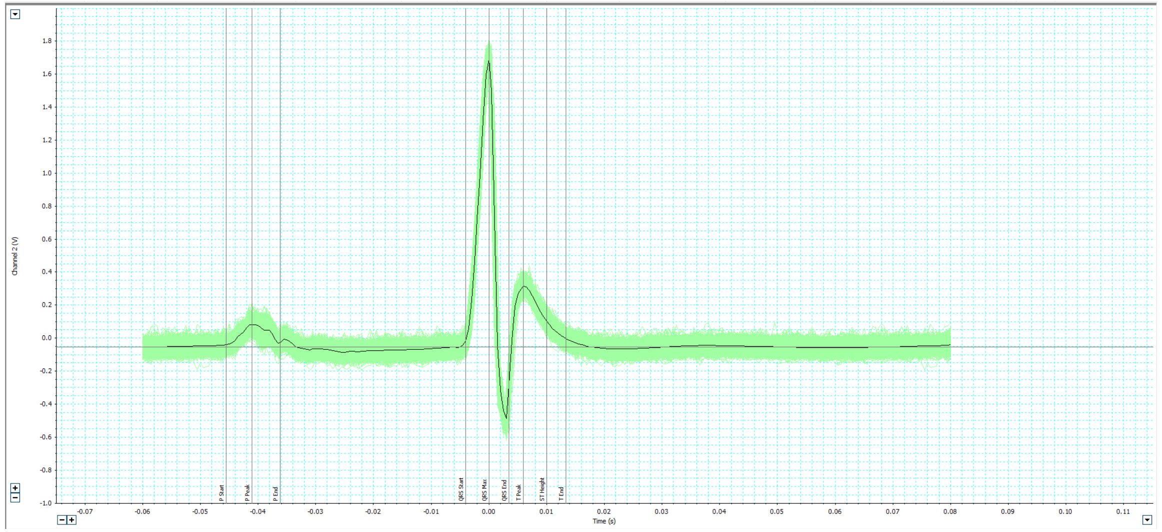

Drug

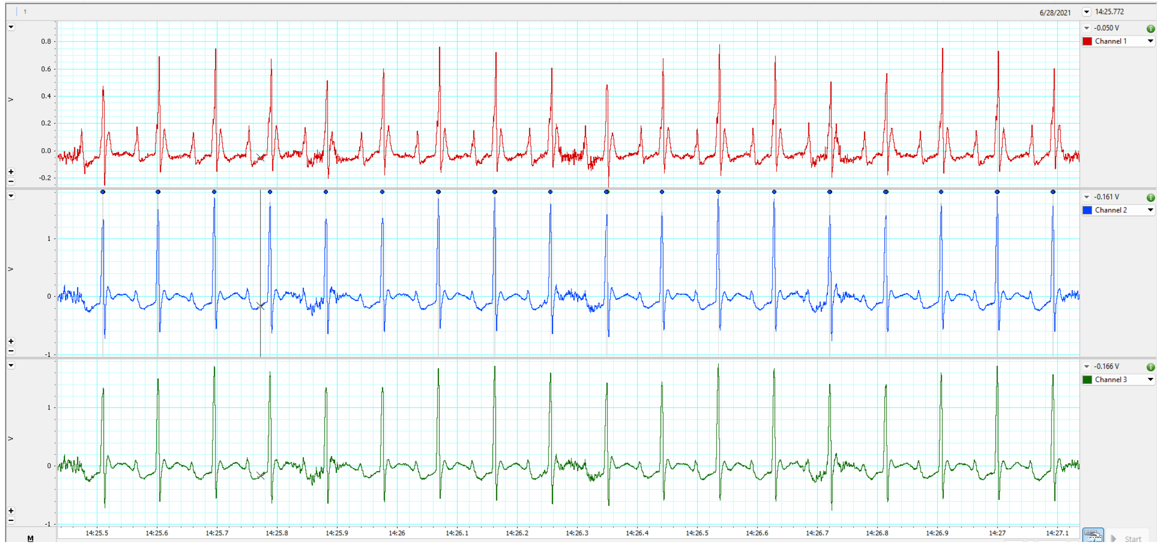

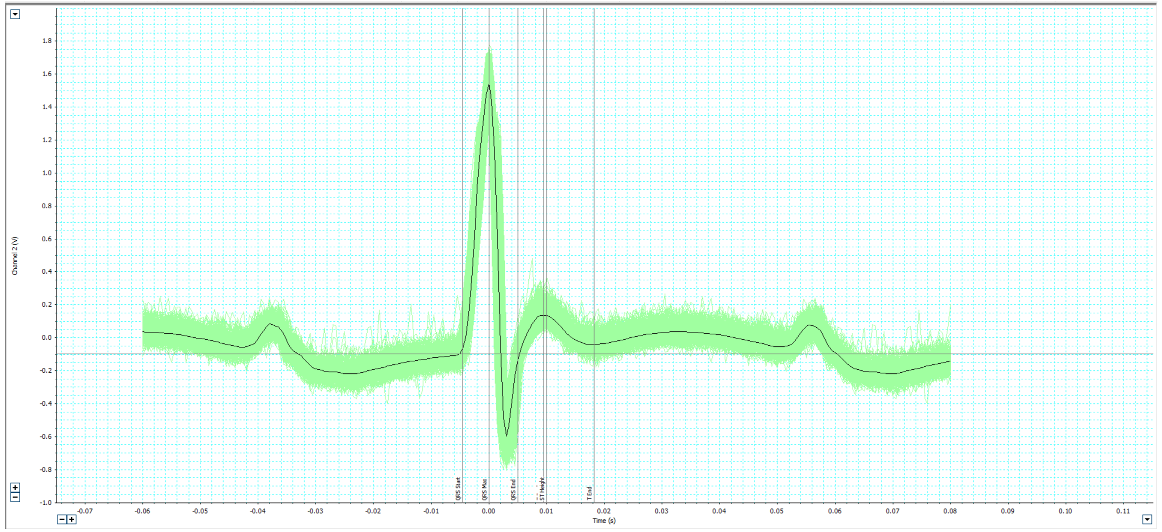

Genotype: Nos1ap$^{+/fl}$; +/αMHC-MCM; Sex: Female; ECG: Anesthetized; Isoproterenol 1mg/kg

Baseline

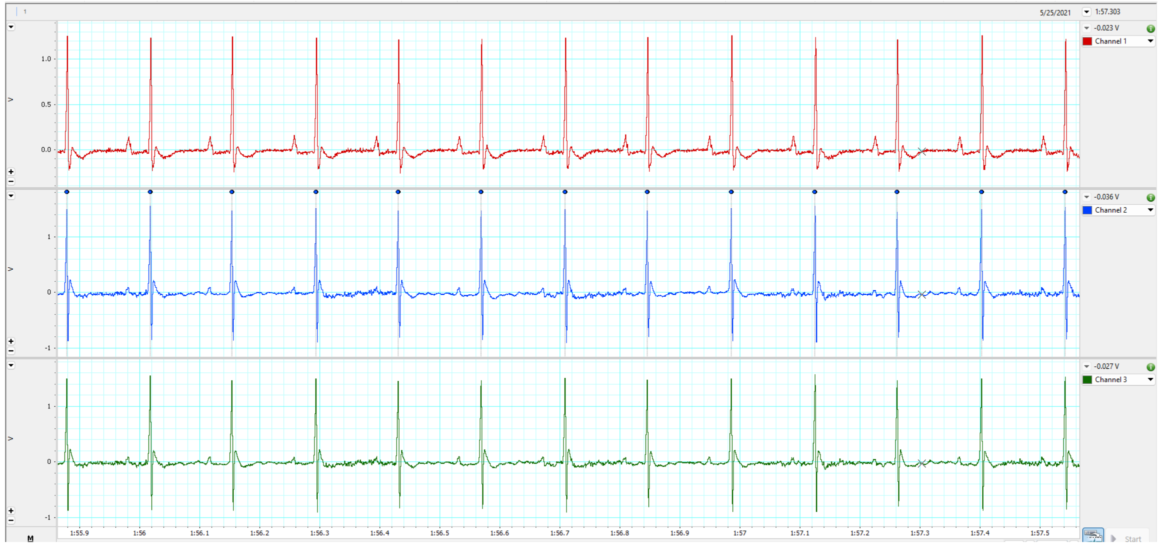

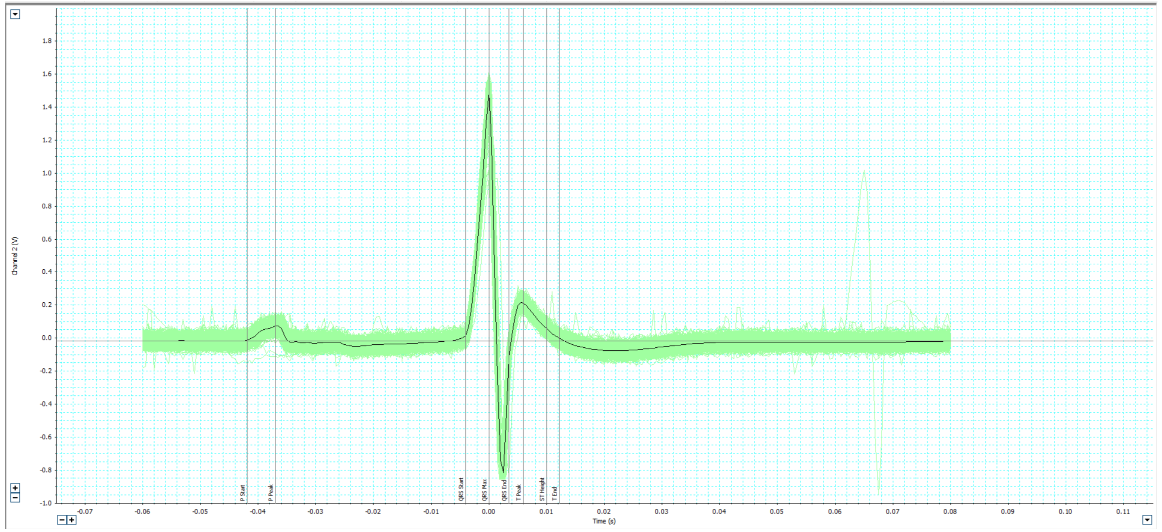

Drug

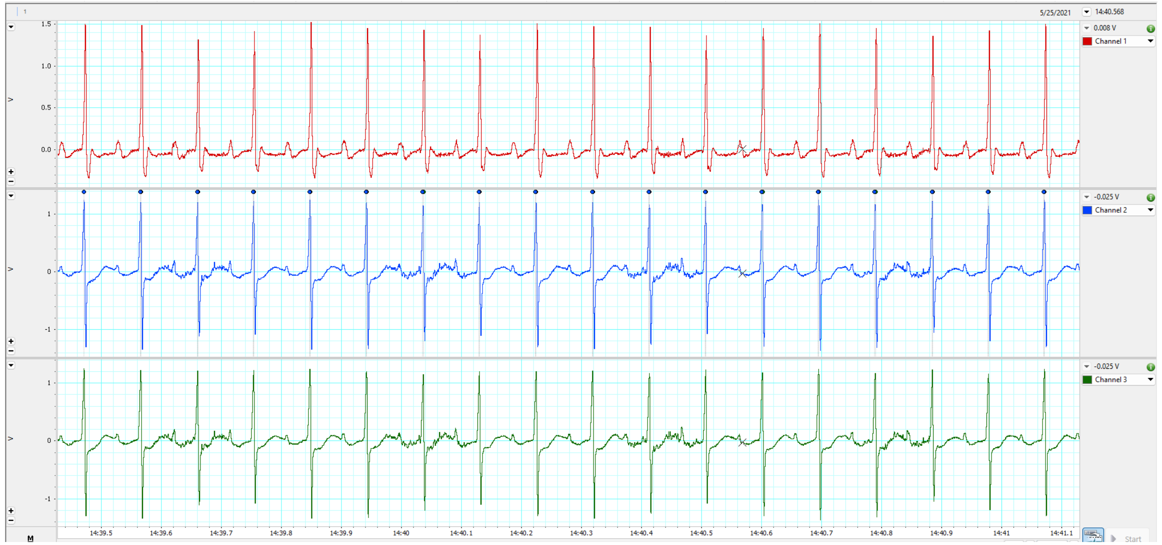

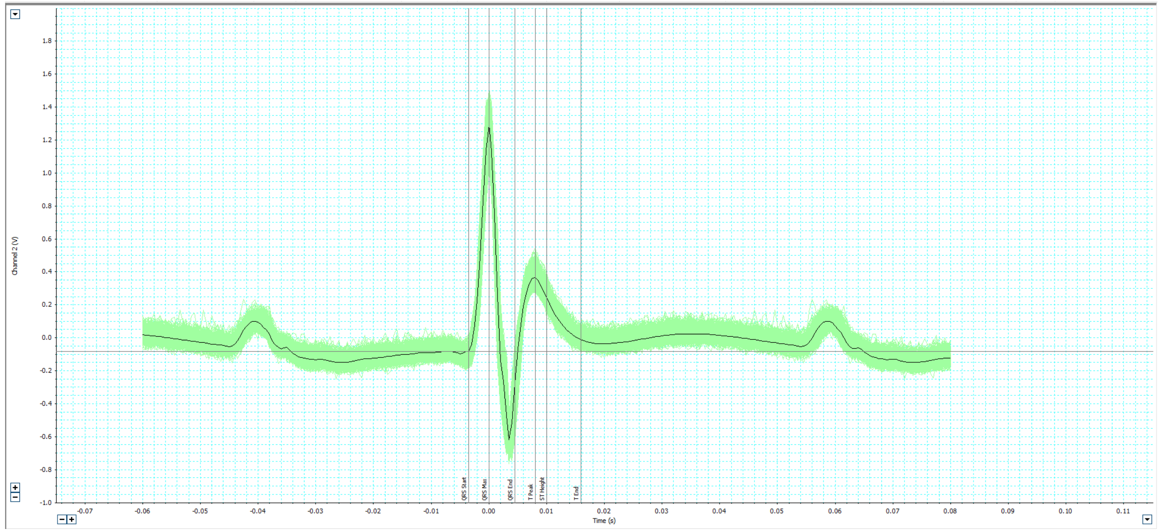

Genotype: Nos1ap$^{+/fl}$; +/αMHC-MCM; Sex: Female; ECG: Anesthetized; Isoproterenol 5mg/kg

Baseline

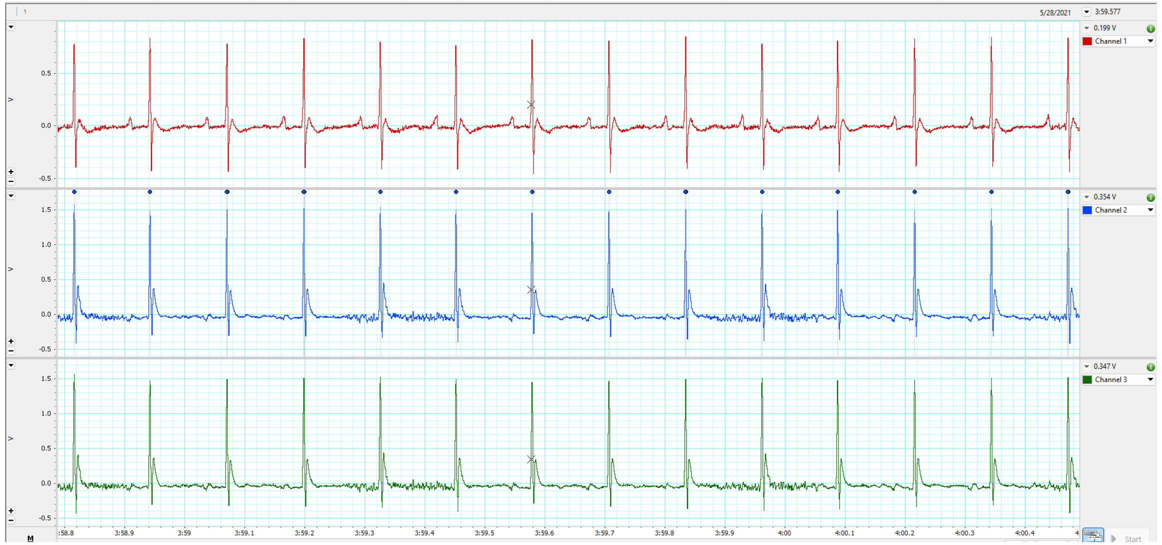

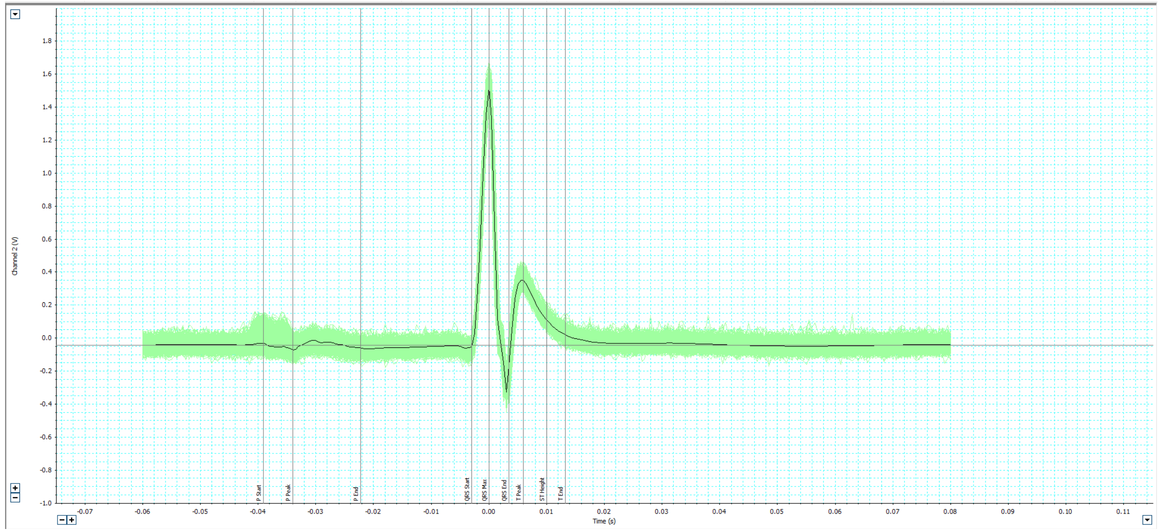

Drug

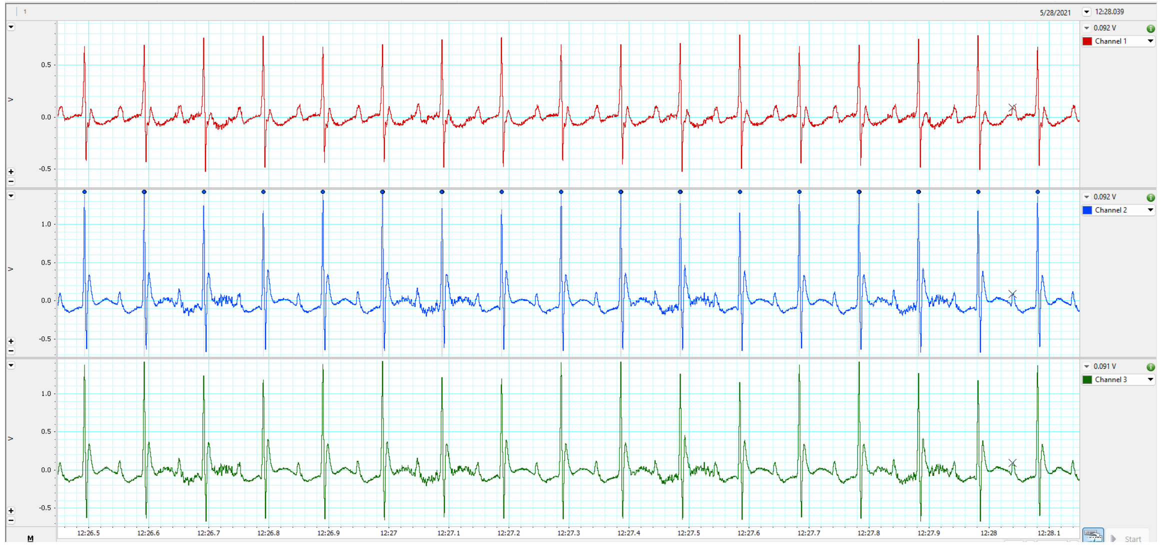

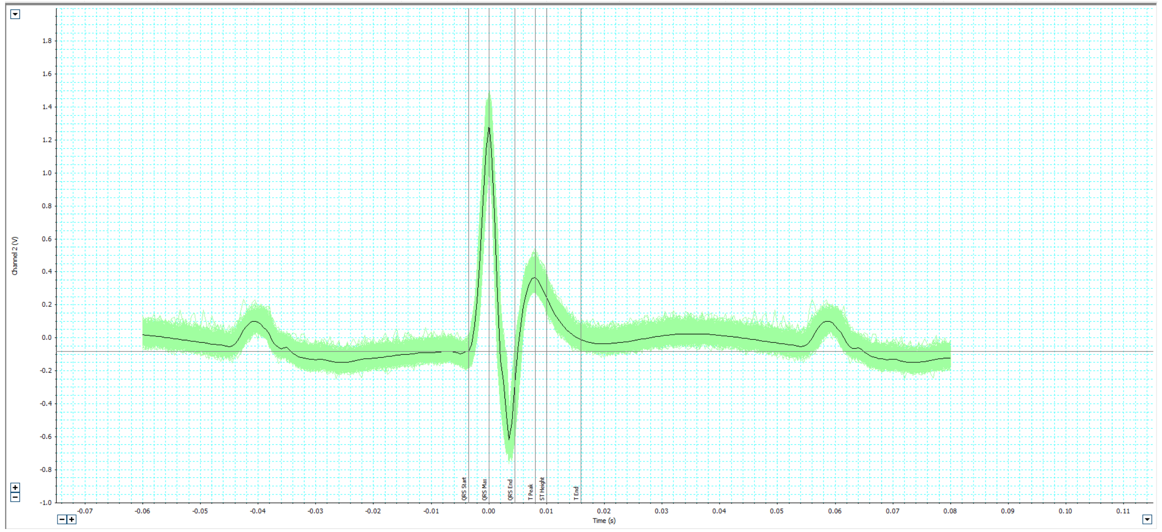

Genotype: Nos1ap$^{fl/fl}$; +/αMHC-MCM; Sex: Female; ECG: Anesthetized; Isoproterenol 1mg/kg

Baseline

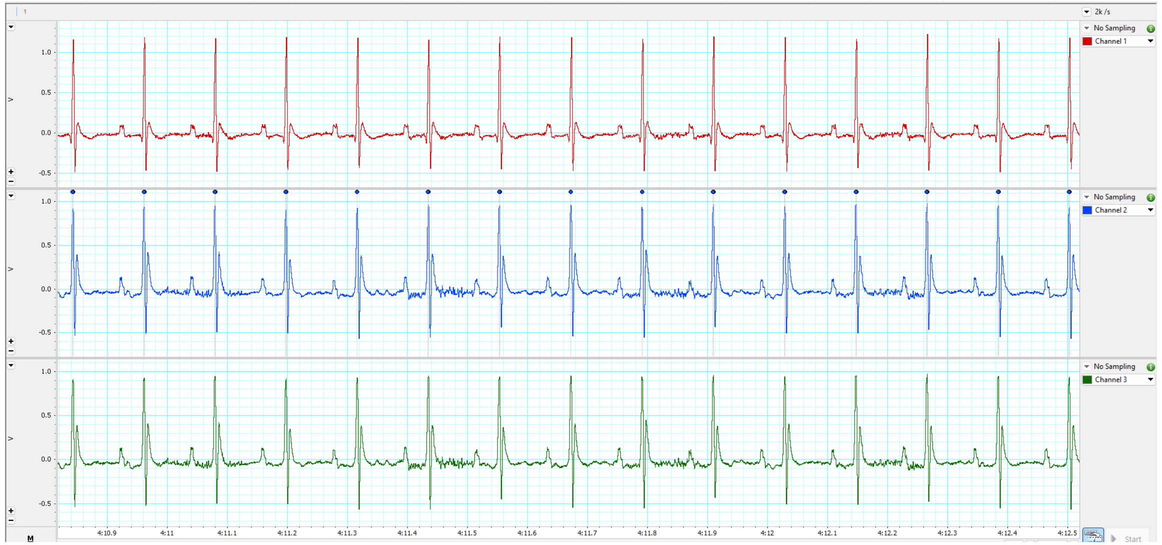

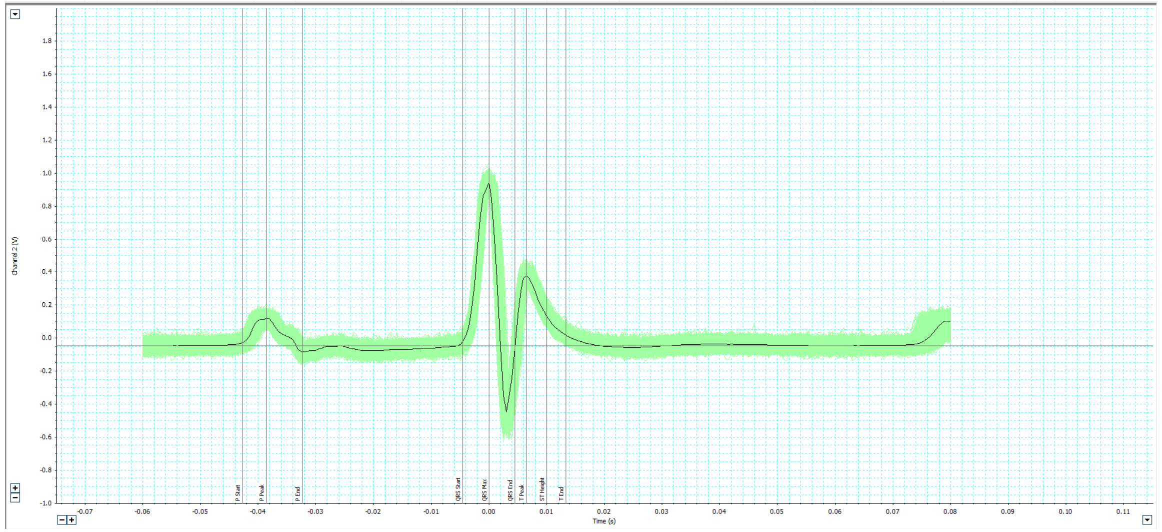

Drug

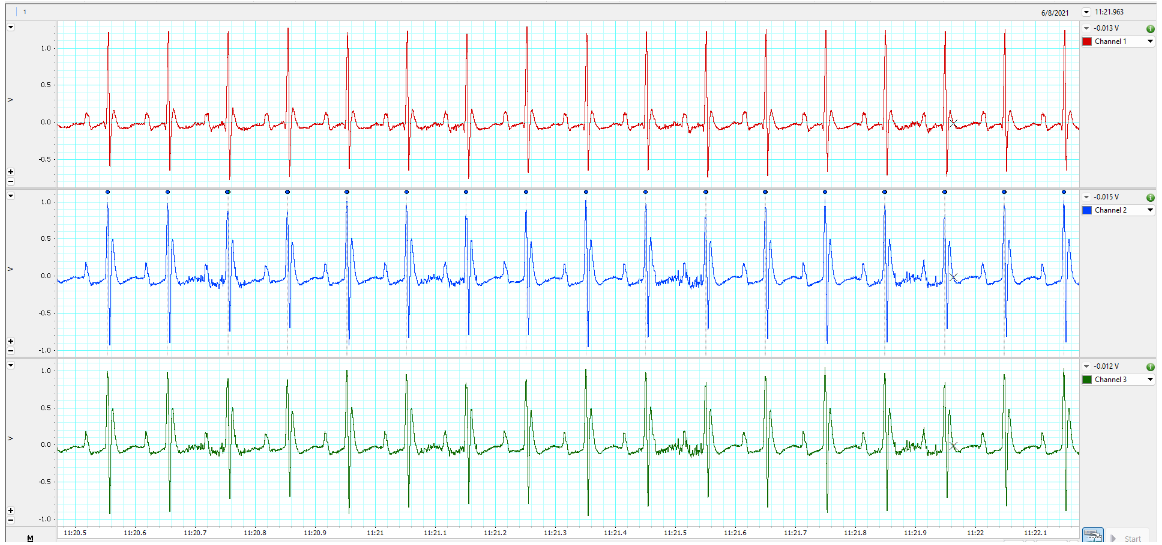

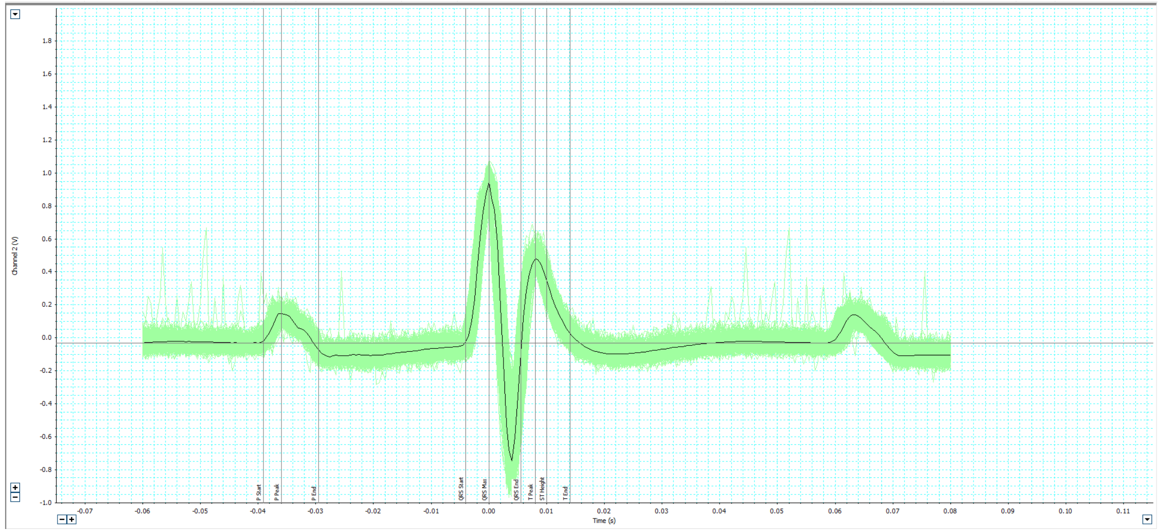

Genotype: Nos1ap$^{fl/fl}$; +/αMHC-MCM; Sex: Female; ECG: Anesthetized; Isoproterenol 5mg/kg

Baseline

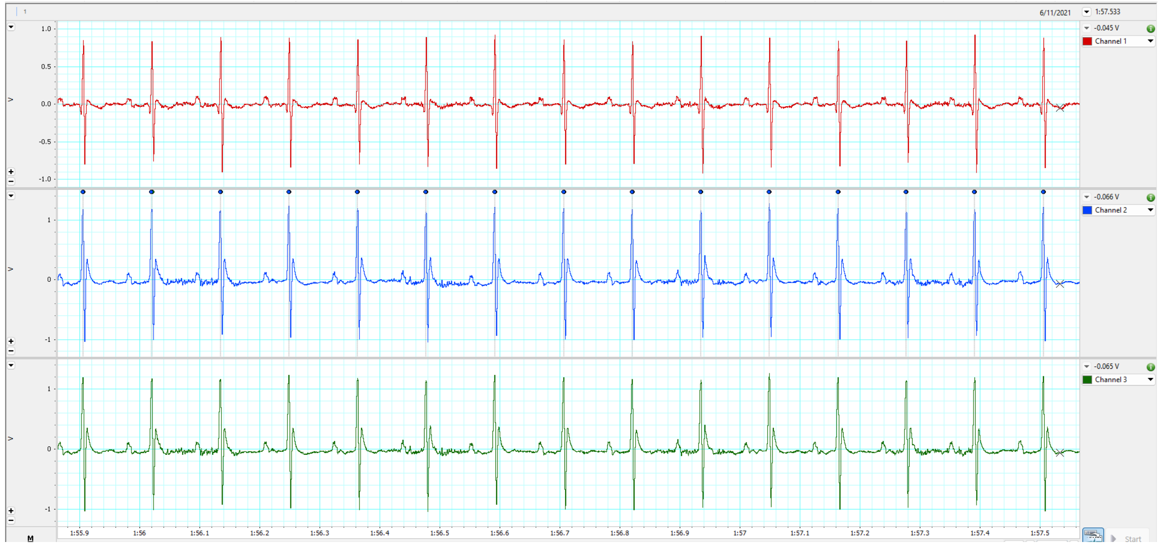

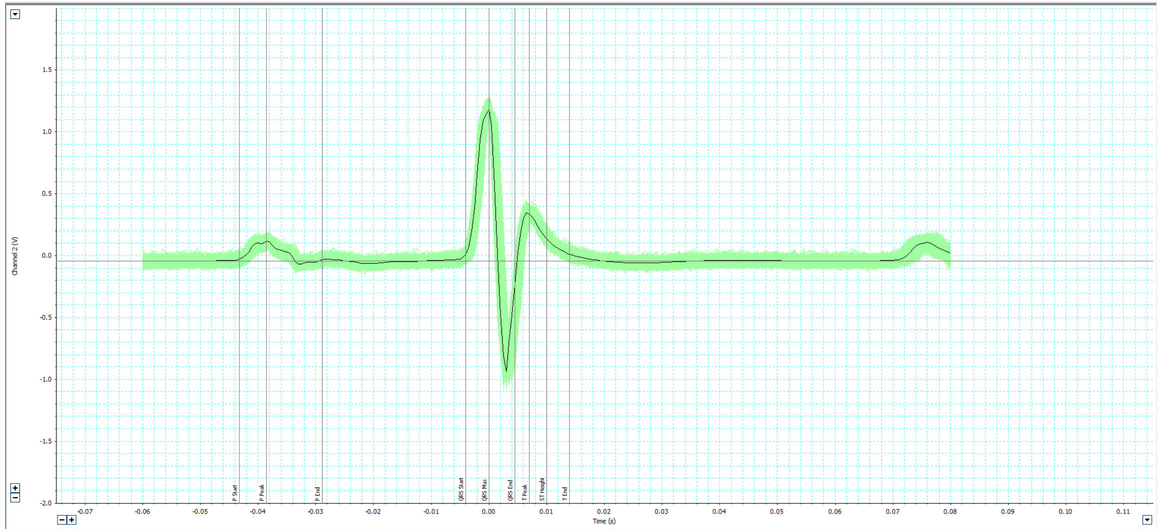

Drug

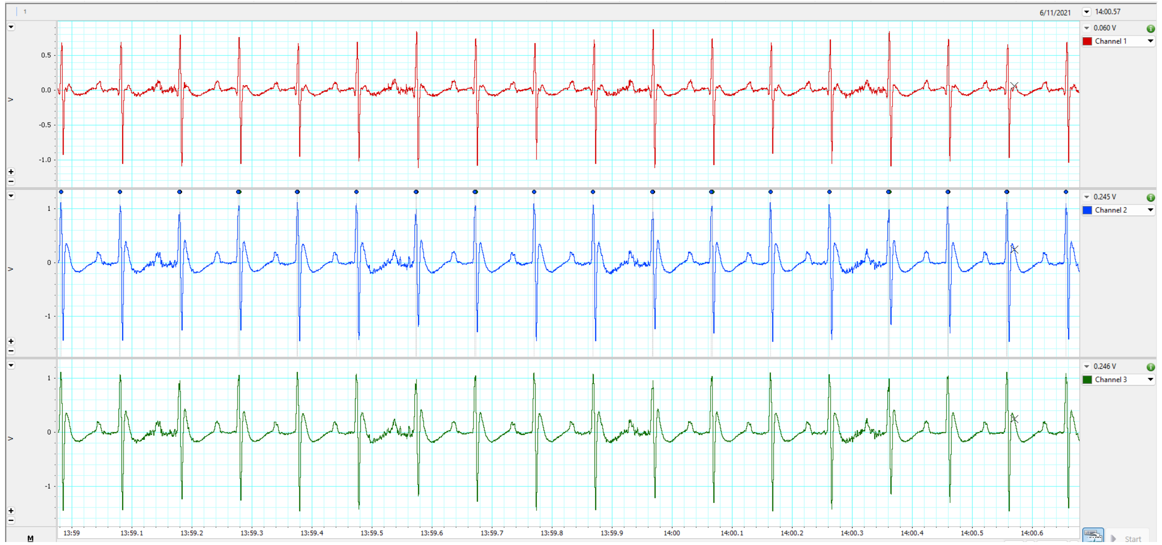

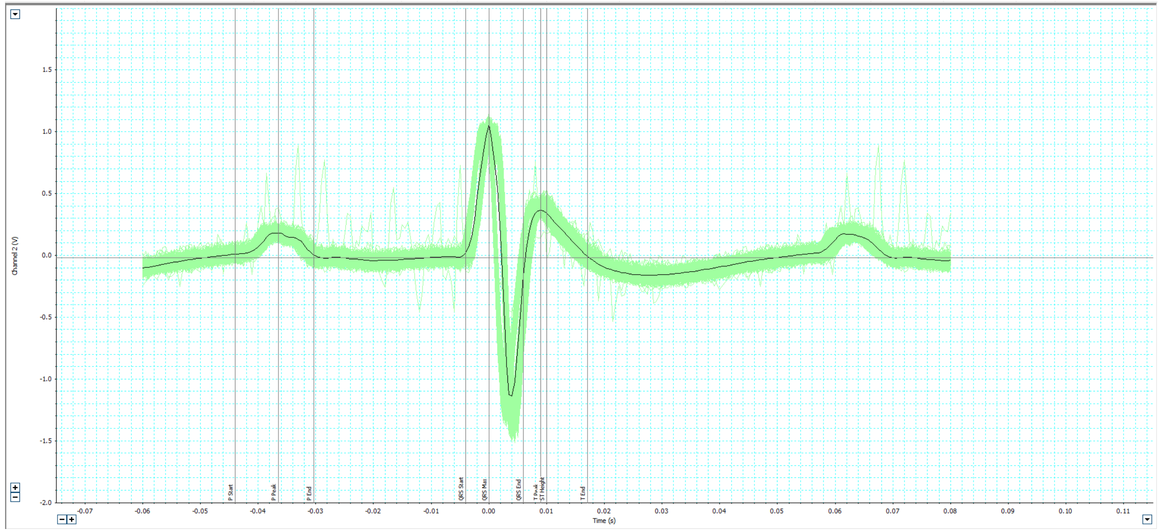

Supplement: jkad208_Supplementary_Data [file jkad208_supplementary_data.zip › Dataset_S2_G3-2023-404487.pdf]
