# Supplementary material for: Cardiac muscle–restricted partial loss of Nos1ap expression has limited but significant impact on electrocardiographic features
Source: G3 (Bethesda). 2023 Sep 14;13(11):jkad208. doi: 10.1093/g3journal/jkad208 (PMC10627271; doi:10.1093/g3journal/jkad208)
Supplement: jkad208_Supplementary_Data [file jkad208_supplementary_data.zip › Supplementary_Figures_and_Tables_G3-2023-404487.pdf]

Supplementary Information

Cardiac muscle-restricted partial loss of *Nos1ap* expression has limited but significant impact on electrocardiographic features

Alexa Smith, Dallas Auer, Morgan Johnson, Ernesto Sanchez, Holly Ross, Christopher Ward, Aravinda Chakravarti, Ashish Kapoor

Ashish Kapoor
Email: ashish.kapoor@uth.tmc.edu

Aravinda Chakravarti
Email: aravinda.chakravarti@nyulangone.org

This PDF file includes:

Figures S1 to S7
Tables S1 to S14

Other supplementary materials for this manuscript include the following:

Dataset S1
Dataset S2
Dataset S3

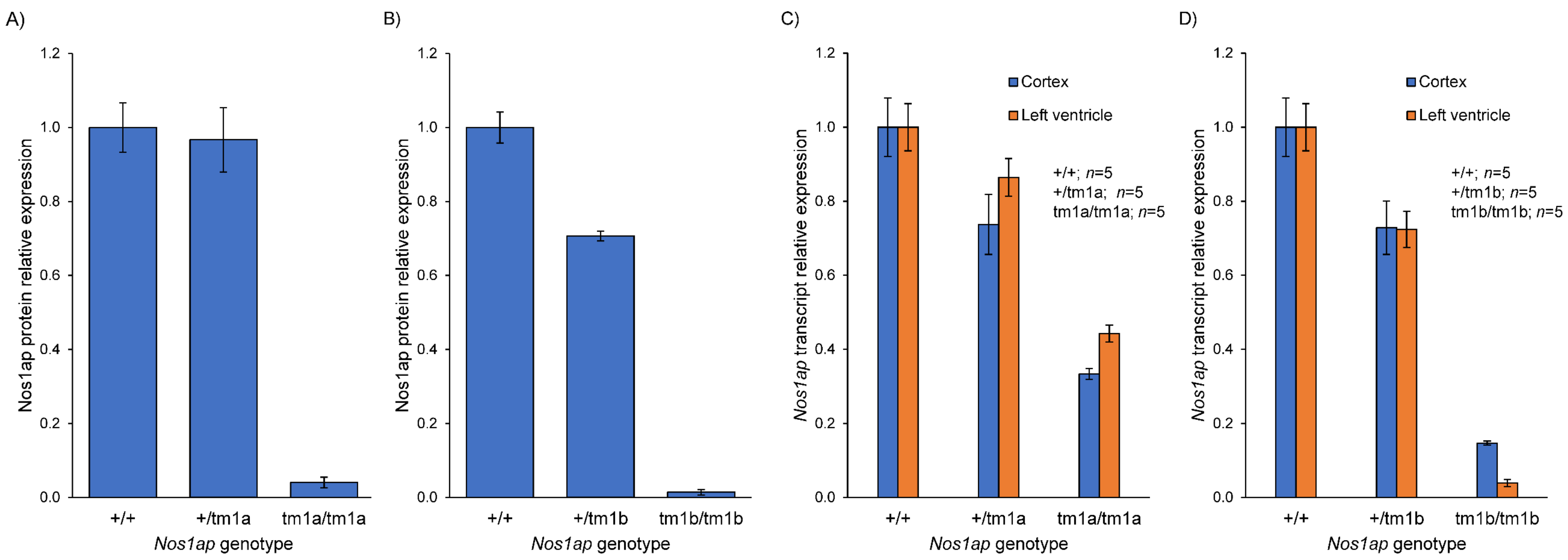

Figure S1: Reduced *Nos1ap* expression in targeted (tm1a) and derived (tm1b) allele carriers. (*A*) and (*B*) Relative quantification of of Nos1ap protein bands shown in Figure 2A. Compared to wildtype mice (+/+), *Nos1ap* tm1a (*A*) homozygotes and tm1b (*B*) carriers and homozygotes have reduced Nos1ap protein expression in adult brain cortex tissue. *P* values for wildtype to heterozygote comparisons: 0.77 (tm1a) and 0.003 (tm1b). *P* values for wildtype to homozygote comparisons: 1.5×10^{-4} (tm1a) and 2.1×10^{-5} (tm1b). (*C*) and (*D*) *Nos1ap* transcript relative expression in *Nos1ap* tm1a (*C*) and tm1b (*D*) mice. Compared to wildtype mice (+/+), *Nos1ap* tm1a (*C*) and tm1b (*D*) carriers and homozygotes have reduced *Nos1ap* transcript expression in adult brain cortex and left ventricle tissues. *P* values for wildtype to heterozygote comparisons: 0.05 (tm1a cortex), 0.13 (tm1a left ventricle), 0.03 (tm1b cortex), and 0.01 (tm1b left ventricle). *P* values for wildtype to homozygte comparisons: 3.4×10^{-5} (tm1a cortex), 3.5×10^{-5} (tm1a left ventricle), 5.0×10^{-6} (tm1b cortex), 3.9×10^{-7} (tm1b left ventricle). Error bars: SEM.

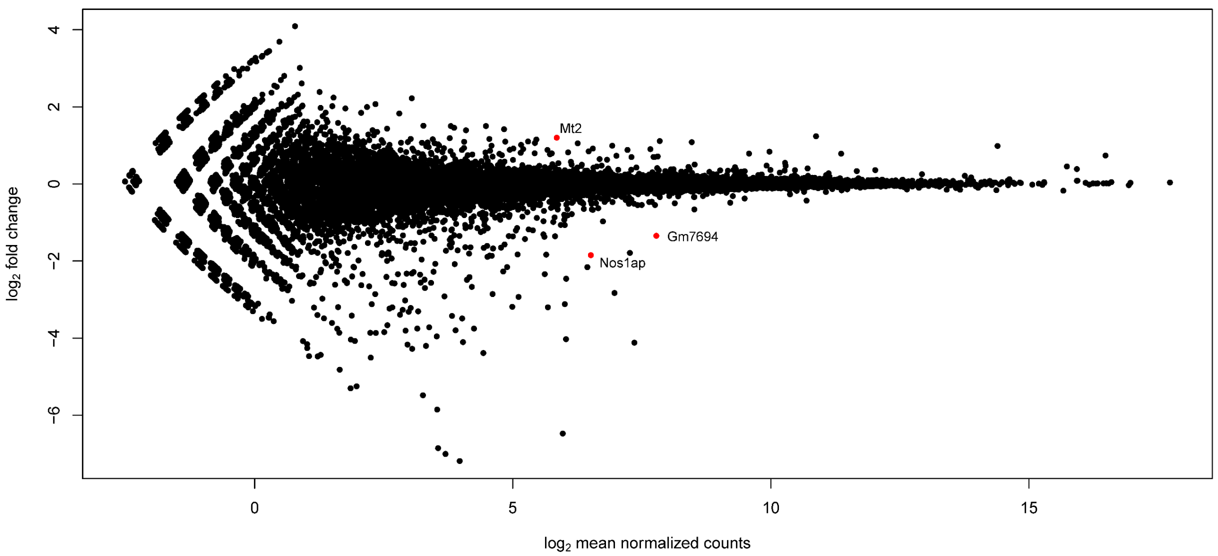

Figure S2: Loss of *Nos1ap* expression has no major effect on E13.5 heart transcriptome. Volcano plot showing $\log_2$ mean normalized read counts (X-axis) and $\log_2$ fold change (Y-axis) comparing the E13.5 heart transcriptome of *Nos1ap*$^{-/-}$ to wildtype mice. Each dot represents a gene. Red dots indicate the three genes that are differentially expressed in mutant mice with FDR <1% and absolute $\log_2$ fold change >1, and black dots indicate other genes.

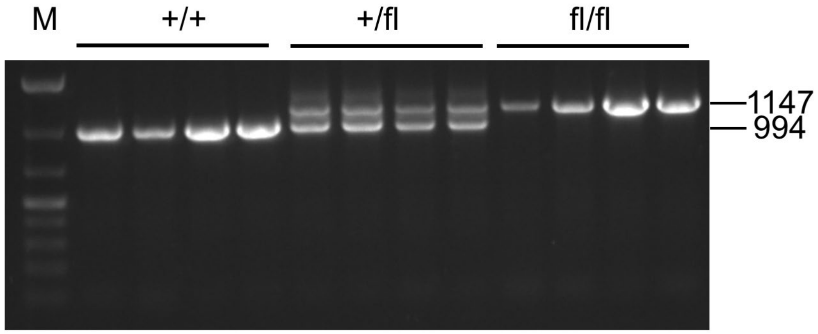

Figure S3: Absence of tamoxifen-inducible Cre-recombinase-based excision of the *Nos1ap* floxed allele in tail tissue of αMHC-MerCreMer mice. Post tamoxifen intraperitoneal injections, PCR amplification of the *Nos1ap* locus using tail tissue genomic DNA from *Nos1ap*$^{+/+}$, *Nos1ap*$^{+/fl}$ and *Nos1ap*$^{fl/fl}$ mice, all with tamoxifen-inducible αMHC-MerCreMer transgene, shows absence of excision. M: DNA ladder.

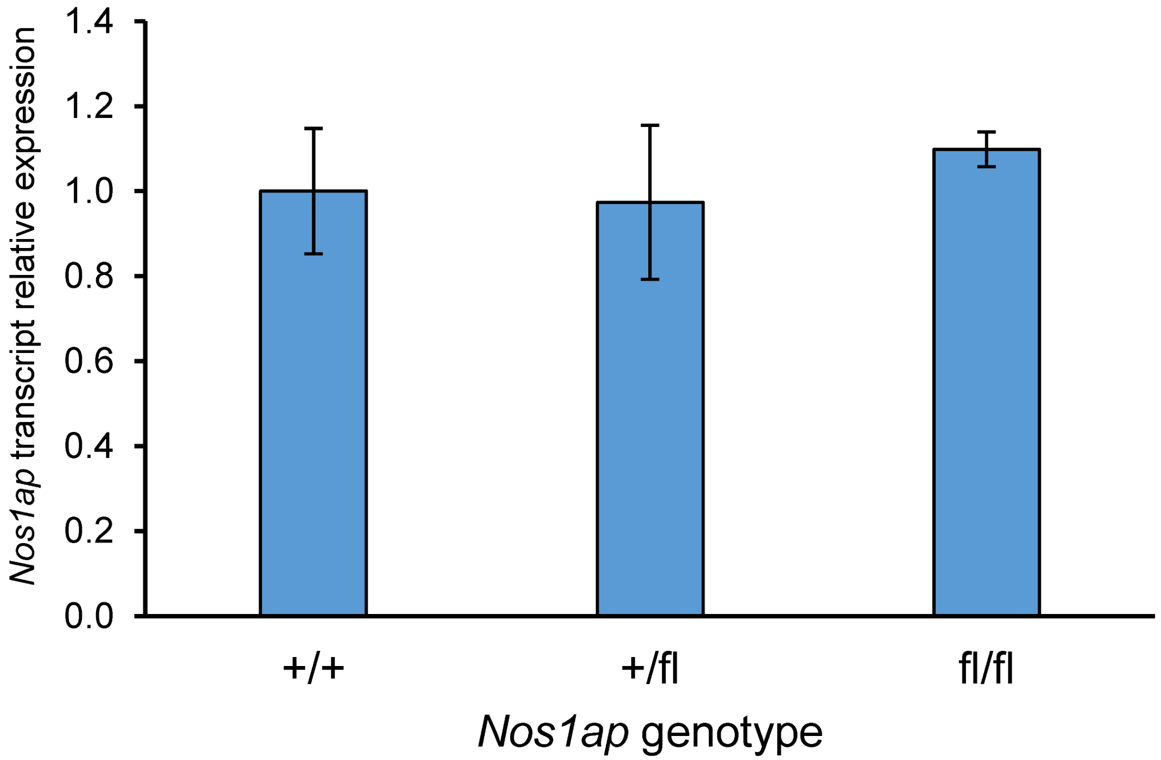

Figure S4: No significant differences in *Nos1ap* left ventricle gene expression in αMHC-MerCreMer-negative *Nos1ap* floxed allele carriers and homozygotes. In the absence of αMHC-MerCreMer, compared to wildtype mice (+/+), *Nos1ap* floxed (fl) allele carriers and homozygotes have no significant difference in *Nos1ap* expression in left ventricle tissue. Error bars: SEM; n=6 for each genotype.

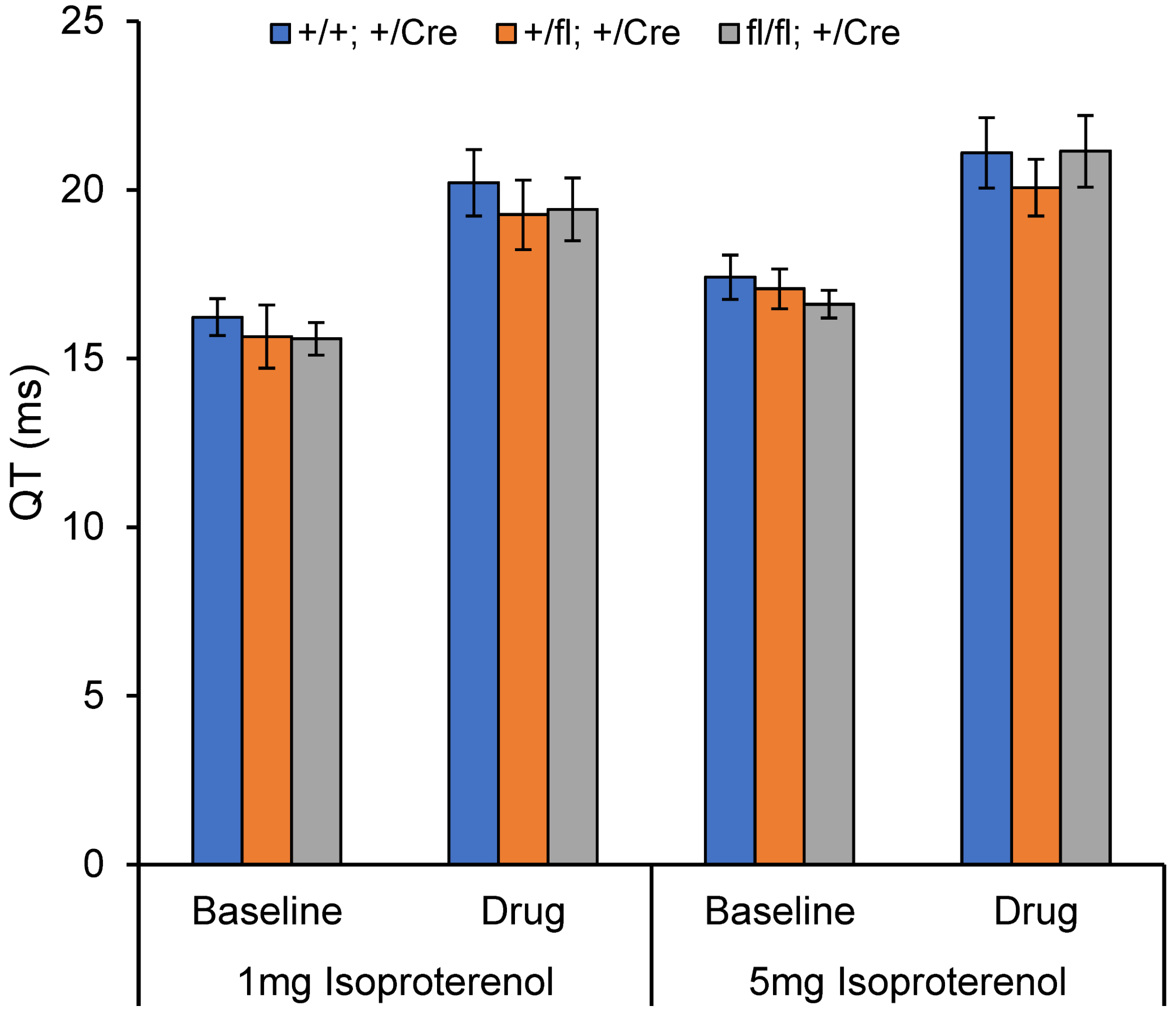

Figure S5: No significant differences in QT interval in anesthetized ECG measurements in *Nos1ap*$^{+/+}$, *Nos1ap*$^{+/fl}$ and *Nos1ap*$^{fl/fl}$ mice with αMHC-MerCreMer. QT interval from ECG recording under anesthesia at baseline and after injecting 1mg/kg or 5mg/kg body weight doses of isoproterenol in *Nos1ap*$^{+/+}$, *Nos1ap*$^{+/fl}$ and *Nos1ap*$^{fl/fl}$ mice, all with tamoxifen-inducible αMHC-MerCreMer transgene, shows no significant difference across genotypes. Error bars: SEM.

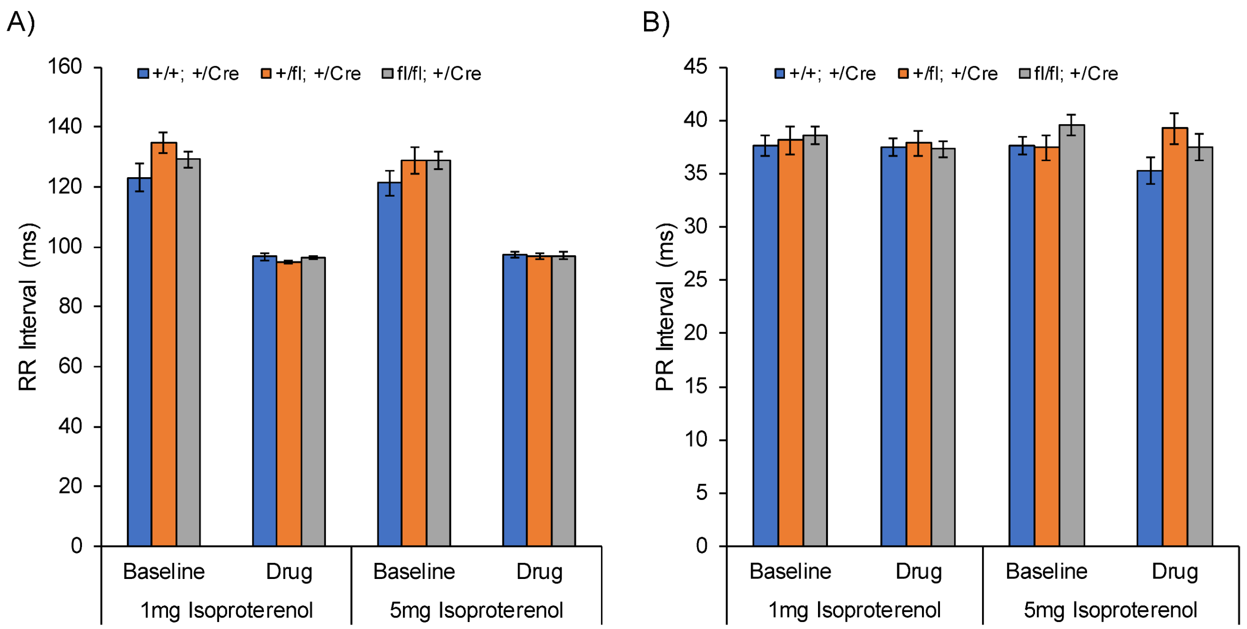

Figure S6: No significant differences in RR interval and PR interval in anesthetized ECG measurements in *Nos1ap*$^{+/+}$, *Nos1ap*$^{+/fl}$ and *Nos1ap*$^{fl/fl}$ mice with αMHC-MerCreMer. RR interval (*A*) and PR interval (*B*) observed in surface ECG recordings of anesthetized mice at baseline and after injection of isoproterenol (1mg/kg or 5mg/kg dose). Error bars: SEM.

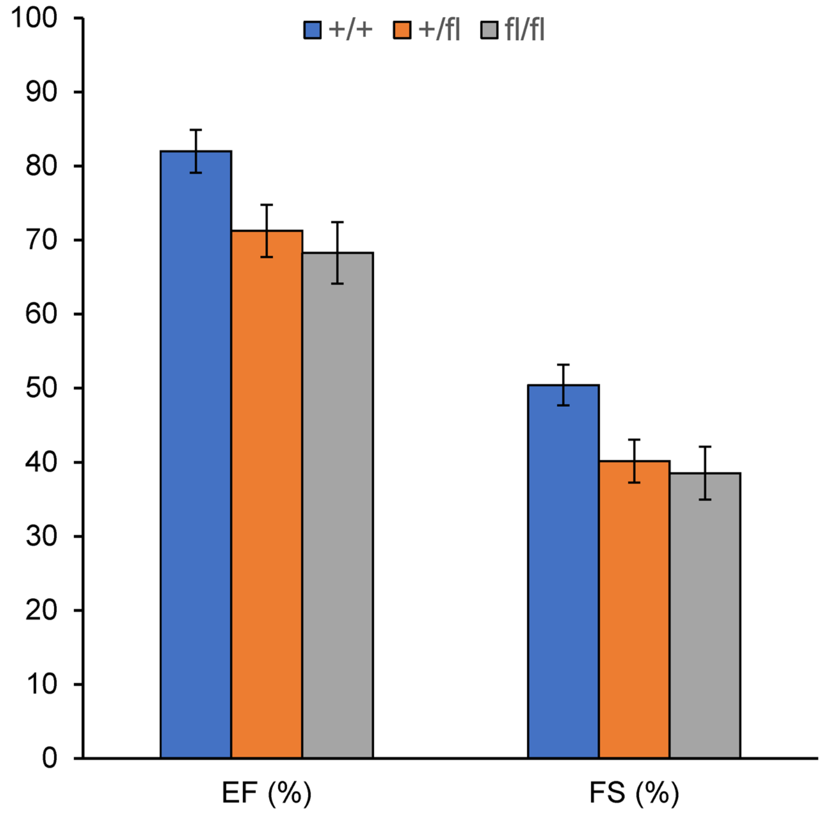

Figure S7: *Nos1ap* floxed allele carriers with αMHC-MerCreMer trend towards reduced left ventricular function. Compared to wildtype mice, (+/+), *Nos1ap* floxed (fl) allele carrier and homozygotes show reduced ejection fraction (EF) and fractional shortening (FS) by echocardiography. *P* values for wildtype to heterozygote comparisons: 0.032 (EF) and 0.020 (FS). *P* values for wildtype to homozygote comparisons: 0.014 (EF) and 0.016 (FS). Error bars: SEM.

Table S1: Primers for PCR genotyping

Name	Sequence (5'-3')	Notes	Locus
Nos1apIntron3-5'F	TGTGACTCTTGCTACGGAAAGTGG	Maps to 5' homology arm	*Nos1ap*
Common-En2-R	CCAACTGACCTTGGGCAAGAACAT	Maps to En2 splice acceptor site	
Common-loxP-F	GAGATGGCGCAACGCAATTAATG	Maps upstream of 3' loxP in the critical region	
Nos1apIntron4-3'R	AGACAGTGACTGGGGAATTGGAAG	Maps to 3' homology arm	
Cre-F	GCGGTCTGGCAGTAAAAACTATC	102 bp transgene-specific amplicon	CMV-Cre
Cre-R	GTGAAACAGCATTGCTGTCACTT		
Flpe-F	TGCCGGTCCTATTTACTCGT	100 bp transgene-specific amplicon	ACTB-Flpe
Flpe-R	TACTTCTTTAGCGCAAGGGGTAG		
A1cf-common-F	TCTATTGCACACAGCAATCCA	300 bp and 420 bp amplicons from wildtype and transgene alleles, respectively	αMHC-MerCreMer
A1cf-WT-R	CCAACTCTTGTGAGAGGAGCA		
MHC-MCM-R	CAAAGGCTCCAGGTCTGAGG		

Table S2: Alleles and PCR amplicon sizes (in bp)

	Primer combinations		
**Allele**	Nos1apIntron3-5'F Common-En2-R	Common-loxP-F Nos1apIntron4-3'R	Nos1apIntron3-5'F Nos1apIntron4-3'R
wildtype	x	x	994
tm1a	340	193	8051*
tm1b	340	x	5368*
tm1c or fl	x	193	1147
tm1d or -	x	x	375

x: no amplification expected; *not observed under the optimized thermal cycling conditions.

Table S3: Genotype distributions from crosses between *Nos1ap*$^{+/fl}$; +/+ and *Nos1ap*$^{+/fl}$; +/*Tg*$^{\alpha MHC\text{-}MerCreMer}$ mice

**Genotype**	**Observed (%)**	**Expected[1] (%)**	χ^2	***P***
Nos1ap$^{+/+}$; +/+	14 (11.4)	15 (12.5)	2.69	0.75
Nos1ap$^{+/fl}$; +/+	26 (21.1)	31 (25.0)		
Nos1ap$^{fl/fl}$; +/+	17 (13.8)	31 (12.5)		
Nos1ap$^{+/+}$; +/*Tg*$^{\alpha MHC\text{-}MerCreMer}$	17 (13.8)	15 (12.5)		
Nos1ap$^{+/fl}$; +/*Tg*$^{\alpha MHC\text{-}MerCreMer}$	29 (23.6)	31 (25.0)		
Nos1ap$^{fl/fl}$; +/*Tg*$^{\alpha MHC\text{-}MerCreMer}$	20 (16.3)	15 (12.5)		

[1]rounded-off to single significant digit.

Table S4: Counts of animals per genotype and time-point undergoing awake ECG measurement

	Time-point (weeks)															
**Genotype**	**6**	**8**	**10**	**12**	**14**	**16**	**18**	**20**	**22**	**24**	**28**	**32**	**36**	**48**	**44**	**48**
Nos1ap$^{+/+}$; +/αMHC-MCM	15	15	14	14	12	11	11	11	11	11	11	11	11	11	11	11
Nos1ap$^{+/fl}$; +/αMHC-MCM	23	23	22	21	19	17	16	15	14	14	14	10	10	10	10	10
Nos1ap$^{fl/fl}$; +/αMHC-MCM	16	16	14	14	13	12	12	12	12	12	11	12	12	12	12	12
Nos1ap$^{+/+}$; +/+	13	13	13	10	10	10	10	10	10	10						
Nos1ap$^{+/fl}$; +/+	26	26	24	23	23	21	18	17	16	13						
Nos1ap$^{fl/fl}$; +/+	13	14	12	12	11	10	10	10	10	9						

Table S5: Age-, sex- and genotype-dependent effects on heart rate-corrected QT interval observed in awake ECG measurement in *Nos1ap*$^{+/+}$, *Nos1ap*$^{+/fl}$ and *Nos1ap*$^{fl/fl}$ mice with αMHC-MerCreMer

**Regression Statistics**						
Multiple R	0.27					
R Square	0.07					
Adjusted R Square	0.07					
Standard Error	1.88					
Observations	1989					
**ANOVA**	df	SS	MS	F	Significance F	
Regression	4	556.23	139.06	39.41	8.55E-32	
Residual	1984	7000.82	3.53			
Total	1988	7557.04				
**Predictors**	Coefficients	Standard Error	t Stat	P-value	Lower 95%	Upper 95%
Intercept	45.70	0.11	403.71	0	45.47	45.92
Age weeks	0.03	0.00	9.43	1.09E-20	0.03	0.04
Sex (female)	-0.66	0.08	-7.83	7.71E-15	-0.83	-0.50
(fl+)	0.11	0.10	1.09	0.275	-0.09	0.32
(fl/fl)	0.34	0.11	3.18	0.002	0.13	0.55

Table S6: Age-, sex- and genotype-dependent effects on QT interval observed in awake ECG measurement in *Nos1ap*$^{+/+}$, *Nos1ap*$^{+/fl}$ and *Nos1ap*$^{fl/fl}$ mice with αMHC-MerCreMer

**Regression Statistics**						
Multiple R	0.24					
R Square	0.06					
Adjusted R Square	0.06					
Standard Error	1.75					
Observations	1989					
**ANOVA**	Df	SS	MS	F	Significance F	
Regression	4	385.08	96.27	31.42	2.07E-25	
Residual	1984	6078.38	3.06			
Total	1988	6463.46				
**Predictors**	Coefficients	Standard Error	t Stat	P-value	Lower 95%	Upper 95%
Intercept	40.60	0.11	384.91	0	40.39	40.80
Age weeks	0.03	0.00	8.88	1.49E-18	0.02	0.03
Sex (female)	-0.38	0.08	-4.80	1.67E-06	-0.53	-0.22
(fl+)	-0.16	0.10	-1.68	0.093	-0.35	0.03
(fl/fl)	0.25	0.10	2.52	0.012	0.06	0.45

Table S7: Age-, sex- and genotype-dependent effects on heart rate-corrected QT interval observed in awake ECG measurement in *Nos1ap*$^{+/+}$, *Nos1ap*$^{+/fl}$ and *Nos1ap*$^{fl/fl}$ mice without αMHC-MerCreMer

**Regression Statistics**						
Multiple R	0.18					
R Square	0.03					
Adjusted R Square	0.03					
Standard Error	1.97					
Observations	1345					
**ANOVA**	df	SS	MS	F	Significance F	
Regression	4	181.95	45.49	11.75	2.21E-09	
Residual	1340	5187.53	3.87			
Total	1344	5369.48				
**Predictors**	Coefficients	Standard Error	t Stat	P-value	Lower 95%	Upper 95%
Intercept	45.03	0.18	248.84	0	44.68	45.39
Age weeks	0.04	0.01	4.17	3.30E-05	0.02	0.06
Sex (female)	-0.19	0.11	-1.74	0.083	-0.40	0.02
(fl/+)	0.66	0.13	5.04	5.19E-07	0.40	0.92
(fl/fl)	0.63	0.15	4.19	2.98E-05	0.34	0.93

Table S8: Age-, sex- and genotype-dependent effects on QT interval observed in awake ECG measurement in *Nos1ap*$^{+/+}$, *Nos1ap*$^{+/fl}$ and *Nos1ap*$^{fl/fl}$ mice without αMHC-MerCreMer

**Regression Statistics**						
Multiple R	0.14					
R Square	0.02					
Adjusted R Square	0.02					
Standard Error	1.90					
Observations	1345					
**ANOVA**	df	SS	MS	F	Significance F	
Regression	4	90.87	22.72	6.31	4.95E-05	
Residual	1340	4822.66	3.60			
Total	1344	4913.53				
**Predictors**	Coefficients	Standard Error	t Stat	P-value	Lower 95%	Upper 95%
Intercept	40.24	0.17	230.58	0	39.89	40.58
Age weeks	0.03	0.01	3.15	0.002	0.01	0.05
Sex (female)	-0.19	0.11	-1.81	0.070	-0.40	0.02
(fl/+)	0.37	0.13	2.97	0.003	0.13	0.62
(fl/fl)	0.48	0.15	3.32	9.22E-04	0.20	0.77

Table S9: Drug (1mg/kg isoproterenol) exposure-, sex- and genotype-dependent effects on heart-rate corrected QT interval observed in anesthetized ECG measurement in *Nos1ap*$^{+/+}$, *Nos1ap*$^{+/fl}$ and *Nos1ap*$^{fl/fl}$ mice with αMHC-MerCreMer

**Regression Statistics**						
Multiple R	0.80					
R Square	0.63					
Adjusted R Square	0.60					
Standard Error	2.47					
Observations	54					
**ANOVA**	df	SS	MS	F	Significance F	
Regression	4	516.08	129.02	21.15	3.52E-10	
Residual	49	298.95	6.10			
Total	53	815.02				
**Predictors**	Coefficients	Standard Error	t Stat	P-value	Lower 95%	Upper 95%
Intercept	14.18	0.70	20.21	2.03E-25	12.77	15.58
Drug exposure	6.01	0.67	8.94	7.34E-12	4.66	7.36
Sex (female)	1.16	0.68	1.71	0.09	-0.20	2.53
(fl/+)	-1.03	0.86	-1.20	0.24	-2.76	0.70
(fl/fl)	-1.00	0.78	-1.28	0.21	-2.58	0.57

Table S10: Drug (1mg/kg isoproterenol) exposure-, sex- and genotype-dependent effects on QT interval observed in anesthetized ECG measurement in *Nos1ap*$^{+/+}$, *Nos1ap*$^{+/fl}$ and *Nos1ap*$^{fl/fl}$ mice with αMHC-MerCreMer

**Regression Statistics**						
Multiple R	0.67					
R Square	0.45					
Adjusted R Square	0.41					
Standard Error	2.36					
Observations	54					
**ANOVA**	df	SS	MS	F	Significance F	
Regression	4	227.41	56.85	10.20	4.36E-06	
Residual	49	273.19	5.58			
Total	53	500.60				
**Predictors**	Coefficients	Standard Error	t Stat	P-value	Lower 95%	Upper 95%
Intercept	15.79	0.67	23.55	2.27E-28	14.44	17.13
Drug exposure	3.84	0.64	5.97	2.62E-07	2.54	5.13
Sex (female)	1.29	0.65	1.99	0.05	-0.01	2.59
(fl/+)	-0.80	0.82	-0.97	0.34	-2.45	0.85
(fl/fl)	-0.85	0.75	-1.13	0.26	-2.35	0.66

Table S11: Drug (5mg/kg isoproterenol) exposure-, sex- and genotype-dependent effects on heart-rate corrected QT interval observed in anesthetized ECG measurement in *Nos1ap*$^{+/+}$, *Nos1ap*$^{+/fl}$ and *Nos1ap*$^{fl/fl}$ mice with αMHC-MerCreMer

**Regression Statistics**						
Multiple R	0.78					
R Square	0.61					
Adjusted R Square	0.58					
Standard Error	2.60					
Observations	60					
**ANOVA**	df	SS	MS	F	Significance F	
Regression	4	585.68	146.42	21.68	8.83E-11	
Residual	55	371.41	6.75			
Total	59	957.09				
**Predictors**	Coefficients	Standard Error	t Stat	P-value	Lower 95%	Upper 95%
Intercept	14.88	0.72	20.76	1.07E-27	13.44	16.31
Drug exposure	5.97	0.67	8.90	3.02E-12	4.63	7.32
Sex (female)	1.70	0.67	2.52	0.01	0.35	3.05
(fl/+)	-0.85	0.89	-0.96	0.34	-2.63	0.93
(fl/fl)	-0.64	0.77	-0.84	0.41	-2.18	0.90

Table S12: Drug (5mg/kg isoproterenol) exposure-, sex- and genotype-dependent effects on QT interval observed in anesthetized ECG measurement in *Nos1ap*$^{+/+}$, *Nos1ap*$^{+/fl}$ and *Nos1ap*$^{fl/fl}$ mice with αMHC-MerCreMer

**Regression Statistics**						
Multiple R	0.67					
R Square	0.45					
Adjusted R Square	0.41					
Standard Error	2.48					
Observations	60					
**ANOVA**	df	SS	MS	F	Significance F	
Regression	4	277.70	69.42	11.25	9.73E-07	
Residual	55	339.53	6.17			
Total	59	617.23				
**Predictors**	Coefficients	Standard Error	t Stat	P-value	Lower 95%	Upper 95%
Intercept	16.50	0.68	24.08	6.78E-31	15.12	17.87
Drug exposure	3.87	0.64	6.03	1.45E-07	2.58	5.15
Sex (female)	1.82	0.64	2.82	0.007	0.53	3.11
(fl/+)	-0.64	0.85	-0.76	0.45	-2.35	1.06
(fl/fl)	-0.46	0.73	-0.63	0.53	-1.93	1.01

Table S13: Drug (1mg/kg isoproterenol) exposure-, sex- and genotype-dependent effects on QRS interval observed in anesthetized ECG measurement in *Nos1ap*$^{+/+}$, *Nos1ap*$^{+/fl}$ and *Nos1ap*$^{fl/fl}$ mice with αMHC-MerCreMer

**Regression Statistics**						
Multiple R	0.67					
R Square	0.45					
Adjusted R Square	0.41					
Standard Error	0.93					
Observations	54					
**ANOVA**	df	SS	MS	F	Significance F	
Regression	4	34.76	8.69	10.08	4.94E-06	
Residual	49	42.26	0.86			
Total	53	77.02				
**Predictors**	Coefficients	Standard Error	t Stat	P-value	Lower 95%	Upper 95%
Intercept	8.62	0.26	32.69	6.18E-35	8.09	9.15
Drug exposure	1.46	0.25	5.76	5.45E-07	0.95	1.96
Sex (female)	-0.14	0.26	-0.56	0.58	-0.65	0.37
(fl/+)	-0.30	0.32	-0.93	0.36	-0.95	0.35
(fl/fl)	-0.75	0.29	-2.54	0.01	-1.34	-0.16

Table S14: Drug (5mg/kg isoproterenol) exposure-, sex- and genotype-dependent effects on QRS interval observed in anesthetized ECG measurement in *Nos1ap*$^{+/+}$, *Nos1ap*$^{+/fl}$ and *Nos1ap*$^{fl/fl}$ mice with αMHC-MerCreMer

**Regression Statistics**						
Multiple R	0.68					
R Square	0.46					
Adjusted R Square	0.43					
Standard Error	0.92					
Observations	60					
**ANOVA**	df	SS	MS	F	Significance F	
Regression	4	40.81	10.20	11.93	4.75E-07	
Residual	55	47.02	0.85			
Total	59	87.83				
**Predictors**	Coefficients	Standard Error	t Stat	P-value	Lower 95%	Upper 95%
Intercept	8.51	0.25	33.40	3.30E-38	8.00	9.02
Drug exposure	1.44	0.24	6.04	1.41E-07	0.96	1.92
Sex (female)	-0.16	0.24	-0.67	0.51	-0.64	0.32
(fl/+)	0.03	0.32	0.09	0.93	-0.61	0.66
(fl/fl)	-0.78	0.27	-2.87	0.006	-1.33	-0.24
